# Single‐cell RNA sequencing of motoneurons identifies regulators of synaptic wiring in *Drosophila* embryos

**DOI:** 10.15252/msb.202110255

**Published:** 2022-02-28

**Authors:** Jessica Velten, Xuefan Gao, Patrick Van Nierop y Sanchez, Katrin Domsch, Rashi Agarwal, Lena Bognar, Malte Paulsen, Lars Velten, Ingrid Lohmann

**Affiliations:** ^1^ Department of Developmental Biology Centre for Organismal Studies (COS) Heidelberg Heidelberg Germany; ^2^ The Barcelona Institute of Science and Technology Centre for Genomic Regulation (CRG) Barcelona Spain; ^3^ Flow Cytometry Core Facility European Molecular Biology Laboratory (EMBL) Heidelberg Germany; ^4^ Developmental Biology Erlangen‐Nürnberg University Erlangen Germany; ^5^ Universitat Pompeu Fabra (UPF) Barcelona Spain

**Keywords:** circuit wiring, *Drosophila* embryonic motoneuron, homeodomain transcription factors, Ig‐domain encoding proteins, single‐cell RNA sequencing, Chromatin, Transcription & Genomics, Neuroscience

## Abstract

The correct wiring of neuronal circuits is one of the most complex processes in development, since axons form highly specific connections out of a vast number of possibilities. Circuit structure is genetically determined in vertebrates and invertebrates, but the mechanisms guiding each axon to precisely innervate a unique pre‐specified target cell are poorly understood. We investigated *Drosophila* embryonic motoneurons using single‐cell genomics, imaging, and genetics. We show that a cell‐specific combination of homeodomain transcription factors and downstream immunoglobulin domain proteins is expressed in individual cells and plays an important role in determining cell‐specific connections between differentiated motoneurons and target muscles. We provide genetic evidence for a functional role of five homeodomain transcription factors and four immunoglobulins in the neuromuscular wiring. Knockdown and ectopic expression of these homeodomain transcription factors induces cell‐specific synaptic wiring defects that are partly phenocopied by genetic modulations of their immunoglobulin targets. Taken together, our data suggest that homeodomain transcription factor and immunoglobulin molecule expression could be directly linked and function as a crucial determinant of neuronal circuit structure.

## Graphical abstract

Single‐cell RNA‐seq of *Drosophila* embryonic motoneurons combined with imaging and genetic perturbation suggests a linked homeodomain transcription factor – immunoglobulin program critical for synaptic wiring in the neuromuscular system.

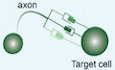

## Introduction

Neuronal circuits in mammals as well as *Drosophila* are stereotypically wired for the precise execution of functional tasks critical for organismal survival. The formation of such circuits is a step‐wise process, which starts with the specification of neuronal cell types and their accurate arrangements in space, followed by the correct wiring of individual cells and their final integration into a functional network. To ensure such precision, the structure and connectivity of neural circuits is genetically specified. However, how these complex interconnected processes are encoded in the genome and executed by the cellular protein machinery is still not fully understood.

According to the “labelled pathway hypothesis” (Sperry, 1963), neurons stochastically and transiently form contacts with many possible targets after their specification, while the expression of specific cell surface proteins (CSPs) is thought to stabilize the correct connections, a process called synaptic specificity (Sanes & Zipursky, 2020). Many lines of evidence support this hypothesis. Recent work has shown that combinations of immunoglobulin superfamily (IgSF) cell surface proteins (Dprs) are differentially expressed in distinct neuronal clusters and bind to specific Dpr binding proteins (DIPs) expressed in synaptic partners (Nakamura *et al*, 2002; Özkan *et al*, 2013; Carrillo *et al*, 2015). In the visual system, combinations of CSPs are differentially expressed between layers (Tan *et al*, 2015), while in olfactory neurons, a combinatorial expression of transcription factors (TFs) and CSPs maps neurons with the same olfactory receptor to the same glomerulus (Couto *et al*, 2005; Li *et al*, 2017, 2020; McLaughlin *et al*, 2021). All these studies explain how groups of similar neuronal cells are molecularly defined and provide a hypothesis on how stereotypic connections to another neuronal cell type are formed. By contrast, how the specificity of circuits is specified and controlled at the level of single cells is still not completely resolved.

In the *Drosophila* neuromuscular system, every single motoneuron (MN) forms unique and stereotypic connections with target muscles already during embryogenesis, but the molecular mechanisms underlying this specificity is unclear (Allan & Thor, 2015). MNs are progressively specified from anterior to posterior by segment‐specific TFs (Bossing *et al*, 1996; Schmidt *et al*, 1997; Angelini & Kaufman, 2005) and further along the dorsal to ventral axis (Broihier & Skeath, 2002; Broihier *et al*, 2004; Landgraf & Thor, 2006) before extending their nerve projections to predefined locations specified in all three spatial dimensions (Landgraf *et al*, 1997; Thor *et al*, 1999; Broihier & Skeath, 2002; Broihier *et al*, 2004; Zarin *et al*, 2014; Hessinger *et al*, 2016). These observations have suggested region‐specific mechanisms in the determination of connectivity patterns. However, such a regional model alone is unlikely to explain the precise connectivity patterns of single cells (Nassif *et al*, 1998; Landgraf *et al*, 2003). In addition, elegant studies in the vertebrate central nervous system and classical transplantation experiments demonstrated that positional identity and connectivity patterns of single neurons are stably maintained even after experimental relocation of cells (Demireva *et al*, 2011). Thus, there seems to be a molecular mechanism that stably imprints cellular identity and instructs the formation of cellular connectivity.

Homeodomain TFs have long been known to play important roles in the specification, differentiation and maintenance of neurons, also in MNs, in different organisms (Thor *et al*, 1999; Thor & Thomas, 2002; Urbach *et al*, 2006, 2016; Sanguinetto *et al*, 2008; Philippidou *et al*, 2012; Deneris & Hobert, 2014; Allan & Thor, 2015; Zeisel *et al*, 2018; Domsch *et al*, 2019; Sugino *et al*, 2019; Allen *et al*, 2020; Reilly *et al*, 2020). Importantly, it has been shown just recently that each neuron class in the nematode *C. elegans* expresses a unique combination of homeodomain TFs, which is unambiguously associated with neuronal identities (Reilly *et al*, 2020; Hobert, 2021). However, it is so far unclear whether this concept extends to other organisms; whether such cell‐specific combinations of homeodomain TFs also instruct later events in circuit formation; and, finally, which molecules downstream of such homeodomain TFs realize synaptic target choice and specificity at the single‐cell level.

Using single‐cell RNA sequencing (scRNA‐Seq) with high numbers of biological replicates, we demonstrate that cell‐specific expression of multiple homeodomain TFs is associated with the cellular heterogeneity within differentiated MNs along the major body axes of *Drosophila* embryos. We furthermore show that multiple CSPs, in particular cell surface immunoglobulins (Igs), act downstream of homeodomain TFs in individual MNs and play an important role in determining specificity during the synaptic wiring phase. Knockdown and ectopic expression of homeodomain TFs induces synaptic wiring defects specific to single cells that are partly phenocopied by genetic manipulation of their putative Ig targets. Additionally, our data suggest that shared combinations of homeodomain TF are expressed in matching synaptic partners of functional neuronal circuits. Based on these findings, we propose that the development of individual neuronal circuits is genetically defined by a linked “homeo‐immunoglobulin program”, which serves as one of the major determinants for complex neuronal wiring with single cell precision.

## Results

### A reference map of MNs during the synaptic wiring phase in *Drosophila* embryos

**Figure EV1:**
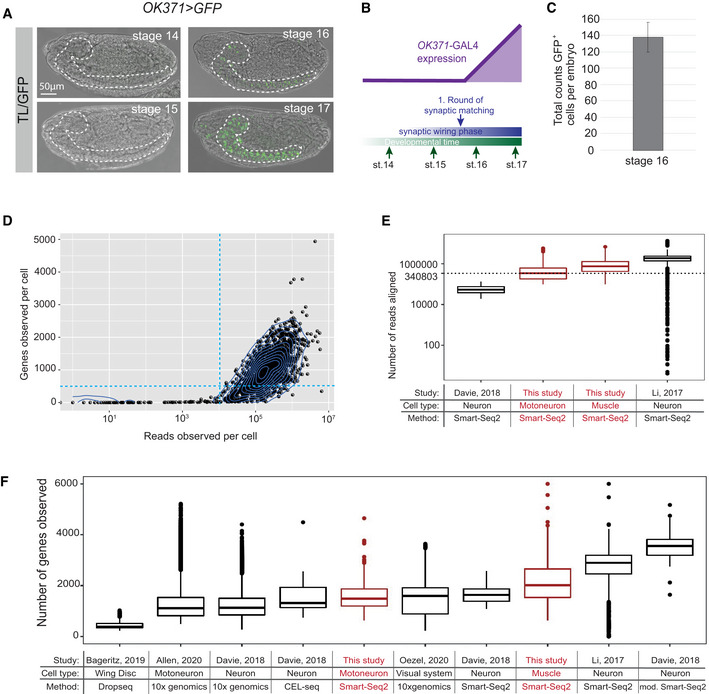
scRNA‐Seq data of embryonic *Drosophila* MNs are of high quality Time course of GFP expression induced by the *OK371‐GAL4* motoneuronal driver during embryonic stages, showing that GFP expression starts after embryonic stages 15 and is clearly detectable at stages 16 and 17 in neuronal cells. Dashed lines highlight the ventral nerve cord.Illustration of the time course shown in (A) in relation to the synaptic wiring in the embryonic neuromuscular system, highlighting that *OK371‐GAL4*‐driven transgene expression occurs at the time when the first synaptic connections are formed between MNs and muscles.Quantification of the average number of differentiated *OK371 > GFP*‐positive MNs in stage 16 embryos (*n* = 3, biological replicates with two independent biological repeats, error bar denotes standard deviation).Visualization of filtering criteria for single cells (dashed blue line, see Materials and Methods). Density dot plot represents the total reads (library size) versus genes observed per cell (library quality, diversity). Each dot represents a motoneuronal cell (total of 1,536 cells). In total, *n* = 999 cells passed the filtering criteria indicated by the dotted lines (see Materials and Methods).Number of reads aligned to the *Drosophila* genome per cell for the two datasets from this study (red) and two other studies (black) profiling *Drosophila* neurons by Smart‐Seq2. See Materials and Methods, section *Data visualization* for a definition of boxplot elements. Individual data points correspond to single cells (biological replicates), see legend of panel F for number of cells.Number of genes observed per cell for the two datasets from this study (red) and several other studies (black) profiling *Drosophila* neurons by scRNA‐Seq (Li *et al*, 2017; Davie *et al*, 2018; Bageritz *et al*, 2019; Allen *et al*, 2020; Özel *et al*, 2021). See Materials and Methods, section *Data visualization* for a definition of boxplot elements. Individual data points correspond to single cells (biological replicates). Bageritz *et al*: *n* = 2,554, Allen *et al*: *n* = 33,115, Davie 10× genomics: *n* = 56,902, Davie CEL‐seq: *n* = 22, Davie Smart‐Seq2: *n* = 45, Davie modified Smart‐Seq2: *n* = 34, this study (Motoneuron): *n* = 999, this study (Muscle): *n* = 837, Oezel *et al*: *n* = 31,018, Li *et al*: *n* = 1,842.

We aimed at identifying molecules driving specificity in synaptic wiring at the single‐cell level to gain a comprehensive view of the complex yet highly precise synaptic matching process. To this end, we used the *Drosophila* neuromuscular system as our model, as it is ideally suited to study mechanisms of synaptic specificity: first, this system is of relatively low complexity; and second, it is fully established at the end of embryogenesis with about 30‐35 MNs innervating in a highly stereotypic manner 30 muscles in each abdominal hemisegment of stage 17 embryos (Landgraf *et al*, 1997; Hoang & Chiba, 2001; Landgraf & Thor, 2006; Kim *et al*, 2009; Couton *et al*, 2015). Evidently, selection of the proper developmental stage is critical for the comprehensive identification of cues driving the highly specific interaction of neuronal cells. This notion is based on previous studies showing that neurons diversify most on the transcriptional level when they are in the process of contacting their synaptic partners while their transcriptomes become indistinguishable upon completion of neuronal connectivity (Li *et al*, 2017). In the *Drosophila* neuromuscular system, embryonic MNs interact with their muscle partners at the end of embryonic stage 16 (Landgraf *et al*, 1997), suggesting motoneuronal transcriptomes to be most diverse at this developmental stage. Based on these considerations, we performed scRNA‐Seq of stage 16 embryonic cells marked by the *OK371‐*GAL4 driver (Mahr & Aberle, 2006) controlling the expression of the UAS‐*RFP* transgene (Fig EV1A and B). This driver is based on a regulatory element controlling the expression of the presynaptic vesicular glutamate transporter (VGlut) and is active specifically in all/most MNs at late stages of embryogenesis (stage 17) (Mahr & Aberle, 2006) (Fig EV1A). In addition, the *OK371*‐GAL4 driver is active in a few glutamatergic brain neurons (Fig EV1A), which were later excluded by restricting the analysis to *Hox*‐expressing cells. We further confirmed that known motoneuronal subtypes are targeted by this driver in expected ratios in stage 16 embryos (Fig EV3A–D, and see below) (Mahr & Aberle, 2006).

**Figure EV2:**
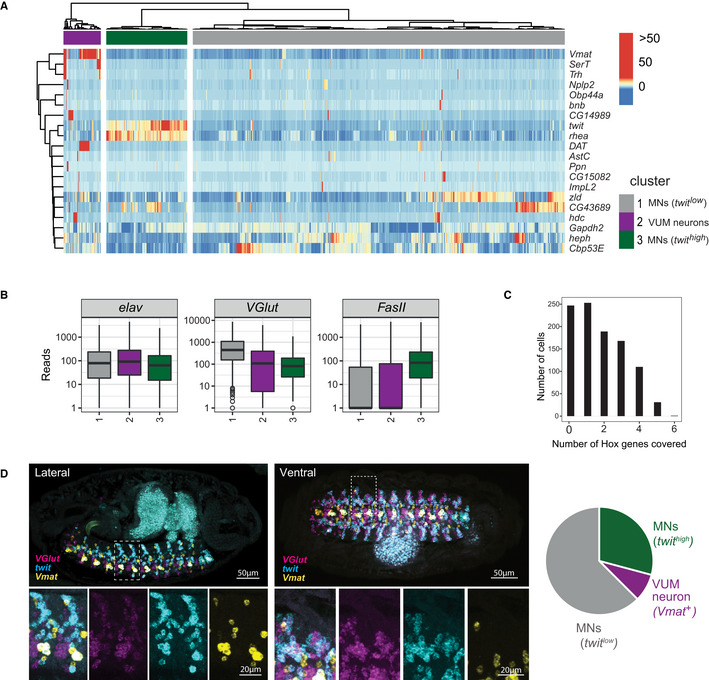
scRNA‐Seq data of embryonic *Drosophila* MNs identifies three major clusters Heatmap depicting gene expression of single motoneuronal cells (columns) after hierarchical clustering using the 20 most variably expressed genes (rows) following the method of Li *et al* (2017). Hierarchical clustering was performed using ward linkage on an Euclidean distance metrics. Colour code represents gene expression levels (see Materials and Methods). Three distinct clusters with similar expression patterns are labelled in grey (cluster 1), purple (cluster 2) and green (cluster 3), these clusters correspond to clusters shown in Fig 1B.Expression of three marker genes, *elav* (pan‐neural), *VGlut* (glutamatergic MNs) and *FasII* (axon) were evaluated for each of the three clusters shown in (A). See Materials and Methods, section *Data visualization* for a definition of boxplot elements. Individual data points correspond to single cells (biological replicates), *n* = 758 (cluster 1), *n* = 76 (cluster 2), *n* = 165 (cluster 3).Bar chart shows the number of cells expressing different numbers of *Hox* genes. In sum, 749 of 999 cells express at least one *Hox* gene (~ 75% *Hox* gene coverage).
*Left panel:* Multiplex HCR visualizes the expression pattern of *VGlut*, a general marker for glutamatergic MNs, and two key marker genes, *twit* and *Vmat*, which drive the clustering shown in (A). The dashed boxes in the upper panels, which show the lateral and dorsal view of representative embryos, are displayed at higher resolution in the lower panel. *Right panel:* Venn diagram showing ratios of cells labelled with these key marker genes, which are compared with ratios expected from scRNA‐Seq experiments.

**Figure EV3:**
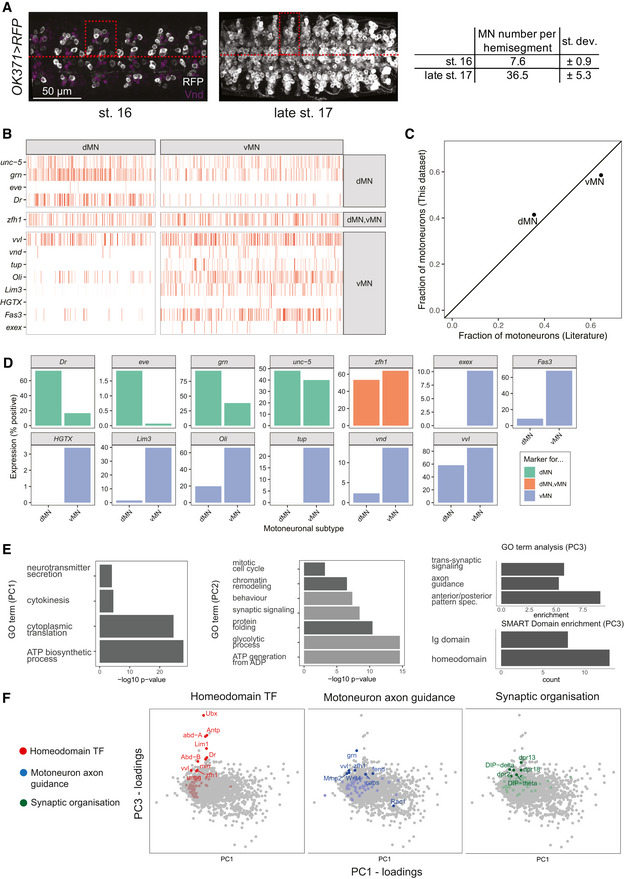
Detailed analysis of single cell data identifies known MN subtypes and variable processes in stage 16 embryonic MNs *Left panel*: ventral view of representative stage 16 and late‐stage 17 embryos expressing UAS‐*RFP* under the control of *OK371*‐GAL4. The red dashed lines highlight the midline, the dashed boxes indicate one hemisegment. Within each hemisegment, RFP‐positive cells were counted to define the average number of MNs present per abdominal hemisegment. *Right panel:* table depicting the average number of MNs per hemisegment counted in stage 16 and late‐stage 17 embryos.Expression of known motoneuron subtype markers (Landgraf *et al*, 1999; Certel & Thor, 2004; Garces & Thor, 2006; Technau *et al*, 2014; Zarin *et al*, 2014; Couton *et al*, 2015) on *n* = 758 cells from the *twit^low^* cluster. Columns correspond to single cells. Cells were assigned as dorsally projecting MNs (dMN) or ventrally projecting MNs (vMN) by computing expression scores on dMN and vMN markers. If a given marker was observed in a single cell, −log(*p*) was added to the respective score, where *p* is the total fraction of cells expressing a marker.Scatter plot comparing the fraction of MNs falling into the distinct classes according to literature (Zarin *et al*, 2014), and according to the assignment performed in (B).Bar chart depicting the expression of the marker genes in the different populations.Principal component analysis (PCA) of genes expressed in *twit^low^* cells. GO term analysis for biological processes was performed on the top 10% genes with highest loadings on principal component 1, PC1 (log 10 *P*‐value; left), principal component 2, PC2 (middle) and principal component 3, PC3 (right). GO term and SMART domain analysis was performed on the top 300 genes representing the most enriched candidates in the PCA. Dark grey indicates processes enriched among genes with positive loadings, light grey indicates processes enriched among genes with negative loadings. Together these analyses indicated that PC1 and PC2 are associated with metabolic processes, cellular differentiation and/or technical variation, while PC3 is associated with anterior/posterior patterning and synaptic processes.Principal component loadings plots highlighting homeodomain TF genes (red) as well as genes associated with the GO terms MN axon guidance (blue) and synaptic organization (green). Points with label correspond to the highest 5% of loadings.

**Figure 1:**
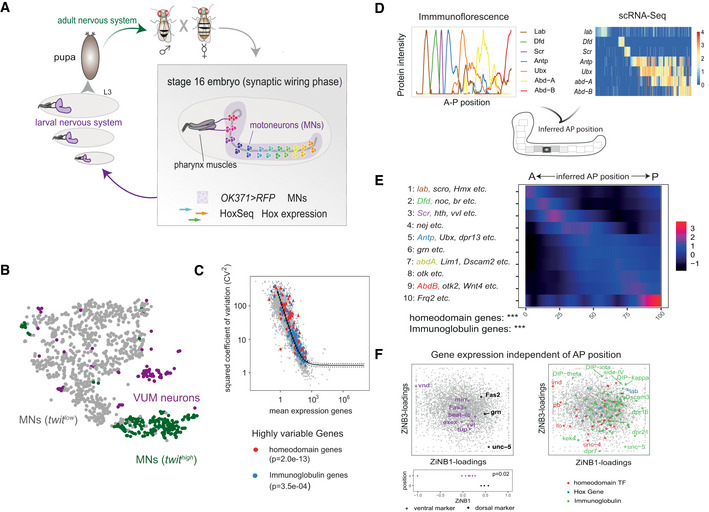
scRNA‐Seq identifies highly variable homeodomain TF expression in late embryonic MNs at the time of synaptic wiring Schematic drawing depicts the step‐wise development of the nervous system in *Drosophila*, starting during embryogenesis, progressing through three larval stages and metamorphosing during pupal stage into the adult nervous system. The first connections in the neuromuscular system are formed between MNs and muscles in late *Drosophila* embryos (stage 16). MNs at this stage expressing UAS‐*RFP* under the control of the motoneuronal driver *OK371‐*GAL4 (*OK371 > RFP*) are color‐coded along the ventral nerve cord according to different patterns of *Hox* gene expression. *OK371‐RFP‐*positive MNs were FACS sorted and single cells were sequenced by targeted Smart‐Seq2 to enrich for *Hox* gene representation (HoxSeq) as spatial markers (see also Materials and Methods).t‐distributed stochastic neighbour embedding (t‐SNE) plot of *n* = 999 single‐cell transcriptomes. Colours correspond to three clusters, VUM neurons (purple), *twit^low^* (grey) and *twit^high^* (green) MNs.Identification of highly variable genes in the *twit^low^* cluster using the method by (Brennecke *et al*, 2013). Scatter plot depicts the mean expression for each gene and squared coefficient of variation across *twit^low^* cells. The solid line indicates the fit, dashed lines the 95% confidence interval. Genes with a significantly elevated variance are shown as triangles, other genes as circles. Different gene classes are colour coded. *P*‐values shown were retrieved by a hypergeometric test for enrichment of the respective gene class among highly variable genes.Outline of strategy to map single MNs to a position along the AP axis (see also Materials and Methods). Upper panel: Intensities of Hox protein expression along the ventral nerve cord measured by immunofluorescence (upper left panel*)* and co‐expression patterns of *Hox* gene transcripts measured by scRNA‐Seq (upper right panel*)* were used as input. Upper right panel depicts a heatmap of color‐coded Hox gene expression levels; columns correspond to *n* = 758 single *twit^low^* MNs. Lower panel: AP position is inferred form scRNA‐Seq data by probabilistically mapping *Hox* gene expression pattern in each individual cell to the immunofluorescence reference data.Genes with significant variation along the AP axis were identified and clustered into 10 groups of distinct expression patterns (Materials and Methods). Heatmap shows average gene expression per cluster (rows) across single cells (columns). Asterisks indicate *P*‐value of a hypergeometric test for enrichment of protein domains, ***P < 0.001.
*Left panel:* ZINB‐WaVE (Risso *et al*, 2018, 2019) was used to statistically separate gene expression variability into parts linked to AP position and parts independent thereof. Scatter plot of ZINB‐WaVE loadings separates known dorsal and ventral marker genes on ZINB‐WaVE component 1. *Right panel:* Genes encoding homeodomain TFs and genes encoding Ig domain molecules (see colour code) show high loadings on ZINB‐WaVE components 1 and 3, demonstrating high variability independent of AP position.

For the experiment, single *RFP*‐expressing MNs were sorted from a pool of precisely staged embryos by fluorescence‐activated cell sorting (FACS) (Fig 1A). In total, 1,536 MNs were sequenced by SMART‐Seq2 (Picelli *et al*, 2014) from pooled embryos. After filtering based on a minimum of 500 genes observed with 10 reads each, 999 single‐cell transcriptomes were retained (Fig EV1D). Thus, every biologically unique motoneuronal cell (~140 *OK371*‐positive cells in a single embryo, Fig EV1A–C) was sequenced in approximately 7 biological replicates in our dataset. By comparing our dataset to recently published data, we confirmed that the quality of our data matches the standards in the field, in particular with regard to sequencing depth (Fig EV1E and F). A median of 1,202 unique genes were observed per cell, and a negligible fraction of 0.1% of reads mapped to the mitochondrial genome, supporting the high technical quality of the data. Abundant expression of motoneuronal marker genes like *Vesicular glutamate transporter (VGlut)* and *embryonic lethal abnormal vision (elav)* indicated successful sorting of the targeted cell population (Fig EV2B).

*Hox* genes are known to be expressed in a consecutive order along the AP axis of *Drosophila*. We used this property to precisely locate single‐cell transcriptomes along the AP axis as further described below. To this end, we implemented a custom modification of the SMART‐Seq2 protocol by adding primers targeting each *Hox* gene to the reverse transcription (RT) and preamplification step that permits an increased representation of the lowly expressed *Hox* genes as spatial markers (Fig 1A, see Materials and Methods) (Giustacchini *et al*, 2017; Velten *et al*, 2021). Despite the low expression of *Hox* genes in late embryonic stages, which is common to all TF encoding genes, we identified 75% of the MNs to express at least one *Hox* gene (Fig EV2C).

To explore the molecular diversity of the MNs, we performed two independent unsupervised analyses, t‐distributed neighbour embedding (tSNE) and hierarchical clustering (Figs 1B and EV2A). Both methods identified a cluster corresponding to modulator neurons (VUMs, 8% of the cells) as well as two large, yet distinct clusters of cells that differ in the expression of the marker genes *rhea* and *target of wit* (*twit*). VUM MNs belong to a very distinct MN subtype expressing a combination of subtype‐specific marker genes, *Vesicular monoamine transporter (Vmat*; Fig EV2A and D)*, Tyramine β* hydroxylase (*Tbh), diacyl glycerol kinase (dgk)* and the motoneuronal marker *Zn finger homeodomain 1* (*zfh1)* (Stagg *et al*, 2011), which we all identified in the VUM neuron cluster. These type II glutamatergic/octopaminergic MNs exhibit modulator roles in taste responses (Sink & Whitington, 1991; Landgraf *et al*, 1997; Siegler & Jia, 1999; Stagg *et al*, 2011), while the *twit^low^* and *twit^high^* cluster can be assigned to the abundant glutamatergic type I MN class (Hoang & Chiba, 2001; Kim *et al*, 2009). *In situ* hybridization chain reactions (HCR) of late stage embryos localized *twit* transcripts in median and lateral clusters of posteriorly located MNs (Fig EV2D) (Kim & Marqués, 2012), suggesting that those two groups of MNs, the VUMs and *twit^high^* MNs, represent indeed two distinct motoneuronal subtypes with different locations.

Taken together, these analyses showed that the dataset generated in this study was of high quality, consistent with published data and provided an approximately 7‐fold cellular coverage of each biologically unique MN at the synaptic wiring stage. In addition, we identified three rather homogenous cluster that can be identified by the expression of *twit^high^*, *twit^low^* and *Vmat*, corresponding to type I and type II MNs.

### The expression of homeodomain transcription factor and Ig domain genes are highly variable within *twit^low^* MNs

To investigate processes required for MN diversity during the synaptic wiring phase, we focused on the largest cluster of MNs expressing low levels of *twit* (*twit^low^*). This cluster contains the majority of glutamatergic type I MNs that are equally distributed along the ventral nerve cord, rather than specialized subtypes of MNs such as the VUMs or *twit^high^* clusters that are unevenly distributed along this embryonic body axis (Fig EV2D). Although this population in the largest cluster (*twit^low^*) appears rather homogenous, our dataset confirms mutual exclusive expression of known markers for the two major motoneuronal subsets, the dorsally and ventrally projecting MNs (Landgraf *et al*, 1999; Certel & Thor, 2004; Garces & Thor, 2006; Landgraf & Thor, 2006; Technau *et al*, 2014; Zarin *et al*, 2014; Urbach *et al*, 2016; Zarin & Labrador, 2019), while common markers are identified in both subsets including *unc5* (Keleman & Dickson, 2001) (Fig EV3B and D). Importantly, the MN subtypes are represented in our dataset in the ratios expected from literature (Landgraf *et al*, 1999; Garces & Thor, 2006; Zarin *et al*, 2019) (Fig EV3C). Hence, these data suggest that the population profiled here is very similar and representative of MNs present at the end of embryogenesis (stage 17).

Using a statistical test to discover biologically variable genes from scRNA‐Seq data (Brennecke *et al*, 2013), we observed that homeodomain TF and Ig domain encoding genes displayed a very high biological variability within *twit^low^* cells (Fig 1C). Similar results were obtained by principal component analysis (PCA) of the *twit^low^* cells, which demonstrated that the expression of homeodomain TFs and CSPs involved in synaptic matching and axon guidance co‐varied (Fig EV3E and F). In particular, the expression of known mediators of synaptic specificity, for instance Dpr protein‐encoding genes (Özkan *et al*, 2013; Carrillo *et al*, 2015), was highly variable within the *twit^low^* cluster and co‐varied with homeodomain TF gene expression (Fig EV3F).

Taken together, more detailed analysis on the largest type I MN cluster (*twit^low^*) revealed that within this cluster all major motoneuronal subtypes were detected and that homeodomain TF and CSP expression was highly variable and co‐varied.

### Homeodomain TF combinations are associated with spatial variability

**Figure EV4:**
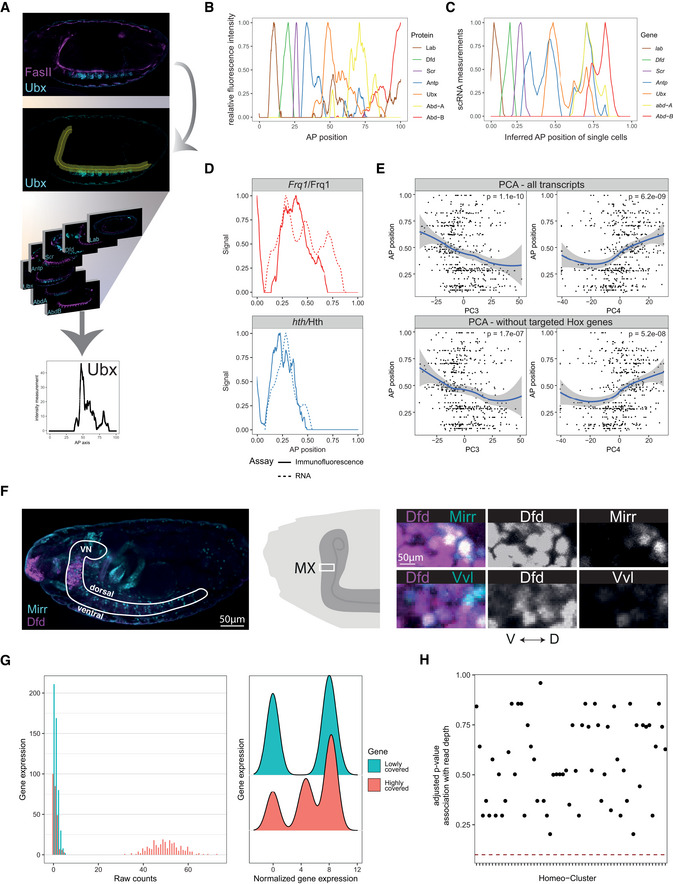
The AP axis can be spatially reconstructed based on immunofluorescence measurements of Hox proteins Pipeline for protein intensity measurements of the seven Hox TFs (Lab, Dfd, Scr, Antp, Ubx, AbdA and AbdB) expressed along the AP axis of the ventral nerve cord in a consecutive order. *Upper panel:* The motoneuronal marker FasII (magenta) was used as reference to measure Hox TF expression patterns in different embryos in a standardized manner (see Materials and Methods). *Lower panel:* Procedure of translating different fluorescence intensity measurements into standardized graphs by Fiji.Normalized and smoothened protein measurements of Hox TFs (colour code) are combined in one graph (see Materials and Methods). *X*‐axis: relative AP position in % (normalized to length of ventral nerve cord), *Y*‐axis = relative fluorescence intensity measurement (normalized to max. intensity).Normalized and smoothened single‐cell mRNA measurements of *Hox* gene expression (colour code) arranged along the inferred AP position (See Fig 1D, Materials and Methods).Proof of concept: comparison of expression of two candidate genes (*frq1* and *hth*) on the protein level (solid line) along the measured AP axis and the mRNA expression level (dashed line) along the inferred AP position (see Fig 1D, Materials and Methods), highlighting a high degree of agreement.Scatter plots relating principal component scores on PC3 and PC4 to AP position. PCA was performed using only cells from the *twit^low^* cluster. PCA was performed including (upper panel) or excluding (lower panel) *Hox* genes to rule out any qualitative biases created by targeted *HoxSeq*. *P*‐values were computed based on the hypothesis that the true correlation is different from 0 using a fisher transform of correlation coefficients.
*Left panel:* The homeodomain TFs Vvl and Mirr identified in ZiNB‐WAVE analysis were investigated for the localization along the DV axis of the ventral nerve cord (highlighted in white) of an early‐stage 17 *Drosophila* embryo. Here, co‐expression of Dfd (magenta) and Mirr (blue) is shown. *Central panel:* Illustration highlighting the maxillary segment (Mx). *Right panel:* Zoom on the expression of Vvl and Mirr in the Dfd expressing maxillary segment (Mx; magenta) is shown, highlighting that Vvl and Mirr are expressed in ventral regions.Normalization strategy of scRNA‐Seq data analysis modified for sparse and lowly expressed genes (see Materials and Methods). For gene “Low”, expression takes 0 or small values from 1 to 10 counts, for gene “High”, expression can be 0, low (1–10), or high (30‐100). After normalization, quantitative differences between cells expressing gene “Low” are effectively voided, whereas they are preserved for gene “High”. *Left panel:* Histogram of raw data, *right panel*: Density plot after normalization.Scatter plot depicting for each homeo‐cluster the strength of association with a technical covariate (sequencing depth). *P*‐values were calculated using the Wilcoxon test contrasting sequencing depth in cells from that cluster, and all other cells. The dotted read line indicates the *P*‐value required for significance (0.05). All associations are therefore not significant.

We hypothesized that the high variability of homeodomain TF gene expression within otherwise homogenous MNs is caused by spatial cues. To investigate this possibility, we spatially mapped single MNs along the AP body axis using *Hox* gene expression as spatial reference points. We first created a high‐resolution map of Hox protein expression by immunofluorescence, showing that anterior Hox proteins were expressed in clearly defined stripes, whereas expression of posterior Hox proteins was partially overlapping (Figs 1D, and EV4A and B). The same co‐expression patterns were observed in our scRNA‐Seq data (Figs 1D and EV4C), allowing us to probabilistically map cells from the scRNA‐Seq dataset to a position along the AP axis (Fig 1D, see Materials and Methods). We validated this mapping strategy by immunofluorescence. To this end, we used the inferred AP position to estimate the expression pattern of every gene along the AP axis (Fig 1E). We thereby identified candidate genes with differential expression along this axis. Importantly, these candidates were not used for constructing the model. For two such candidates, *Frq1* and *hth*, we compared the predicted expression pattern to immunofluorescence data and observed a high agreement (Fig EV4D). Inferred AP position was significantly correlated with principal components 3 and 4 (Fig EV4E), indicating that AP position profoundly affects the entire transcriptome of each cell.

Interestingly, the above‐described heterogeneity of homeodomain encoding genes is aligned with the AP position, but we additionally found more variability of homeodomain encoding genes independent of AP position. To that end, we made use of ZINB‐WaVE analysis (Risso *et al*, 2018, 2019). We separated scRNA‐Seq data into variability linked to the known covariates (AP position and technical variability) and into processes statistically independent thereof (Fig 1F). On the first component of AP‐independent variability, we identified one group of genes known as marker for dorsal–ventral (DV) position (Bhat, 1999; Skeath, 1999; Urbach *et al*, 2006). These genes were ordered according to their localization in the embryo from dorsal to ventral. Again, homeodomain TFs and Ig surface proteins were among the most variable genes on this AP‐independent axis of variability. Immunofluorescence experiments of two ventral marker genes, *mirror (mirr)* and *ventral veins lacking (vvl),* confirmed the predicted DV position (Fig EV4F).

Together, these analyses showed that highly variable homeodomain encoding genes are associated with spatial heterogeneity, which is in line with previous findings (Bhat, 1999; Skeath, 1999; Urbach *et al*, 2006). Furthermore, using *Hox* gene expression as spatial markers allowed us to map individual MNs along the AP axis.

### Cell‐specific homeodomain TF combinations delineate *Drosophila* embryonic MN heterogeneity during synaptic wiring

**Figure 2:**
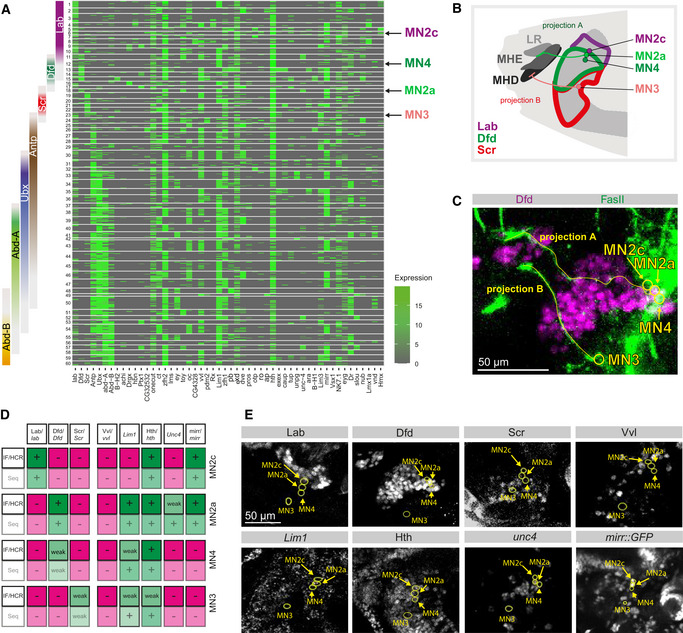
Specific combinations of homeodomain TFs are expressed in embryonic MNs during synaptic wiring Heatmap depicting the expression of homeodomain encoding genes (columns) across *n* = 537 single *twit^low^* MNs for which spatial mapping information was available (rows). Rows and columns are arranged by hierarchical clustering. Normalized expression levels are color‐coded. Arrows indicate selected clusters for follow‐up studies (see Fig 1D). *Hox* gene expression in individual cells as identified by scRNA‐Seq is shown on the left by coloured bars, clusters 1 to 60 expressing specific combinations of homeodomain genes are shown on the left.Schematic drawing of a stage 17 *Drosophila* embryonic head, highlighting the position of selected MNs (MN2c, MN2a, MN4, MN3) and feeding muscles (LR, MHE, MHD) located in consecutive segments expressing different Hox proteins (Lab, Dfd, Scr). Each MN innervates a specific muscle by a FasII‐positive axonal projection, projection A connects MN2a and the MHE muscle, while projection B connects the MN3 with the MHD muscle.Visualization of the different MNs based on Dfd expression in MN2a and the MHE, which is innervated by a FasII‐positive axon emerging from MN2a (projection A), while the motoneuronal projections from MN3 (projection B) innervates the Dfd‐negative MHD located just underneath the MHE (see also B). All MNs are highlighted by yellow circles.Chart comparing the expression of eight homeodomain TFs in the four anterior MNs (MN2c, MN2a, MN4, MN3) as identified by scRNA‐Seq (Seq) and immunohistochemistry (IF/HCR), with the absence of expression color‐coded in magenta and the presence of expression in green. The genes were selected for validation, as they allowed an unambiguous distinction of the four anterior MNs according to the scRNA‐Seq data.Validation of the specific expression of the eight homeodomain TFs (Lab, Dfd, Scr, VVl, Lim1, Hth, Unc4 and Mirr) in MN2c, MN2a, MN4 and MN3 by immunohistochemistry or *in situ* HCR. For expression analysis, proteins or transcripts were detected with specific antibodies (Lab, Dfd, Scr, Hth, Vvl) or HCR probes (Lim1, unc4) using wild‐type embryos. Mirr expression was detected by GFP antibody stainings using a GFP fusion line (*mirr::GFP*). The stereotypic position of MN2c, MN4, MN2a and MN3 was identified by the two axon projections A and B (labelled by FasII), which invariantly innervate the MHE (projection A) and MHD (projection B) muscles in wild‐type embryos. MN2c and MN4 are the two MNs adjacent to MN2a. The four MNs are highlighted by yellow circles.

Our analyses suggested that homeodomain encoding genes, unlike other groups of genes, can be used to more precisely depict the cellular heterogeneity of *twit^low^* MNs. Thus, we performed unsupervised hierarchical clustering using only the highly variable homeobox genes as input. In addition, we used a normalization strategy that effectively classifies lowly expressed genes as “expressed” or “not expressed”, whereas the expression of genes with sufficient sequencing coverage was represented in a more quantitative manner (Fig EV4G, see Materials and Methods). Furthermore, cells not expressing any *Hox* genes were excluded from further analyses, as they might include few glutamatergic brain neurons potentially targeted by *OK371*‐GAL4 (Fig EV1A). This approach identified small groups of cells expressing a unique combination of homeodomain‐containing genes (Fig 2A). Using this strategy, we could show that these patterns were independent of technical covariates and that clustering is not affected by technical noise common to single‐cell transcriptomic data (Fig EV4H).

**Figure EV5:**
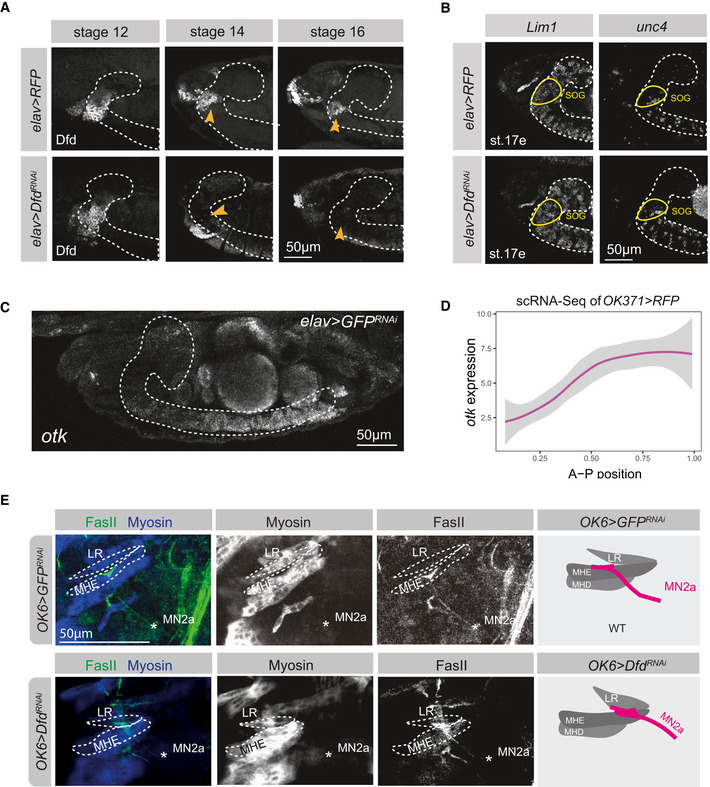
Homeodomain TFs control Immunoglobulin gene expression in the embryonic nervous system Representative confocal images of stage 12, 14 and 16 *Drosophila* control (*elav > RFP*) and *elav > Dfd^RNAi^* embryos, depicting Dfd expression (red) and the ventral nerve cord, which is outlined by a white dashed line in all embryos (identified by Elav staining). The orange arrowheads highlight the area of neuronal Dfd expression, which is abolished in *elav > Dfd^RNAi^* embryos at stage 14.HCR for identifying *Lim1* and *unc4* transcripts in *Drosophila* stage 17 embryos. RNA expression of both genes is analysed in control (*elav > RFP*) versus Dfd‐depleted (*elav > Dfd^RNAi^*) animals, the ventral nerve cord is highlighted by a white dashed line, the Dfd‐expressing subesophageal ganglion (SOG) is indicated by a yellow circle.Representative confocal image of a stage 17 *Drosophila* embryo, highlighting expression of Ig gene *otk* along the ventral nerve cord (white dashed line) as detected by HCR.Graph depicting *otk* mRNA expression level along the inferred AP position based on the scRNA‐Seq data.Representative confocal image of a stage 17 embryonic head with Myosin‐expressing muscles (blue) and FasII‐expressing axonal projections (green) in animals after RNA interference with *Dfd* in MNs by means of the *OK6*‐GAL4 driver. A zoom on the projection (FasII staining) of MN2a to the MHE and LR muscles of an early‐stage 17 *Drosophila* embryo are shown. Asterisks highlight the location of MN2a, which is identified by the FasII‐expressing axonal projection emerging from a Dfd‐expressing MN, which normally innervates the Dfd‐expressing MHE muscle. The panel on the right side represents a schematic drawing of the confocal image shown on the left side, summarizing the innervation of the anterior muscles (LR, MHE, MHD) by projections emerging from MN2a (magenta) in the perturbation condition.

The identification of defined groups of cells with homogenous expression of homeodomain TFs required a splitting of the dataset into a specific number of clusters. Since a statistical specification of cluster numbers from scRNA‐Seq data remain an unresolved issue in the field (Zhu *et al*, 2018; Luecken & Theis, 2019), we used our knowledge that embryos at the exact time point of sequencing harbour around 140 *OK371*‐positive MNs differentially distributed along the AP axis (Fig EV1, EV2, EV3, EV4, EV5). Due to the bilateral symmetry, this corresponds to 70 biologically unique cellular identities, including the *twit^high^*, *twit^low^* and VUM cells. We then arbitrarily clustered the data into 60 groups, corresponding to the estimated number of *twit^low^* cells (Fig EV2D).

**Figure 3:**
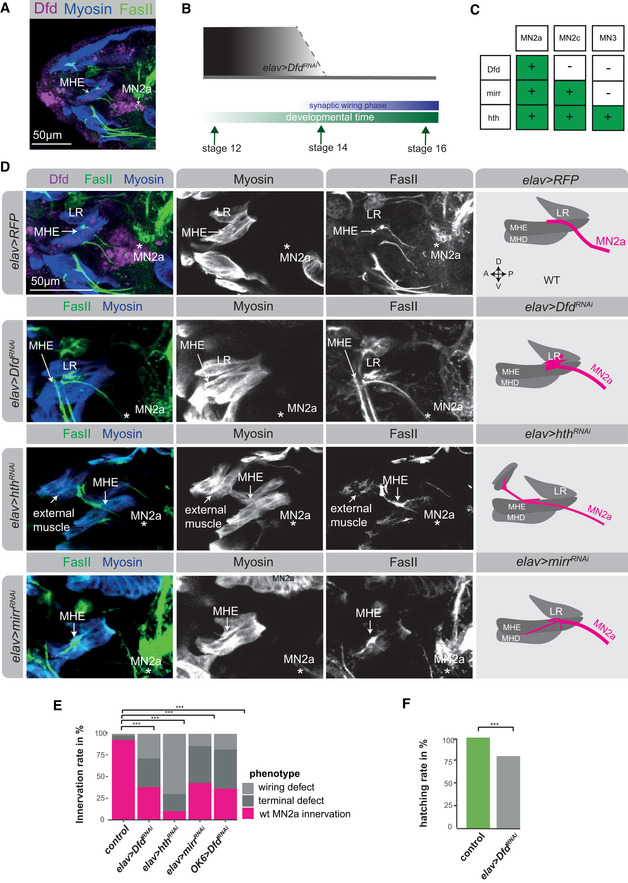
Homeodomain TFs expressed in MNs fine tune wiring specificity in the neuromuscular system Representative confocal image of a stage 17 embryonic head with Dfd‐expressing cells (purple), Myosin expressing muscles (blue) and FasII‐expressing axonal projections (green) highlighted. The MN2a is identified as a Dfd‐expressing neuron projecting a FasII‐expressing axon to the Dfd‐positive MHE muscle. This stereotypic innervation is the basis for identifying wiring defects after RNAi interference shown in (D).Time course of RNAi induced effect using the pan‐neural *elav‐GAL4* driver line, showing that efficient knock‐down of gene expression is achieved only at embryonic stage 14.Chart highlighting the expression of the three genes used for RNA interference, *Dfd*, *hth* and *mirr*, in MN2c, MN2a and MN3.Representative confocal images of stage 17 embryonic heads highlighting expression of Dfd (purple), Myosin in muscles (blue) and FasII in axonal projections (green) in control (*elav > RFP*) animals and after RNA interference with three different homeodomain TF genes, *Dfd*, *hth* and *mirr*, which are all co‐expressed in MN2a (see Fig 2E). A zoom on the projection (FasII staining) of MN2a to the MHE and MHD muscle of an early stage 17 *Drosophila* embryo are shown. Asterisks highlight the location of MN2a, which is in all cases identified by the FasII expressing axonal projection emerging from a Dfd expressing MN, which normally innervates the Dfd‐expressing MHE muscle (as shown in the *elav > RFP* control). The panel on the right side represents a schematic drawing of the confocal image shown on the left side, summarizing the innervation of the anterior muscles (LR, MHE, MHD) by projections emerging from MN2a (magenta) in control and perturbation conditions.Quantification of different phenotypes manifesting after RNA interference in comparison to control animals: MN2a axons projecting to the MHE muscle, representing the wild‐type (wt) innervation pattern (magenta); MN2a axons projecting to muscles other than the MHE termed abnormal innervation (light grey); abnormal synaptic morphologies at MN2a‐derived axon terminals termed terminal defects (dark grey). Note, each genetic experiment was performed in parallel to an adequate control experiment using the same driver line crossed to a line that controls expression of either UAS*‐RFP* or UAS‐*GFP^RNAi^*. Each experiment was performed in triplicates, innervation rates were calculated from *n* = 56 for *elav > RFP*, *n* = 21 for *elav > Dfd^RNAi^*, *n* = 10 for *elav > hth^RNAi^* and *n* = 7 for *elav > mirr^RNAi^*. In the case of Dfd knock‐down, two different driver lines were used, the pan‐neural *elav‐*GAL4 and the motoneuron‐specific *OK6‐*GAL4 drivers, respectively. Both result in similar phenotypes, highlighting that the *elav‐*GAL4‐driven effects are specific to MNs. *P*‐values between two genetic conditions were calculated by a two‐sided Fisher test. ***P < 0.005.Correct MHE innervation is required for hatching of *Drosophila* embryos from the eggshell (Friedrich *et al*, 2016). Hatching rate was calculated based on the number of L1 larvae observed after 24 h in genetic crosses, depleted of Dfd (UAS‐*Dfd^RNAi^*) in neurons (*elav‐*GAL4; *n* = 217) compared to crosses with control animals (mock = *elav > RFP*; *n* = 156). *P*‐values were calculated by a two‐sided Fisher test, ***P < 0.005.

We next validated that these groups correspond to defined cells in a few cases by using the stereotypic position of axon projections as reference for defined cells. As previously described (Friedrich *et al*, 2016), we could reproducibly identify the MN innervating the mouth hook elevator muscle (MHE) by its Dfd expression and FasII staining (Figs 2B and C, and 3A). Based on its similar position in *Calliphora vicina* (Schoofs *et al*, 2010a, 2010b), we termed this axon projection MN2a. The neurons directly adjacent to MN2a were termed MN2c and MN4, while the MN innervating the mouth hook depressor muscle (MHD) was called MN3 (Schoofs *et al*, 2010a, 2010b). MN2a, MN2c, MN3 and MN4 were identified based on their position with regard to the FasII‐stained axon projections. We then separately measured protein or RNA expression of Lab, Dfd, Scr, Vvl, *Lim1*, Hth, *unc‐4* and *mirr* in these MNs using immunofluorescence and hybridization chain reaction (HCR; Fig 2D and E). Based on the expression pattern of these eight genes, we were able to assign all four MNs unambiguously to clusters defined by the transcriptome data (Dataset EV1; MN2c = cluster C6, MN2a = cluster C18, MN4 = cluster C12, MN3 = cluster C20).

While difficulties in microscopically identifying all individual 60 MNs impeded us from unanimously designating all clusters as biologically unique cells, our analyses demonstrated that a homeodomain TF combination is associated with cellular heterogeneity during the synaptic wiring phase and is specific to small groups of cells or even single cells.

### Homeodomain TFs modulate synaptic target specificity of two distinct MN projections

After having identified cell‐specific combinations of homeodomain TFs associated with motoneuronal heterogeneity, we wanted to test their contribution to synaptic specificity in individual embryonic MNs. To this end, we focused our analysis on MN2a, which co‐expresses the homeodomain TFs Dfd, Mirr and Hth, and its projection to the MHE muscle (Fig 3A). We used the pan‐neural driver *elav‐*GAL4 (Luo *et al*, 1994) to interfere with these three TF genes by RNAi‐mediated gene silencing (RNAi) and examined synaptic defects of MN2a in stage 17 *Drosophila* embryos. The *elav‐*GAL4 driver is ideally suited for temporal interference with proteins during the late synaptic wiring phase, as RNAi‐mediated knockdown is realized earliest in stage 14 of motoneuronal axonogenesis (Figs 3B and EV5A), thereby leaving the initial specification of these cells unaffected. To complement this approach, we used additionally a more specific driver for MNs, *OK6‐*GAL4, which is active in glutamatergic MNs only (Aberle *et al*, 2002).

Defects on MN2a induced by RNAi were classified into two distinct categories, wiring defects (i.e. mistargeting of neurons to the wrong muscle) and terminal defects (i.e. abnormal synaptic morphologies at axon terminal sites; Fig 3C–E). Terminal defects were quantified but not analysed in detail (Fig 3E). Our experiments showed late neural interference with *Dfd*, *mirr* and *hth* resulted in an abnormal muscle targeting of MN2a (Fig 3D and E); however, in each case, MN2a innervated different ectopic target sites. For example, late neural depletion of *Dfd* led to mistargeting of the MN2a to the labial retractor muscle (LR), a muscle located directly anterior to the MHE muscle, in 29% of the embryos, while interference with *hth* resulted in a mis‐guidance of the MN2a to external muscles in 70% of the embryos (Fig 3D and E). In the case of *mirr* knockdown, the MN2a tended to target the MHD muscle located posterior to the MHE; however, wiring defects were less pronounced and frequent (Fig 3D and E). Importantly, defects observed in *elav > Dfd^RNAi^* and *OK6 > Dfd^RNAi^* embryos were comparable (Figs 3D and 3E, and EV5E), showing that the phenotypes were primarily due to knockdown of gene expression in MNs and not or to a minor extent caused by non‐autonomous effects from non‐MNs labelled by *elav*‐GAL4. In the case of Dfd depletion, we additionally investigated behavioural defects of mouth hook movements by hatching rate assays and measured decreased rates (Fig 3F). Neural interference with one homeodomain TF did not affect the expression of other homeodomain TF genes, which we exemplarily showed for *Lim1* or *unc4* in *Dfd*‐depleted embryos (Fig EV5B). Together, these results showed that loss of specific homeodomain TFs, which are combinatorially expressed in single MNs, leads to cell‐specific synaptic targeting defects and changes in target preferences of an individual MN at the end of embryogenesis.

**Figure 4:**
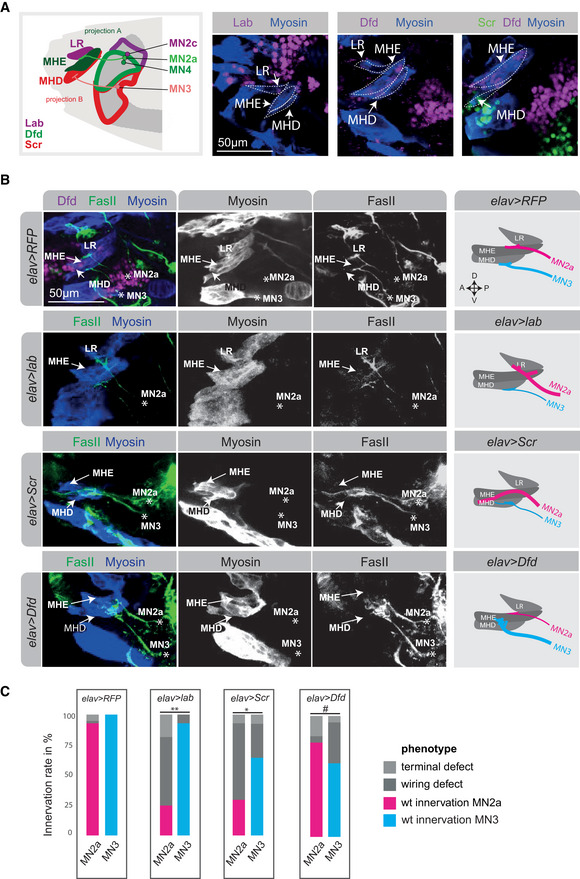
Ectopic homeodomain TF expression changes muscle target choice in a predictive manner *Left panel:* Schematic drawing of a stage 17 *Drosophila* embryonic head, highlighting the stereotypic innervation of the anterior feeding muscles by specific MNs, MHE (green) by MN2a, which only expresses Dfd, MHD (red) by MN3, which only expresses Scr, and LR (purple) by MN2c, which only expresses Lab. *Right panel:* Representative confocal images of early stage 17 *Drosophila* embryonic heads, highlighting the expression pattern of the anterior Hox TFs Lab, Dfd and Scr, showing that Lab is expressed in the LR muscle, Dfd in the MHE muscle and Scr in the MHD muscle. The muscles are indicated by white dashed lines.Representative confocal images of stage 17 embryonic heads highlighting the expression of Dfd (purple), Myosin in muscles (blue) and FasII in axonal projections (green) in control (*elav > RFP*) animals and animals mis‐expressing the Hox TFs Lab, Dfd and Scr by means of the *elav‐*GAL4 driver. A zoom on the projections (FasII staining) of MN2a and MN3 to the MHE and MHD muscles of an early‐stage 17 *Drosophila* embryo are shown. Asterisks highlight the location of MN2a and MN3, respectively. MN2a is identified by the FasII‐expressing axonal projection emerging from a Dfd‐expressing MN, which normally innervates the Dfd‐expressing MHE muscle (as shown in the *elav > RFP* control), while MN3 is identified as the MN underneath MN2a, which normally innervates the MHD muscle (as shown in the *elav > RFP* control). The panel on the right side represents a schematic drawing of the confocal image shown on the left side, summarizing the innervation of the anterior muscles (LR, MHE, MHD) by projections emerging from MN2a (magenta) or MN3 (cyan) in control and mis‐expression conditions.Quantification of different phenotypes manifesting in MN2a and MN3 after Hox mis‐expression in comparison to control animals: MN2a axons projecting to the MHE muscle, representing the wild‐type (wt) innervation pattern of MN2a (magenta); MN3 axons projecting to the MHD muscle, representing the wild‐type (wt) innervation pattern of MN3 (cyan); MN2a or MN3 axons projecting to muscles other than the MHE or MHD termed wiring defects (dark grey); abnormal synaptic morphologies at MN2a‐ or MN3‐derived axon terminals termed terminal defects (light grey). Note, each genetic experiment was performed in parallel to an adequate control experiment using the same driver line crossed to a line that controls expression of either UAS*‐RFP* or UAS*‐GFP^RNAi^*. Each experiment was performed in triplicates, innervation rates were calculated from *n* = 16 for *elav > lab*, *n* = 18 for *elav > Dfd* and *n* = 25 for *elav > Scr*. *P*‐values between two genetic conditions were calculated by a two‐sided Fisher test. ^#^ *P* < 0.1, *P < 0.05, **P < 0.01.

The hypothesis that combinations of homeodomain TFs drive synaptic wiring and specificity implies that not only the loss of factors but also their ectopic activity should cause wiring defects. To test this assumption, we induced the expression of the homeodomain‐containing *Hox* genes *lab*, *Dfd* and *Scr*, which are active in consecutive segments in the *Drosophila* head (Fig 4A), in all neurons using the *elav‐*GAL4 driver and investigated the innervation patterns of MN2a and MN3 (Fig 4A). In unperturbed conditions, the Dfd‐expressing MN2a normally innervates the MHE muscle (Figs 2C and 3A *)*, which also expresses Dfd (Fig 4A), while the Scr‐expressing MN3 targets the Scr‐positive MHD muscle (Figs 2C and 4A). Ectopic expression of *Dfd* caused the MN3 to innervate the more anteriorly located Dfd‐expressing MHE muscle, while ectopic *Scr* expression mis‐guided the MN2a to the more posteriorly located Scr‐positive MHD muscle (Figs 4B and C). Along the same lines, ectopic *lab* expression resulted in a high frequency of the MN2a to innervate the anteriorly located LR muscle (Fig 4B and C), which expresses Lab at low levels (Fig 4A). Intriguingly, this latter effect is reminiscent to the mistargeting phenotype observed in *Dfd* knockdown conditions (Fig 3D). These results are striking, as they show that the target preference of MNs can be changed along the AP axis in a predictive manner: mis‐expression of more anterior *Hox* genes guided MNs to innervate more anterior muscles, while more posterior *Hox* genes directed the MNs to more posterior muscles (Fig 4B and C). However, these mistargeting effects were restricted, as motoneuronal axons hardly passed across muscles to innervate targets that are even further anteriorly or posteriorly located. For example, while ectopic *lab* expression allowed the MN2a to target the LR muscle (Fig 4B), the MN3 rarely innervated this muscle by crossing the Dfd‐positive MHE muscle.

In sum, stage‐specific genetic perturbation with homeodomain TFs resulted in cell‐specific changes of muscle target selection by MNs, supporting the idea that these factors control the specificity of MN‐muscle interactions in late embryonic stages when the first neuromuscular synaptic connections are established.

### Functional relevance of Ig genes in mediating synaptic specificity

**Figure 5:**
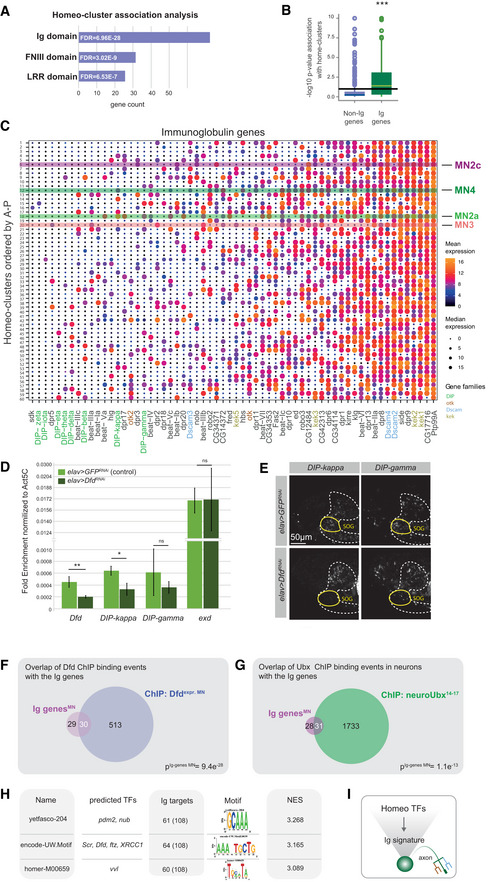
Homeodomain TFs regulate Ig genes expressed in embryonic MNs A, BAnalysis of gene classes associated with the homeodomain TF clusters. For each gene in the dataset, normalized, scaled expression in single cells was modelled as a function of homeodomain TF cluster identity, and significance of the association was determined using an *F*‐test. Panel (A) depicts the number of genes with significant homeodomain TF cluster association falling into distinct gene classes. *P*‐values were retrieved by a Fisher test. Panel (B) contrasts the *P*‐values for homeodomain TF cluster association between Ig domain genes and other genes using a boxplot. See Materials and Methods, section *Data visualization* for a definition of boxplot elements. *P* values are from a two‐sided Wilcoxon test. ***P < 0.005.CSingle cells were grouped into 60 clusters according to their expression of genes encoding homeodomain proteins (Fig 2A) and arranged along the inferred AP position (Fig 1D, 1E). For each cluster, the mean expression of Ig encoding genes was computed. Heatmap depicts Ig encoding genes in columns and clusters in rows; mean expression is color‐coded and median expression level is visualized by circle size. Gene families are highlighted in colour codes. The coloured bars highlight the Ig genes expressed in anterior MNs (MN2c, MN2a, MN4, MN3).DQuantification of Ig gene expression changes after interference with *Dfd* expression in neural cells (*elav > Dfd^RNAi^*) by qPCR. Two genes, *DIPgamma* and *DIPkappa*, were selected as potential Dfd targets based on their co‐expression in Dfd‐positive MNs. The bar chart shows the fold enrichment after normalization against Act5C of the *Dfd* and *Ig* genes in control versus *elav > Dfd^RNAi^* conditions from three biological replicates and shows that *Dfd* and *DIP‐kappa* expression are significantly reduced in *elav > Dfd^RNAi^* embryos, whereas the expression of the homeodomain TF gene *exd* is unchanged (internal control). *P*‐values were calculated from a *t*‐test: *<0.05, **<0.01, ns non‐significant. Error bar denotes standard deviation.EHCR for *DIPgamma* and *DIPkappa* transcripts in *Drosophila* stage 17 embryos. RNA expression of both genes is analysed in control (*elav > GFP^RNAi^*) versus Dfd‐depleted (elav > *Dfd^RNAi^*) animals, the ventral nerve cord is highlighted by a white dashed line, the Dfd‐expressing suboesophageal ganglion (SOG) is indicated by a yellow circle.FVenn diagram displaying an overlap of Ig domain genes expressed in MNs and genes that are bound by Dfd based on the Dfd ChIP‐Seq performed in a whole embryo (Sorge *et al*, 2012). *P*‐value was calculated using a hypergeometric test using as a reference all protein coding genes *n* = 13,920.GVenn diagram displaying the overlap of Ig domain genes expressed in MNs that are bound by Ubx identified in a neuronal tissue‐specific ChIP‐Seq (Domsch *et al*, 2019). *P*‐value was calculated as described in (F).HiRegulon analysis was used to identify TF motifs enriched in the vicinity of Ig encoding genes. Three of the top 15 highest ranked motifs of TFs are shown; the predicted targets of these motifs are homeodomain TFs.ISchematic drawing of the relationship between the homeodomain and the Ig molecule clusters in a single neuron.

Our single‐cell analyses and functional follow‐ups revealed a critical role for combinations of homeodomain TFs in controlling synaptic specificity in the neuromuscular system, motivating an investigation of potential downstream effectors. Unsupervised analysis of gene classes associated with the cell‐specific homeodomain TF expression revealed that Ig encoding genes most strongly correlated with homeodomain clusters (Fig 5A and B). To visualize this relationship, we used each of the 60 homeodomain TF clusters identified before (Fig 2A) to calculate the corresponding expression of all Ig genes within each cluster (Fig 5C, Dataset EV1). Strikingly, this analysis revealed that each MN cluster expresses a complex but unique combination of Ig genes, with some Ig genes being expressed in all MNs, some in only a subset and a few exclusively in single MNs. For example, the Ig encoding gene *off‐track (otk)* is expressed in posterior homeodomain clusters in RNA‐Seq (Figs 5C and EV5D), which we confirmed by *in situ* HCR (Fig EV5C). Some other Ig encoding genes, such as those encoding Down syndrome cell adhesion molecules (Dscams) or Kekkon (Kek*)* neurotrophin receptors (Ulian‐Benitez *et al*, 2017), are expressed in most homeodomain clusters, but are occasionally switched off in a few clusters. In other cases, for example genes of the DIP family, the Ig gene is specifically expressed in one or few homeodomain clusters (like *DIP‐iota* in cluster 28; Fig 5A). This analysis also revealed that the four anterior MNs, MN2c, MN2a, MN4 and MN3, express specific combinations of Ig genes (Fig 5C).

To corroborate the regulation of Igs by homeodomain TFs, we manipulated the expression of the homeodomain encoding gene *Dfd* in neuronal cells and investigated the effects on Ig gene expression by qPCR or HCR. According to our scRNA‐Seq data, *Dfd* is co‐expressed with the Ig genes *DIP‐gamma* and *DIP‐kappa* in MN2a (Fig 5C, Dataset EV1). Both genes were downregulated when *Dfd* expression was reduced by RNAi in neural cells using the *elav‐*GAL4 driver (Fig 5D and E). To provide further evidence that homeodomain TFs regulate Ig domain expression, we analysed previously generated whole‐embryo Dfd ChIP‐Seq data (Sorge *et al*, 2012) and Ubx ChIP‐Seq data, which were retrieved from neuronal cells of late‐stage embryos (Domsch *et al*, 2019). We found that Ig encoding genes expressed in MNs overlap with the Dfd or Ubx bound targets (Fig 5F and G). In addition, homeodomain TF binding motifs were overrepresented within regulatory sequences associated with Ig encoding genes (Fig 5H), strongly supporting a direct regulation of Ig expression by homeodomain TFs (Fig 5I).

**Figure 6:**
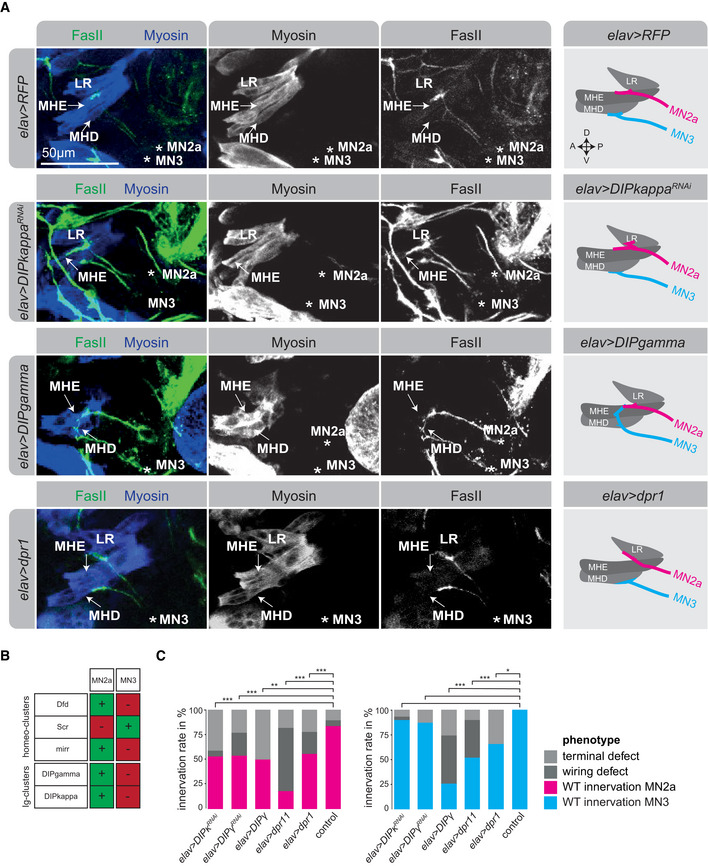
Ig genes affect synaptic wiring specificity in accordance with the motoneuronal homeodomain cluster map Representative confocal images of stage 17 embryonic heads highlighting the expression of Myosin in muscles (blue) and FasII in axonal projections (green) in control (*elav > RFP*) animals and in animals with reduced neural *DIPkappa* (*elav > DIPkappa^RNAi^*) as well as in animals, which mis‐express *DIPgamma* or *dpr1* in neural cells (*elav > DIPgamma, elav > dpr1*). Single channels focusing on the projections (FasII staining) of MN2a and/or MN3 to the MHE, MHD or LR muscles of an early‐stage 17 *Drosophila* embryo are shown. Asterisks highlight the location of MN2a and MN3, respectively. MN2a is identified by the FasII expressing axonal projection emerging from a Dfd expressing MN, which normally innervates the Dfd‐expressing MHE muscle (as shown in the *elav > RFP* control), while MN3 is identified as the MN underneath MN2a, which normally innervates the MHD muscle (as shown in the *elav > RFP* control). The panel on the right represents a schematic drawing of the confocal images shown on the left side, summarizing the innervation of the anterior muscles (LR, MHE, MHD) by projections emerging from MN2a (magenta) or MN3 (cyan) in control and mis‐expression conditions.Chart highlighting the expression of the homeodomain cluster genes *Dfd*, *Scr* and *mirr* as well as the Ig genes *DIPgamma* and *DIPkappa* in MN2a and MN3, showing that both Ig genes are only expressed in MN2a and not in MN3.Quantification of different phenotypes manifesting in MN2a and MN3 after neural RNA interference or mis‐expression of Ig genes (*DIPgamma*, *DIPkappa*, *dpr11*, *dpr1*) in comparison to control animals: MN2a axons projecting to the MHE muscle, representing the wild‐type (wt) innervation pattern of MN2a (magenta); MN3 axons projecting to the MHD muscle, representing the wild‐type (wt) innervation pattern of MN3 (cyan); MN2a or MN3 axons projecting to muscles other than the MHE or MHD termed wiring defects (dark grey); abnormal synaptic morphologies at MN2a‐ or MN3‐derived axon terminals termed terminal defects (light grey). Each experiment was performed in triplicates. Note, each genetic experiment was performed in parallel to an adequate control experiment using the same driver line crossed to a line that controls expression of either *UAS‐RFP* or *UAS‐GFP^RNAi^*. *P*‐values between two genetic conditions were calculated by a two‐sided Fisher test. *P < 0.05, **P < 0.01, ***P < 0.005.

To investigate the functional role of Ig domain proteins in synaptic matching, we focused on the MN2a. This neuron co‐expresses the homeodomain TFs Dfd and Mirr (but not Scr) and the Ig genes *DIP‐kappa* and *DIP‐gamma* (Fig 6B, Dataset EV1). RNAi‐mediated knockdown of *DIP‐kappa* and *DIP‐gamma* in neural cells caused the MN2a to target the LR muscle (6% and 23%, respectively; Fig 6A and C) and additionally caused terminal defects (41% and 23%, respectively). Interestingly, this effect was similar to the phenotype induced by Dfd RNAi, but less pronounced (Fig 3D and E, 29% mistargeting and 33% terminal defects). This is consistent with the assumption that targeting specificity is mediated not by one but a combination of Ig genes acting downstream of homeodomain TFs. Importantly, ectopic expression of *DIP‐gamma* in neural cells caused the MN3, which normally does not express this Ig gene, to innervate the *DIP‐gamma* expressing MHE (Fig 6A and C), a phenotype we also observed when Dfd was ectopically expressed in MN3 by means of the neural *elav‐*GAL4 driver (Fig 4B). Ectopic expression of other Ig genes that are not expressed in MN2a according to scRNA‐Seq, such as *dpr1 or dpr11*, also showed MN‐specific wiring defects towards alternative target muscles (Fig 6A and C).

Together, these results suggest that homeodomain TF combinations are associated with Ig gene expression, and Ig domain proteins are involved in mediating synaptic target specificity in the neuromuscular system downstream of homeodomain TFs and thereby modulate muscle target selection.

### Homeodomain TFs mediate target specificity in interconnected MNs and muscle cells

**Figure 7:**
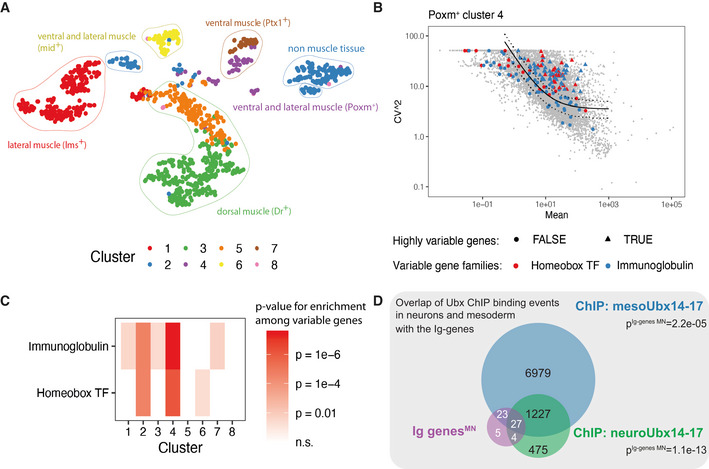
Homedomain TFs are differential expressed in embryonic muscles t‐distributed stochastic neighbour embedding (t‐SNE) plot of single‐cell transcriptomes of GFP expressing somatic muscle cells sorted from *Mhc‐TAU‐GFP* expressing stage 16 *Drosophila* embryos. Colours correspond to clusters identified using hierarchical clustering that was annotated using marker gene expression of muscle subtypes.Identification of highly variable genes using the method by (Brennecke *et al*, 2013). Scatter plot depicts for each gene the mean expression and squared coefficient of variation across cells using the Poxm^+^ cluster as input. The solid line indicates the fit, dashed lines the 95% confidence interval. Genes with a significantly elevated variance are shown as triangles, other genes as circles. Different gene classes are colour‐coded.Statistical model of Brennecke *et al* (2013) was used to identify highly variable genes. Colour code displays enrichment of Ig and homeodomain TF‐encoding genes among the variable genes according to a hypergeometric test.Venn diagram displaying an overlap of Ig domain genes bound by Ubx in muscle and neuronal tissues, identified in a tissue‐specific ChIP‐Seq (Domsch *et al*, 2019). *P*‐value was calculated using a hypergeometric test using as a reference all protein coding genes *n* = 13,920.

The homeodomain TF Dfd is expressed in functionally connected cells of the feeding motor unit, the MN2a and the MHE muscle, while the adjacent LR and MHD muscles do not express Dfd (Fig 4A). Similarly, MN3 and the MHD muscle express the homeodomain TF Scr, whereas the more anterior located muscles and neurons are devoid of Scr expression (Figs 2D and 4A). This observation led us to hypothesize that combinations of homeodomain TFs label matching synaptic partners at defined positions along the AP axis to control synaptic matching. To examine this hypothesis and complement the dataset on MNs, we performed scRNA‐Seq of embryonic somatic muscles with enrichment for *Hox* genes as described above for MNs. To this end, we sorted somatic muscles using a fly stock expressing endogenously *GFP* tagged *Myosin heavy chain* (*Mhc‐TAU‐GFP*, (Chen & Olson, 2001). Analysis of this dataset by means of t‐SNE and clustering indicates the existence of six relatively distinct subtypes of somatic muscle (Fig 7A). Using previously described marker genes, these were tentatively identified as dorsal somatic muscles (Dr positive), lateral somatic muscles (lms positive), ventral and lateral somatic muscles (mid and Poxm positive) and ventral somatic muscles (Ptx1 positive). For further analysis, we focused on the Poxm‐positive cluster and demonstrated that homeodomain TFs and Ig domain encoding genes were highly variably expressed within this rather homogeneous somatic muscle subtype (Fig 7B). Most other clusters showed similar results (Fig 7C). Along the same line, we found binding of the homeodomain TF Ubx close to Ig genes expressed in mesodermal cells of late‐stage embryos when analysing previously generated tissue‐specific Ubx ChIP dataset (Domsch *et al*, 2019) (Fig 7D). These findings strongly suggest that homeodomain TF combinations are associated with Ig gene expression in MNs as well as in muscles.

**Figure 8:**
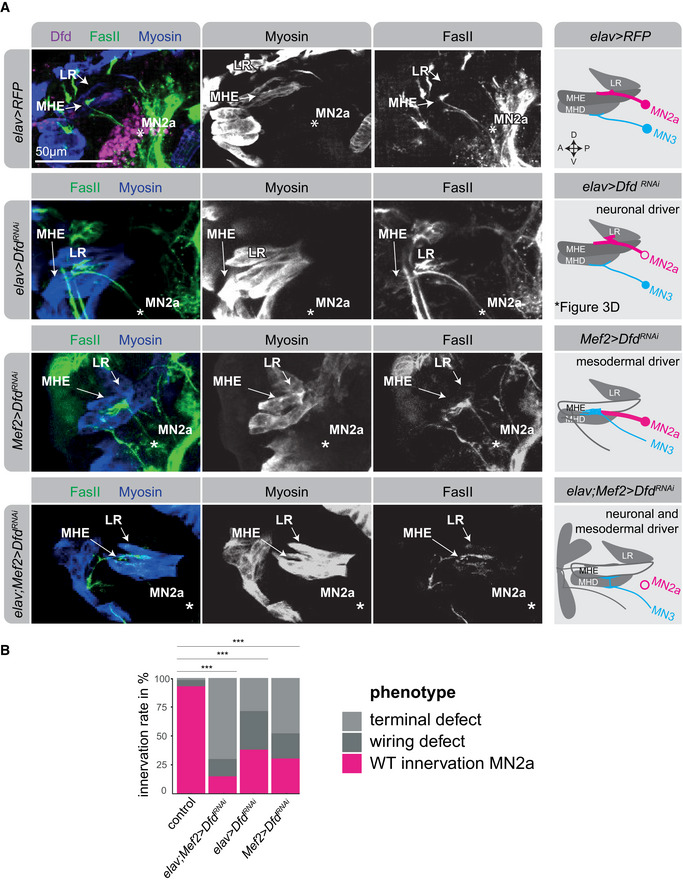
Synaptic targeting requires the homeodomain TF Dfd in neurons and muscles Representative confocal images of stage 17 embryonic heads highlighting the expression of Dfd (purple), Myosin in muscles (blue) and FasII in axonal projections (green) in control (*elav > RFP*) animals and in animals with reduced *Dfd* expression in neurons (*elav > Dfd^RNAi^*) or in muscles (*Mef2 > Dfd^RNAi^*) or both in both tissues (*elav;Mef2 > Dfd^RNAi^*). The single channels for the projections (FasII staining) of MN2a and/or MN3 to the MHE, MHD or LR muscles of an early‐stage 17 *Drosophila* embryo are shown. Asterisks highlight the location of MN2a and MN3, respectively. MN2a is identified by the FasII‐expressing axonal projection emerging from a Dfd‐expressing MN, which normally innervates the Dfd‐expressing MHE muscle (as shown in the *elav > RFP* control), while MN3 is identified as the MN underneath MN2a, which normally innervates the MHD muscle (as shown in the *elav > RFP* control). The panel on the right side represents a schematic drawing of the confocal image shown on the left side, summarizing the innervation of the anterior muscles (LR, MHE, MHD) by projections emerging from MN2a (magenta) or MN3 (cyan) in control and perturbation conditions.Quantitative analysis of phenotypes. Each experiment was performed in triplicates, innervation rates were calculated from *n* = 21 for *elav > Dfd^RNAi^*, *n* = 23 for *Mef2 > Dfd^RNAi^ n* = 20 for *elav;Mef2 > Dfd^RNAi^* animals. Note, each genetic experiment was performed in parallel to an adequate control experiment using the same driver line crossed to a line that controls expression of either *UAS‐RFP* or *UAS‐GFP^RNAi^*, *P*‐values between two genetic conditions were calculated by a two‐sided Fisher test. ***P < 0.005.

To support the hypothesis that co‐expression of identical homeodomain TFs in interconnected cells is critical for synaptic matching, we interfered with *Dfd* in neurons (*elav*‐GAL4) or muscles (*Mef2*‐GAL4) or in both tissues (*elav*‐GAL4; *Mef2*‐GAL4;) and analysed the innervation of anterior muscles (LR, MHE and MHD) by anterior MNs. In all three conditions, we observed significant synaptic wiring defects and terminal defects with a significant increase in phenotypes when *Dfd* was knocked down in both tissues. While neural *Dfd* depletion led to mistargeting of the MN2a to the LR muscle (Fig 8A and B), *Dfd* knock‐down in muscles changed the innervation of the MHE muscle, which normally expresses Dfd, as it was now targeted by MNs that normally innervated the MHD muscles (Fig 8A and B). Knockdown of *Dfd* in both muscle and neurons caused aberrant innervations of the MHE muscles and loss of the stereotypic innervation (Fig 8A and B).

In sum, the results are in line with the hypothesis that combinations of homeodomain TFs are required to align synaptic partners in the neuronal and muscle tissues and coordinate their preferences in synaptic partner choice.

## Discussion

Each neuron in the nervous system chooses a single target cell from a high number of possible interactions, which is a prerequisite for the formation of stereotypic neuronal circuits. This extraordinary degree of precision is thought to be mediated by a matching set of cell specifically expressed cell recognition and adhesion molecules such as CSPs (Sanes & Zipursky, 2020). However, up to date, a systematic scrutinization of such a “connectivity map” and the upstream mechanisms fine‐tuning its expression are missing. The most important reasons for this are the lack of single cell‐specific neuronal markers, the complexity of neuronal systems and the possibly gradual and combinatorial nature of CSP expression and function.

Our experimental design allowed us to overcome these challenges. First, by focusing on one neuronal subtype, the motoneuronal population in *Drosophila* embryos, we were able to reduce neuronal complexity. Second, we investigated and manipulated this cell population exactly at the time when they form the first stereotypic connections with their muscle targets during embryogenesis without affecting earlier stages of development. This approach allowed us to identify molecular cues critical for synaptic wiring, as molecules driving this process are known to be most differentially expressed at that time (Li *et al*, 2017). Third, MNs form highly cell‐specific connections with muscles and are present in a relatively small number per embryo. Thus, we used a single‐cell genomic approach with a high number of biological replicates of every biologically unique cell. Thereby, we identified novel markers specific to single cells or small groups of cells that in turn permit the identification of transcriptome signatures relevant for wiring. And finally, we implemented a spatial mapping approach based on *Hox* gene expression to locate MNs along their AP position and thereby gained insight into the role of spatial mechanisms during synaptic wiring.

Our scRNA‐Seq data revealed that a cell‐specific combination of homeodomain TFs acts as a major component of transcriptional heterogeneity during the wiring phase of MNs. Despite the use of a high number of biological replicates for each unique cell, scRNA‐Seq on its own cannot be applied to unanimously identify biologically unique cells. Thus, we used imaging to demonstrate in four cases a correspondence between homeo‐clusters identified by scRNA‐Seq and gene expression patterns in stereotypically defined single cells. Additionally, we showed that ectopic expression of homeodomain TFs caused wiring defects in MNs not expressing the homeodomain TF, whereas knockdown caused wiring defects in MNs expressing the homeodomain TF. Together, these data allowed us to conclude that cellular heterogeneity within postmitotic *Drosophila* MNs could be described by cell‐specific combinations of homeodomain TFs, which we showed to control late events in neuronal differentiation, in particular synaptic wiring, at the single‐cell level. Thus, our unbiased genomic approach suggested that homeodomain TFs could be major drivers in the synaptic wiring process, which is in line with many previous studies showing that this TF class plays an important role in MNs in different organisms (Thor *et al*, 1999; Jurata *et al*, 2000; Thor & Thomas, 2002; Sanguinetto *et al*, 2008; Dasen & Jessell, 2009; Arber, 2012; Philippidou *et al*, 2012; Zarin *et al*, 2014; Meng & Heckscher, 2020; Hobert, 2021).

Homeodomain TF expression is not only highly specific to individual MNs, but is also associated with profound differences in the entire transcriptome and in particular the expression of Ig CSPs as possible effectors of synaptic specificity. Thus, we used homeodomain TF expression as classifiers to define cellular identities and systematically investigate the distribution of CSPs between cells. We found on average 5–10 homeodomain TF genes and 20–40 Ig genes to be cell specifically expressed in individual MNs, revealing that Ig CSP expression is highly combinatorial and even more complex than the expression of the corresponding homeodomain TFs. Although reported previously that synaptically connected neurons express multiple ligand–receptor pairs (Tan *et al*, 2015), our data suggest that this process is maybe even more complex than previously thought. Interestingly, most CSPs change more gradually between cells, and unlike in the case of homeodomain TF expression, binary expression patterns of Ig genes were not observed in the data (Wit & Ghosh, 2016; Oostrum *et al*, 2020). In line, previous studies on single molecules already indicated that small changes in relative expression levels of CSPs in matching partners can change synaptic specificity (Sweeney *et al*, 2011; Yogev & Shen, 2014). In addition, we found in our dataset, some Ig genes, such as *Dscam* encoding genes, to be broadly expressed but not present in some defined cells, while many other Ig genes are less specific for cellular identities. Importantly, only one Ig molecule class, the DIP genes, were found to be distinct for specific cellular identities. Intriguingly, previous expression and functional studies had already implicated these molecules as strong candidates for synaptic targeting function (Carrillo *et al*, 2015; Tan *et al*, 2015; Ashley *et al*, 2019; Cheng *et al*, 2019), and we now provide additional evidence that they are involved in controlling synaptic matching in the embryonic neuromuscular system. Taken together, our data strongly support a model whereby a highly combinatorial Ig gene expression program active in single cells drives synaptic specificity and connectivity between cells. This complexity might explain why null mutants in many genes contributing to specificity have been shown to result in low penetrance or incomplete phenotypes (Xu *et al*, 2018, 2019). Many recognition molecules might function redundantly in the synaptic matching process either by acting in parallel pathways or in protein complexes. We envision such a complexity to ensure a higher robustness in synaptic matching even when single components are missing or expressed at reduced levels. In the future, it will be important to study the contribution of CSP combinations to synaptic wiring at the single‐cell level to resolve redundancy and specificity within this system.

How is the complex CSP expression program critical for synaptic wiring controlled in individual MNs? Several lines of evidence indicate that combinations of homeodomain TFs are the direct upstream regulators of CSPs in individual MNs. It has been shown before for some homeodomain TFs that they control the precise matching of MNs and their target muscles (Jurata *et al*, 2000; Thor & Thomas, 2002; Sanguinetto *et al*, 2008; Dasen & Jessell, 2009; Arber, 2012; Philippidou *et al*, 2012; Zarin *et al*, 2014; Meng & Heckscher, 2020; Hobert, 2021). In addition, some previous data provided already evidence for a functional link between the expression of receptor molecules and homeodomain TFs (Labrador *et al*, 2005). We now show that manipulation of homeodomain TF expression during the wiring phase by knockdown or ectopic expression leads to changes in target preferences in line with the expression pattern of the respective TF. For example, mis‐expression of more anterior *Hox* genes guided MNs to innervate an anterior muscle, while mis‐expression of posterior *Hox* genes guided MNs to innervate a posterior muscle. Importantly, phenotypes induced by knockdown or overexpression of one Hox TF are phenocopied by manipulations of its putative Ig CSP targets. Consistently, we found the expression of Ig CSP encoding genes to be changed in the absence of homeodomain TFs. Furthermore, chromatin immunoprecipitation studies indicate a direct interaction of homeodomain TFs with control regions of their Ig targets. Together, the combination of single‐cell genomics (co‐expression), chromatin immunoprecipitation (binding of enhancers) and genetics (phenocopies, changes in expression of downstream genes) strongly suggest that cell‐specific homeodomain TF programs are important regulators of synaptic specificity and that combinations of Ig proteins act directly downstream of homeodomain TFs to mediate their function. How these complex combinations of Ig molecules are controlled in a cell‐specific manner by homeodomain TFs will be an important problem to solve in the future. This is particularly puzzling for a TF class like the homeodomain TFs, which interact with highly similar DNA sequences (Ekker *et al*, 1994; Noyes *et al*, 2008). Whether other TF classes, although not identified as highly variable genes but expressed in our MN transcriptomes, contribute to specificity in Ig gene expression will be one of the many paths to follow based on the resources generated in this study. Our analysis also revealed that homeodomain TFs, although co‐expressed in the same MN, can have different functions in synaptic wiring, as their knock‐down does not necessarily result in the same wiring defects (Fig 3). This furthermore highlights the combinatorial nature of regulating synaptic specificity. In future, it will be highly relevant to resolve this combinatorial control on a single‐cell level. Finally, we would predict that synaptic wiring defects resulting from the loss of one homeodomain TF(s) cannot be rescued by the expression of a single Ig target but very likely requires changes in the expression of multiple of these molecules.

**Figure 9:**
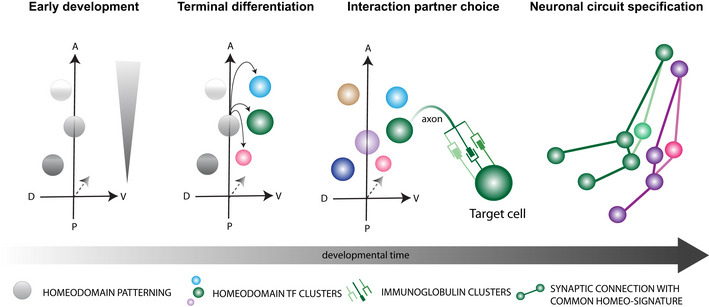
Model depicting the molecular mechanism for stereotypic synaptic partner selection Left panel: Early developmental programs pattern single cells according to their position in the embryo. Morphogenic gradients establish specific homeodomain protein expression patterns along embryonic body axes (i.e. dorsal ventral axis = DV axis, anteroposterior axis = AP axis). Left middle panel: After neuroblasts have undergone several divisions, neurons terminally differentiate and establish unique identities depending on their homeodomain TF expression (colour code). Right middle panel: A unique homeodomain TF expression specifies Ig domain receptor expression in matching synaptic partners, resulting in differential affinities of Ig domain proteins promoting selective synaptic target choice. Right panel: Common homeo‐signatures regulate interaction of partners within individual neuro‐muscular circuits.

In sum, we propose a model whereby the position of every cell is imprinted early in embryonic development by patterns of homeodomain TFs (Fig 9). Expression patterns of these factors become more complex and combinatorial with each cell division until small groups of cells and possibly every single cell are uniquely defined by a homeodomain TF program. These unique combinations of homeodomain TFs then in turn regulate specific downstream programs of Ig gene expression. In the target cells (here: muscles), a similar TF combination is an important determinant of connectivity and possibly regulates the expression of a complementary Ig receptor expression program. This molecular logic enables every single cell to find its corresponding interaction partner based on complementary adhesive properties mediated by combinations of Ig domain molecules. Thus, this concept explains how a molecular memory of cell body position is translated into invariant cell–cell adhesion events by means of a linked homeodomain TF–Ig program. In the future, the combinatorial manipulation of CSPs and homeodomain TFs in single cells (Replogle *et al*, 2020) will allow a further functional dissection of their interactions and regulatory mechanisms.

## Materials and Methods

### Reagents and Tools table

**Table:** 

Reagent/Resource	Reference or source	Identifier or Catalog Number
**Experimental Model: *Drosophila melanogaster***		
UAS‐EGFP RNAi (y[1] sc[*] v[1]; P{y[+t7.7] v[+t1.8] = VALIUM20‐ EGFP.shRNA.3}attP2)	Bloonington Drosophila Stock Center	41560
UAS‐mirr RNAi (y[1] sc[*] v[1]; P{TRiP.HMC06139}attP2)	Bloonington Drosophila Stock Center	65877
UAS‐hth RNAi ( P{y[1] sc[*] v[1]sev21; TRiP.HMS01112}attP2)	Bloonington Drosophila Stock Center	34637
UAS‐DIP gamma RNAi (y[1] sc[*] v[1]; P{TRiP.HMS06062}attP40)	Bloonington Drosophila Stock Center	80461
UAS‐DIP kappa RNAi (y[1] sc[*] v[1]; P{TRiP.HM04050}attP2	Bloonington Drosophila Stock Center	31740
UAS‐Dfd RNAi (UAS‐Dfd‐si on chr.III)	Friedrich et a (2016)	N/A
UAS‐mCD8::GFP (y[1] w[*]; P{w[+mC] = UASmCD8::GFP.L}LL5, P{UAS‐ mCD8::GFP.L}2)	Bloonington Drosophila Stock Center	5137
UAS‐mRFP ( w[1118]; P{w[+mC]=UAS‐myr‐mRFP}1)	Bloonington Drosophila Stock Center	7118
UAS‐lab (w[1118]; P{w[+mC]=UAS‐lab.M}X2)	Bloonington Drosophila Stock Center	7300
UAS‐dpr1 (y[1] w[*]; P{UAS‐dpr1.N}2/CyO)	Bloonington Drosophila Stock Center	25081
UAS‐Scr (w[1118]; P{w[+mC]=UAS‐Scr.M}EE2/TM6B, Tb[+])	Bloonington Drosophila Stock Center	7302
M{UAS‐DIP‐γ.ORF.3xHA.GW}ZH‐86Fb	Flyorf	F003086
M{UAS‐dpr11.ORF.3xHA.GW}ZH‐86Fb	Flyorf	F003032
UAS‐Dfd‐HA	Katrin Domsch (designed for this study)	N/A
elav‐Gal4 (P{w[+mW.hs]=GawB}elav[C155])	Bloonington Drosophila Stock Center	458
Mef2‐Gal4	Ranganayakulu *et al* (1998)	N/A
elav‐Gal4; Mef2‐Gal4	designed for this study	N/A
Ok6‐Gal4	Aberle *et al* (2002)	N/A
Ok371‐Gal4	Mahr and Aberle (2006)	N/A
lab::GFP (y[1] w[*]; P{y[+t7.7] w[+mC]=lab‐GFP.FPTB}attP40)	Bloonington Drosophila Stock Center	66551
Frq1MI00709‐GFSTF (y[1]w[*]; Mi{PT‐GFSTF.0}Frq1MI00709‐GFSTF.0)	Bloonington Drosophila Stock Center	60284
mirr‐GFP (y[1]w[*]; P{mirr‐GFP.FPTB}attP40)	Bloonington Drosophila Stock Center	68183
MHC‐TAU‐GFP	Chen and Olson (2001)	N/A
**Antibodies**		
Rabbit anti‐Hth	Richard Mann lab (McKay *et al*, 2009)	N/A
Rabbit anti‐GFP	invitrogen	A11122
Rabbit anti‐Lab	Thomas Kaufmann lab (Diederich *et al*, 1989)	N/A
Rat anti‐Myosin	abcam	ab51098‐100
Rat anti‐GFP	chromotek	3H9
Rat anti‐Vvl	W.A Johnson lab (Anderson *et al*, 1995)	N/A
Rat anti‐ELAV	Developmental Studies Hybridoma Bank ‐ DSHB	7E8A10
Goat anti‐Abd A	Santa Cruz	27063
Guinea pig anti‐Dfd	Katrin Domsch (designed for this study)	N/A
Guinea pig anti‐Ubx	Katrin Domsch (Domsch *et al,* 2019)	N/A
Guinea pig anti‐Vnd	Robert Zinzen	N/A
Mouse anti‐Scr	Developmental Studies Hybridoma Bank ‐ DSHB	6H4.1
Mouse anti‐Abd A (all isoforms)	Developmental Studies Hybridoma Bank ‐ DSHB	Ubx/ABD‐A FP6.87
Mouse anti‐And B	Developmental Studies Hybridoma Bank ‐ DSHB	1A2E9
Mouse anti‐Antp	Developmental Studies Hybridoma Bank ‐ DSHB	8c11
Mouse anti‐FasII	Developmental Studies Hybridoma Bank ‐ DSHB	1D4
**Oligonucleotides and sequence‐based reagents**		
oligo‐dt30VN	This study	AAGCAGTGGTATCAACGCAGAGTACT30VN
TSO	This study	AAG CAG TGG TAT CAA CGC AGA GTA CAT rGrG+G
IS PCR primer	This study	AAG CAG TGG TAT CAA CGC AGA GT
targeted_Dfd_RT_rv	This study	TCG GAT TGT TGC TGT TGA AG
targeted_Ubx_RT_rv	This study	CAG AAT TTT GCT CGC ATT CA
targeted_AbdA_RT_rv	This study	CAT GCG TTG CTC TAT CAA A
targeted_AbdB_RT_rv	This study	AAT ATA ATG CTC GGG GCA AA
targeted_Scr_RT_rv	This study	ATT GGG CGA TAC AAA CGA AG
targeted_Lab_RT_rv	This study	CCC TTC AAC TTT GCT TGC TC
targeted_Antp_RT_rv	This study	AAC CAT ACC CAG TCC ACC AA
targeted_Dfd_IS_fw	This study	AAG CAG TGG TAT CAA CGC AGA GTC CCT GGA TGA AGA AGA TCC A
targeted_Ubx_IS_fw	This study	AAG CAG TGG TAT CAA CGC AGA GTA AGG AGC TGA ACG AAC AGG A
targeted_AbdA_IS_fw	This study	AAG CAG TGG TAT CAA CGC AGA GTC TGG ACA AGA GCA ATC ACG A
targeted_AbdB_IS_fw	This study	AAG CAG TGG TAT CAA CGC AGA GTC GGA TTC GAT TTT AGC AAA TG
targeted_Scr_IS_fw	This study	AAG CAG TGG TAT CAA CGC AGA GTT CGA ATG CAA CTT GTT CTG C
targeted_Lab_IS_fw	This study	AAG CAG TGG TAT CAA CGC AGA GTC CCT GAT AAT GGC GAA CAG T
targeted_Antp_IS_fw	This study	AAG CAG TGG TAT CAA CGC AGA GTA GAG GAA CAG CAA AGC GAA A
targeted_Dfd_IS_rv	This study	AAG CAG TGG TAT CAA CGC AGA GTT CGG ATT GTT GCT GTT GAA G
targeted_Ubx_IS_rv	This study	AAG CAG TGG TAT CAA CGC AGA GTC AGA ATT TTG CTC GCA TTC A
targeted_AbdA_IS_rv	This study	AAG CAG TGG TAT CAA CGC AGA GTC ATG CGT TGC TCT ATC AAA
targeted_AbdB_IS_rv	This study	AAG CAG TGG TAT CAA CGC AGA GTA ATA TAA TGC TCG GGG CAA A
targeted_Scr_IS_rv	This study	AAG CAG TGG TAT CAA CGC AGA GTA TTG GGC GAT ACA AAC GAA G
targeted_Lab_IS_rv	This study	AAG CAG TGG TAT CAA CGC AGA GTC CCT TCA ACT TTG CTT GCT C
targeted_Antp_IS_rv	This study	AAG CAG TGG TAT CAA CGC AGA GTA ACC ATA CCC AGT CCA CCA A
qPCR_act5C_fw	This study	AGG AGG AGG AGG AGA AGT CG
qPCR_act5C_rv	This study	TGT GCT GCA CTC CAA ACT TC
qPCR_Dfd_fw	Hu *et al* (2013)	GCG AAC GGA TCA TCT ACC CC
qPCR_Dfd_rv	Hu *et al* (2013)	ATC TGA TGG CGT GTG TAG GC
qPCR_DIP‐kappa_fw	Hu *et al* (2013)	ATC CCC TCA AAG GGA AAA CAC A
qPCR_DIP‐kappa_rv	Hu *et al* (2013)	GAA TCG CGG AAA GTC GGA ATC
qPCR_DIP‐gamma_fw	Hu *et al* (2013)	AAC CAG CAT CAC GAG AGC AG
qPCR_DIP‐gamma_rv	Hu *et al* (2013)	GCC GGA TAT GTG ACG TTG TTG
qPCR_exd_fw	Hu *et al* (2013)	CGG AGC AAT CAC TTG ACG AGG
qPCR_exd_rv	Hu *et al* (2013)	CGA GAG GAC GGT CTT CTC CTT
**Chemicals, enzymes and other reagents**		
5xSMART First Strand buffer	Clonetech	N/A
Ampure XP beads	Beckman	A63880
DNase I (RNase free)	Invitrogen	AM222
Formaldehyde	Sigma Aldrich	252549
KAPA HiFi HotStart ReadyMix	fisherscientific	NC0295239
oligo (dT)18	Thermo Scientific	S0132
RevertAid First Strand cDNA synthesis Kit	Thermo Scientific	K1622
RNAse Inhibitor	Thermo Scientific	EO0381
SMARTScribe™ Reverse Transcriptase	clonetech	639537
SSC (20X)	Sigma Aldrich	15557044
SYBR green for qPCR	Invitrogen	117733‐038
Tn5	PepCore EMBL	N/A
TRIzol reagent	Ambion by Life Technologies	15596026
VectaShield + DAPI	Vector Laboratories	H‐1200
HCR probe *Vglut_*B1	Molecular Instruments	NM_001273010.1
HCR probe *DIP‐gamma_*B2	Molecular Instruments	NM_143392
HCR probe *DIP‐kappa_*B3	Molecular Instruments	NM_165049, NM_001298975.1
HCR probe *otk_*B4	Molecular Instruments	NM_078981.3, NM_001299396.1
HCR probe *Lim1_*B4	Molecular Instruments	NM_132277.3, NM_001272429.2
HCR probe *unc4_*B5	Molecular Instruments	NM_001298460.1, NM_133014.3, NM_001298461.1
HCR probe *twit_*B2	Molecular Instruments	NM_136223
HCR probe *Vmat_*B3	Molecular Instruments	NM_001014524.2, NM_001014525.3, NM_001014526.2, NM_001274027.2
**Software**		
Fiji (ImageJ)	Schindelin *et al* (2012)	https://imagej.net/Fiji
IndeXplorer	Velten *et al* (2017)	https://git.embl.de/velten/indeXplorer
iRegulon	Janky *et al* (2014)	http://iregulon.aertslab.org/
**Other**		
Illumina NextSeq platform	Illumina	
qTOWER3	Analytik Jena	
Leica TCS SP8 confocal microscope	Leica	

### Methods and Protocols

#### *Drosophila* strains and experimental crosses

We used the *OK371*‐GAL4 driver (Mahr & Aberle, 2006) crossed to UAS‐*mRFP* to perform single cell sorting of MNs and the *Mhc‐TAU‐GFP* line (Chen & Olson, 2001) for sorting of somatic muscle cells. Crosses were kept for 1 h at 25°C to oviposit on apple juice plates with yeast paste. Subsequently, eggs were incubated on apple juice plates for additional 19 h, after which embryos were dissociated for FACS sorting.

In order to validate protein expression of specific marker genes, we used the following endogenously GFP‐tagged fly lines: *frq1::GFP* (BL:60284), *mirr::GFP* (BL:68183) and *lab::GFP* (BL:66551) lines.

For genetic experiments, we used the pan‐neural *elav‐*GAL4 driver (Luo *et al*, 1994) and the muscle‐specific *Mef2‐*GAL4 driver (Ranganayakulu *et al*, 1998) as main driver. In addition, we combined these two driver lines (*elav*‐GAL4; *Mef2*‐GAL4 driver) for this study. To confirm phenotypes, in MNs, the *OK6‐*GAL4 driver (Aberle *et al*, 2002) was used in a few examples. In this study, we used second‐generation *TRiP RNAi* lines from Bloomington (Ni *et al*, 2011), as we found that these *short hairpin RNAs* (*shRNAs*) are more effective in embryonic stages. Therefore, only one UAS‐*RNAi* line fulfilling the required criteria has been available for most of the target genes. The UAS‐*Dfd^RNAi^* was designed for this study using the second generation of TRiP system. The UAS‐*mirr^RNAi^ (BL:65877)*, UAS‐*hth^RNAi^ (BL:34637)*, UAS‐*DIPgamma^RNAi^ (BL:80461)*, UAS‐*DIPkappa^RNAi^(BL:31740)* were obtained from the Bloomington Stock Center. The UAS overexpression lines UAS‐*Ubx*‐HA (Domsch *et al*, 2019) and AS‐*Dfd*‐HA were designed by Katrin Domsch as described in (Domsch *et al*, 2019), the UAS‐lab and UAS‐*dpr1 (BL:31740)* lines were from the Bloomington Stock Center, and the UAS‐*DIPgamma (F003086)* and UAS‐*dpr11 (F003032)* lines were from the FlyORF collection. For innervation rate assays, we crossed virgins of the corresponding driver line to males carrying UAS‐*RNAi* or UAS overexpression constructs. As control, we performed the experiment under the same conditions with UAS‐*mRFP* lines or UAS‐*EGFP^RNAi^* lines. The crosses were kept for at least 16 h at 29°C for knock‐down experiments and 20 h at 25°C for overexpression experiments on apple juice plates. Early‐stage 17 embryos were selected after embryo fixation.

For hatching rate assays, the *elav*‐GAL4 driver and *Mef2*‐GAL4 drivers were crossed to UAS‐*RNAi* lines. UAS‐*mRFP* lines or UAS‐*EGFP^RNAi^* lines crossed to the above‐mentioned driver lines were used as controls. Crosses were set up in duplicates (equal amounts of males and females for every replicate, sample and control), and egg laying was performed at 29°C for 3 h on apple‐juice plates with yeast paste. Subsequently, eggs were washed, counted and unfertilized eggs were removed or counted and transferred on fresh apple juice plates without yeast paste. Eggs were incubated for additional 24 h and then the hatching rate was quantified.

#### Plasmid construction, transgenesis and antibody production

The UAS‐*Dfd^RNAi^* line was generated following the conventional TRiP protocol (second generation).

UAS‐HA‐*Dfd* fly line: The full *Dfd* coding region was cloned into the pUAS‐attB vector (Bischof *et al*, 2007) using a forward primer with an *EcoR*I restriction site and an HA tag sequence as well as a reverse primer with an *Xba*I restriction site. The UAS‐*Dfd* construct was injected by BestGene into attP5 embryos (second chromosome). Primers and sequence maps are available upon request. Dfd antibody: The Dfd antibody was generated using the pGEX‐purification system (GElifesciences). The open reading frame of *Dfd* was cloned in the pGEX‐6P‐2 vector using *EcoR*I and *Xho*I restriction site. The protein was purified according to the protocol (GElifesciences) and eluted by using the PreScission Protease site. The immunization and antibody handling were performed by the Charles Rivers Company.

#### Immunohistochemistry

The embryos were fixed by bleaching with 100% Clorox first for 2 min to remove the chorion. After washing in water, embryos were transferred to fixing solution (3.7% formaldehyde in PBS + 100% heptane) and incubated for 20 min at room temperature on a nutator. Fixing was stopped by removal of formaldehyde. Then, equal amounts of methanol were added to heptane and vortexed for about 40 s to remove the vitellin membrane. Subsequently, the heptane phase was removed, and embryos were washed in methanol.

For antibody stainings, embryos were hydrated and washed for three times in PBT (with 0.1% Tween 20). The primary antibodies were used at 4°C overnight from Abcam (rat anti‐Myosin 1:1,000) DSHB (mouse anti‐FasII, 1:50; mouse anti‐Scr, 1:50; mouse anti‐Abd A, 1:50; mouse anti‐Antp; rat anti‐Elav 1:50, mouse anti‐AbdB 1:50), Invitrogen (rabbit anti‐GFP, 1:500), provided by Katrin Domsch (guinea pig anti‐Dfd 1:500), guinea pig anti‐Ubx (1:500) (Domsch *et al*, 2019), rabbit anti‐Lab was given by T. Kaufmann, rabbit anti‐Hth provide by R. Mann and the rat anti‐Vvl was given by the Johnson lab. After 3× washing in PBT (with 0.1% Tween 20), embryos were incubated for 2 h at room temperature with secondary antibodies from Jackson Immunoresearch. Vectashield with DAPI or TSO was used as mounting medium.

For double stainings with antibodies originating from the same animal (mouse anti‐FasII + mouse anti‐Antp; mouse anti‐FasII + mouse anti‐Abd A; mouse anti‐FasII + mouse anti‐Scr), we performed a modified protocol for sequential antibody staining by TSA (according to the manufacture protocol). Primary and secondary antibodies were used with similar concentrations than for antibody staining's of embryos.

#### *In situ* hybridization chain reaction (HCR)

Multiplex *in situ* hybridization experiments were performed on fixed late‐stage *Drosophila* embryos according to the HCR version 3.0 protocol (Choi *et al*, 2018) and personal communication with Christoph Schaub. Customized probes were generated for this study by Molecular Instruments targeting all isoforms of each target gene (*VGlut* B1, *DIP‐gamma* B2, *DIP‐kappa* B3, *otk* B3, *Lim1* B4, *unc4* B5, *twit* B2, *Vmat* B3). A maximum of three targets were multiplexed in the same *in situ* hybridization experiment or the *in situ* hybridization protocol was combined with the immunohistochemistry protocol.

In brief, late‐stage embryos were collected, bleached and fixed in 3.7% formaldehyde in PBS + 100% heptane. Then, the vitelline membrane of embryos was removed by vortexing and the embryos were dehydrated in methanol. For *in situ* hybridization experiments, embryos were first prehybridized and then hybridized at 37°C with different combinations of customized probes with corresponding adaptors in 30% probe hybridization buffer (30% formamide, 5xSSC, 9 mM citric acid pH6, 0.1% Tween20, 50 µg/ml heparin, 1× Denhardt’s solution, 5% dextran sulphate). After washing in 5× SSCT (5× SSC, 0.1% Tween20, 5% dextran sulphate), fluorescently labelled hairpins were pre‐amplified for each linker (B1, B2, B3, B4, B5) in amplification buffer (5xSSC, 0.1% Tween20, 5% dextran sulphate). Prepared linkers were incubated with the embryos at room temperature. After washing, samples were either mounted for microscopy or an additional immunohistochemistry protocol followed as described in the section above.

#### Quantitative PCR

Early‐stage 17 embryos were subjected to RNA extraction using TRIzol. RNA was then isolated by chloroform/isopropanol extraction. After DNA digestion (DNase I, Invitrogen), 1 μg of total RNA was converted to cDNA using the RevertAid First Strand cDNA synthesis Kit (Invitrogen) and oligo(dT)_18_ primers (Fisher Scientific). Quantitative PCR was performed using the SYBR Green Master Mix (Invitrogen), 150 nM primer, in 96‐well plates, conducted on the qTOWER3 machine (Analytik Jena).

#### Microscopy and image analysis

Fixed embryos were imaged on a Leica TCS SP8 confocal microscope. The 20× objective was used for imaging of embryos and the 63× objective to visualize the neuromuscular junction of the MHE muscle. For homeodomain TF cluster analysis, we used as reference the highly stereotypic neuronal projection from the MN2a to MHE and the MN3 to MHD labelled by the motoneuronal marker FasII. Going from MN2a, we identify the upper (MN2c) and lower (MN4) MNs based on the location. Images were processed by Fiji and the integrated ROI manager was used to label and save locations of cells and projection based on FasII, these labels were transferred across pictures. To enable precise localization across different confocal images, we used the Fijiyama plugin for 3D alignment. Embryos used for quantification of innervation rate assays were processed with a standardized imaging pipeline to facilitate counting (programed by Patrick van Nierop Y Sanchez, see https://github.com/patrickvannieropysanchez/Velten_et_al._2021). Confocal pictures shown in figures were processed manually to optimize the result.

For AP axis measurements along the ventral nerve cord, we used FasII staining as reference and measured protein intensities of Lab, Dfd, Scr, Antp, Ubx, Abd A and Abd B by Fiji. We normalized the length of the ventral nerve cord between different embryos (0 = anterior to 2000 = posterior), set thresholds for the protein intensities to remove the overall unspecific background caused by antibody stainings and normalized the protein intensities of different the antibodies (0=min intensity to 100=max intensity). For counting motoneuron numbers, 20x resolution was used to orientate the embryos with help from the Fiji plugin “clear volume”, cells were counted at 63× resolution using the Fiji multiple point tool. Vnd antibody staining was used to identify segment boundaries in the embryonic ventral nerve cord (McDonald *et al*, 1998).

#### Dissociation of embryonic cells and flow cytometry

Collections of 19‐ to 20‐h‐old *Drosophila* embryos were dechorionated with bleach (5% Chloride) for 2 min. For dissociation, embryos were transferred into syringes filled with 1ml of 10x Trypsin‐EDTA (0.5% Trypsin). For additional mechanical dissociation, embryos were transferred 20x between two syringes (25G needle) and 10× between two syringes with 27G needles. Debris was reduced by filtering through a 35‐μm cell strainer. The cells were stained for 5 min with DRAQ5 (1 mg/ml) and DAPI (1 mg/ml). Subsequently, cells were sorted into 96‐well plates containing 5 µl of Smart‐Seq2 lysis buffer at 4°C by a BD FACS Aria III flow cytometer equipped with 405nm, 488nm, 561nm and 633nm laser. Directly after cell sorting, plates were shock‐frozen in liquid nitrogen.

#### Single‐cell transcriptome sequencing

A pooled cell population of *OK371 > RFP*‐positive motoneuronal cells and a pooled population of *Mhc‐TAU‐GFP*‐positive somatic muscle cells derived from FACS‐sorted 19‐ to 20‐h‐old *Drosophila* embryos were used for scRNA sequencing. The standard Smart‐Seq2 protocol (Picelli *et al*, 2014) was modified by targeting low‐expressed *Hox* genes (*lab, Dfd, Scr, Antp, Ubx, abdA, AbdB*) during the reverse transcription and PCR stages. Therefore, 1µM targeted primer mix of each *Hox gene* (see key resource table) was added to the PCR and RT buffers, respectively. The above‐described approach targets all expressed isoforms of each *Hox* gene. Method‐specific biases were ruled out by quality checks via Bioanalyzer of the whole transcriptome after Smart‐Seq2 preparation. Libraries of scRNA transcriptomes were created using a home‐made Tn5 transposase (Hennig *et al*, 2018) and seventeen 96‐well plates were sequenced with 75bp single‐end on an Illumina NextSeq platform.

#### Raw data processing, quality control and normalization

Sequencing reads were demultiplexed and the poly‐A tail trimmed. Read count tables were generated by pseudo‐alignment to the cDNA of the *Drosophila* transcriptome (BDGP6 ensemble) using Kallisto (Bray *et al*, 2016). For quality control, we used standard settings (Velten *et al*, 2017): cells were removed unless they contained at least 10 reads for each of at least 500 genes, and genes were removed unless they were expressed in at least five cells with 10 reads each. For basic cell type discovery and PCA, data were normalized and scaled to an estimate of biological variance using the indeXplorer pipeline (Velten *et al*, 2017).

Such scaled and mean‐centred expression values take very high values if the gene is expressed in few cells and low values if the gene is expressed in many cells (see Fig EV4G). Library‐size normalized data emphasize the absolute expression level of the genes. Neither of the two strategies is ideal when aiming to identify a “code” using clustering strategies. Binarization of the gene expression levels would constitute a viable alternative (see for example (Li *et al*, 2017)) but cannot capture biologically meaningful quantitative differences between cells expressing the gene “highly” or “lowly”. For the heatmap depicting the homeodomain TF expression, we therefore normalized the data gene‐wise by

$$N_{i}=\log\left(k\frac{X_{i}}{\sum X}+1\right)$$

where *X* is the vector of size factor normalized counts for a given gene. *N* approaches binarization for large *k*. Here, a value of *k* = 10^6^ was chosen. This leads to a behaviour where normalized values are essentially binarized for lowly expressed genes, where quantitative differences cannot be captured in a meaningful manner; by contrast, for highly expressed genes, quantitative expression levels play a role (see Fig EV4G). We have previously used similarly motivated strategy for data normalization in the context of predicting cell–cell interactions using scRNA‐Seq data (Baccin *et al*, 2020).

#### Clustering and dimensionality reduction

Following the indeXplorer pipeline, PCA was computed using scaled, normalized expression values of all genes. t‐SNE was then computed on the first 10 principal components based on a visual inspection of the PCA Elbow plot. Alternatively, and following (Li *et al*, 2017), cells were clustered by hierarchical clustering using only the 20 most variably expressed genes of our scRNA dataset; Ward linkage was used on an Euclidean distance metric. The *twit^low^* cluster was selected for further analysis on variability in gene expression. To this end, we used the statistical method developed by (Brennecke *et al*, 2013) to identify variable genes. The variable homeodomain TFs thereby identified were normalized as described above and used as input for unsupervised hierarchical clustering using ward linkage and Euclidean distance matrix to avoid that clustering is affected by technical noise. To validate this point, we showed that homeo‐clusters are not significantly associated with technical covariability (sequencing depth) by Wilcoxon test (Fig EV4H). Then, we split the clusters in a supervised manner according to the structure of the dendrogram and compared the approximate number of cells labelled by the *OK371‐*GAL4 driver during this stage with the estimated cluster number.

#### Inference of spatial position from single‐cell gene expression data

To infer spatial position from single‐cell gene expression data, we first created a reference map of protein expression for seven *Hox* genes (Fig EV4A and B). To that end, immunofluorescence experiments were performed as described above.

Immunofluorescence data for gene g were thereby represented as a function of position $Y_{g}\left(x\right)\in \left(0,1\right)$, whereas single‐cell gene expression was represented as a matrix of read count values across genes and cells, $D_{g,c}\in N_{0}$. Cells not expressing any Hox gene were excluded. We then assumed that the probability of observing $D_{g,c}$ given that the position of cell c is really $x_{c}$ depends on the expression of g at that position:

$$p\left(D_{g,c}|x_{c}\right)∼\left\{\begin{array}{cc} Pois\left(D_{g,c}|r_{g}*Y_{g}\left(x\right)\right) & \text{if}\;Y_{g}\text{(}x_{c})>0\\ Pois\left(D_{g,c}\right|\lambda ) & \text{if}\;Y_{g}\left(x_{c}\right)=0 \end{array}\right.$$

here λ is a constant corresponding to a background number of reads observed in non‐expressing cells, and $r_{g}$ is a gene‐wise scaling factor that estimates the average number of RNA‐seq reads in a cell maximally expressing the protein. A maximum likelihood estimate of $x_{c}$ is then obtained by computing

$$\hat{x_{c}}=\underset{x}{\arg\;\max}\prod_{g}p\left(D_{c,g}|x\right)$$

For the analysis shown in the manuscript, λ was set to 0.1 (Kharchenko *et al*, 2014) and $r_{g}$ was set to the mean gene expression in expressing cells:

$$r_{g}=\frac{\sum_{c}D_{c,g}}{\sum_{c}D_{c,g}>0}$$

We used Latin Hypercube sampling across a wide range of values for λ and r to demonstrate that the position estimate was independent of parameter choice.

To identify genes with spatially variable expression, B‐Spline models with 3 degrees of freedom were fitted through scaled and normalized gene expression values for each gene individually, using inferred position as the only covariate. These models were then compared to null models not containing the position term using the Bayesian information criterion, similar to the workflow for selecting genes with variable expression over pseudotime described in (Velten *et al*, 2017).

For visualization and clustering, expression values for all variably expressed genes were arranged by inferred position and a floating mean was computed by 1D‐convolution with an absolute exponential kernel with decay rate 10. Smoothened gene expression values obtained thereby were then compared to immunofluorescence images for model validation (Fig EV4D) or used for clustering of gene into modules with coherent expression patterns over space (Fig 1D and E).

#### ZINB‐WaVE analysis

To identify gene whose expression was variable but statistically independent from the AP axis, we made use of the ZINB‐WaVE model (Risso *et al*, 2018, 2019). Unlike a PCA, ZINB‐WaVE separately estimates a matrix of known‐covariate factors as well as a matrix of unknown‐covariate factors; furthermore, ZINB‐WaVE uses a zero‐inflated negative binomial distribution to account for the sparse nature of single‐cell RNA‐seq data. We ran ZINB‐WaVE using default settings and the number of genes observed as well as the inferred AP position as known sample‐level covariates. While the PCA of the dataset was dominated by effects related to the number of genes observed, viability dye incorporation, and AP position (Fig EV4E), the unknown‐covariate factors from ZINB‐WaVE arranged genes according to their dorsal–ventral expression (Fig 1F). Two of the predicted DV markers were validated by Immunofluorescence (Fig EV4F).

#### ChIP‐Seq reanalysis and iRegulon analysis

To identify the relationship between motoneuronal genes expressed in our scRNA‐Seq dataset and genes bound by the homeodomain TF Dfd, we used a whole embryo Dfd ChIP (Sorge *et al*, 2012) to investigate if Ig domain proteins are overrepresented among putative Dfd‐regulated genes compared to other genes expressed in MNs. Genes were classified as regulated by Dfd when Dfd binding to the target gene was detected max. 1 kb upstream of the promotor region (Sorge *et al*, 2012). To calculate a *P*‐value for enrichment, we performed a hypergeometric test. The Ubx ChIP‐Seq data were analysed as described in (Domsch *et al*, 2019). To illustrate the overlap of neuronal Ubx‐bound genes with the genes from the Ig expression maps, mapped neuronal Ubx peaks were assigned to genes in close proximity and these genes were used in the Venn diagram to illustrate the common and unique populations.

The iRegulon (Janky *et al*, 2014) analysis was used to identify TF motifs enriched in the vicinity of Ig‐encoding genes expressed in our motoneuronal scRNA dataset. Therefore, we performed an iRegulon analysis on all Ig domain encoding proteins expressed in MNs under standard settings (9,713PWM; 5 kb upstream, 50 UTR and first intron with standard cut‐off) to identify the 15 highest ranked TF motifs. We next used iRegulon to investigate which TF families are predicted to bind to these motifs and chose three of the five motifs predicted to be regulated by homeodomain TFs.

#### Data visualization

All plots were generated using the ggplot2 (v. 3.2.1) and pheatmap (v. 1.0.12) packages in R 3.6.2. Boxplots are defined as follows: The middle line corresponds to the median; lower and upper hinges correspond to first and third quartiles. The upper whisker extends from the hinge to the largest value no further than 1.5 * IQR from the hinge (where IQR is the inter‐quartile range, or distance between the first and third quartiles). The lower whisker extends from the hinge to the smallest value at most 1.5 * IQR of the hinge. Data beyond the end of the whiskers are called “outlying” points and are plotted individually.

## Author contributions

**Jessica Velten:** Conceptualization; Validation; Investigation; Methodology; Writing—original draft; Writing—review and editing. **Rashi Agarwal:** Investigation. **Patrick Van Nierop y Sanchez:** Investigation; Methodology; Writing—review and editing. **Katrin Domsch:** Investigation; Methodology; Writing—review and editing. **Lena Bognar:** Investigation. **Malte Paulsen:** Investigation; Methodology. **Lars Velten:** Data curation; Software; Investigation; Methodology; Writing—original draft; Writing—review and editing. **Ingrid Lohmann:** Conceptualization; Supervision; Funding acquisition; Investigation; Methodology; Writing—original draft; Writing—review and editing. **Xuefan Gao:** Data curation; Formal analysis; Validation.

In addition to the CRediT author contributions listed above, the contributions in detail are:

JV and IL designed the study. The experiments were performed by JV with significant contributions by XG, RA, LB and MP. The bioinformatic data analysis was performed by JV and LV and KD. PVN designed a code for image analysis. JV wrote the paper together with IL and LV. IL obtained funding for this study (DFG, LO 844/4‐2). All authors have read and commented on the paper.

## Disclosure and competing interests statement

The authors declare no competing or financial interest.

## Supporting information



Expanded View Figures PDF

Dataset EV1

## Data Availability

Single‐cell transcriptomics raw data and count tables are available from gene expression omnibus, ID: GSE155578 (http://www.ncbi.nlm.nih.gov/geo/query/acc.cgi?acc=GSE155578for the motoneuronal dataset) and ID: GSE155586 (http://www.ncbi.nlm.nih.gov/geo/query/acc.cgi?acc=GSE155586; for the muscle dataset). Most analyses were done using the indeXexplorer software for interactive exploration of single‐cell RNA‐seq datasets (Velten *et al*, 2017), which is available from https://git.embl.de/velten/indeXplorer. Custom scripts for inference of spatial position are available from the corresponding authors upon request.
