# Supplementary material for: Single‐cell RNA sequencing of motoneurons identifies regulators of synaptic wiring in Drosophila embryos
Source: Mol Syst Biol. 2022 Feb 28;18(3):e10255. doi: 10.15252/msb.202110255 (PMC8883443; doi:10.15252/msb.202110255)
Supplement: Supplementary file 1 — Expanded View Figures PDF [file MSB-18-e10255-s001.pdf]

Expanded View Figures

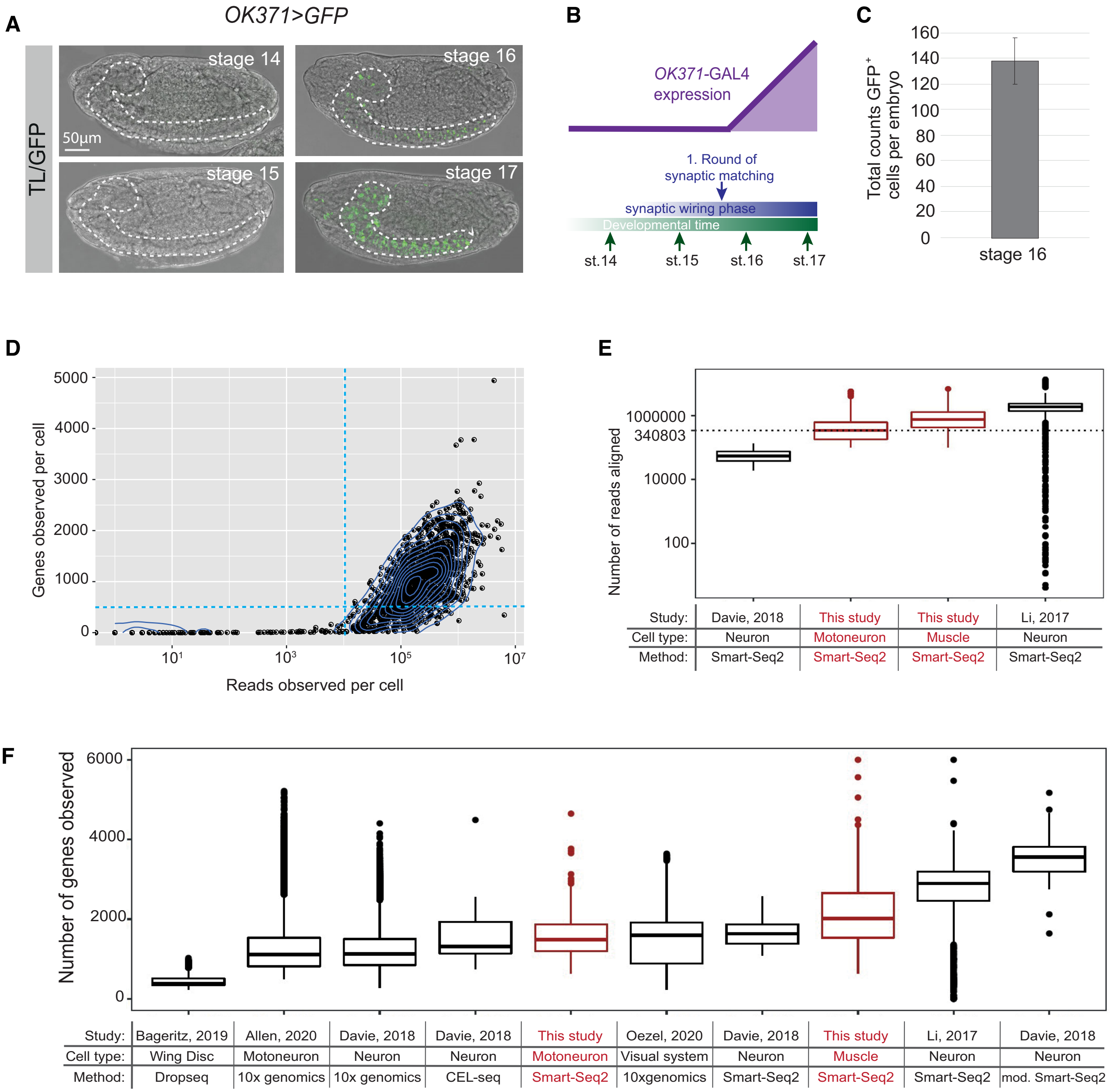

Figure EV1.

 ©2022 The Authors

Figure EV1. scRNA-Seq data of embryonic *Drosophila* MNs are of high quality.

A Time course of GFP expression induced by the *OK371-GAL4* motoneuronal driver during embryonic stages, showing that GFP expression starts after embryonic stages 15 and is clearly detectable at stages 16 and 17 in neuronal cells. Dashed lines highlight the ventral nerve cord.

B Illustration of the time course shown in (A) in relation to the synaptic wiring in the embryonic neuromuscular system, highlighting that *OK371-GAL4*-driven transgene expression occurs at the time when the first synaptic connections are formed between MNs and muscles.

C Quantification of the average number of differentiated *OK371 > GFP*-positive MNs in stage 16 embryos ($n = 3$, biological replicates with two independent biological repeats, error bar denotes standard deviation).

D Visualization of filtering criteria for single cells (dashed blue line, see Materials and Methods). Density dot plot represents the total reads (library size) versus genes observed per cell (library quality, diversity). Each dot represents a motoneuronal cell (total of 1,536 cells). In total, $n = 999$ cells passed the filtering criteria indicated by the dotted lines (see Materials and Methods).

E Number of reads aligned to the *Drosophila* genome per cell for the two datasets from this study (red) and two other studies (black) profiling *Drosophila* neurons by Smart-Seq2. See Materials and Methods, section *Data visualization* for a definition of boxplot elements. Individual data points correspond to single cells (biological replicates), see legend of panel F for number of cells.

F Number of genes observed per cell for the two datasets from this study (red) and several other studies (black) profiling *Drosophila* neurons by scRNA-Seq (Li *et al*, 2017; Davie *et al*, 2018; Bageritz *et al*, 2019; Allen *et al*, 2020; Özel *et al*, 2021). See Materials and Methods, section *Data visualization* for a definition of boxplot elements. Individual data points correspond to single cells (biological replicates). Bageritz *et al*: $n = 2{,}554$, Allen *et al*: $n = 33{,}115$, Davie 10× genomics: $n = 56{,}902$, Davie CEL-seq: $n = 22$, Davie Smart-Seq2: $n = 45$, Davie modified Smart-Seq2: $n = 34$, this study (Motoneuron): $n = 999$, this study (Muscle): $n = 837$, Oezel *et al*: $n = 31{,}018$, Li *et al*: $n = 1{,}842$.

©2022 The Authors

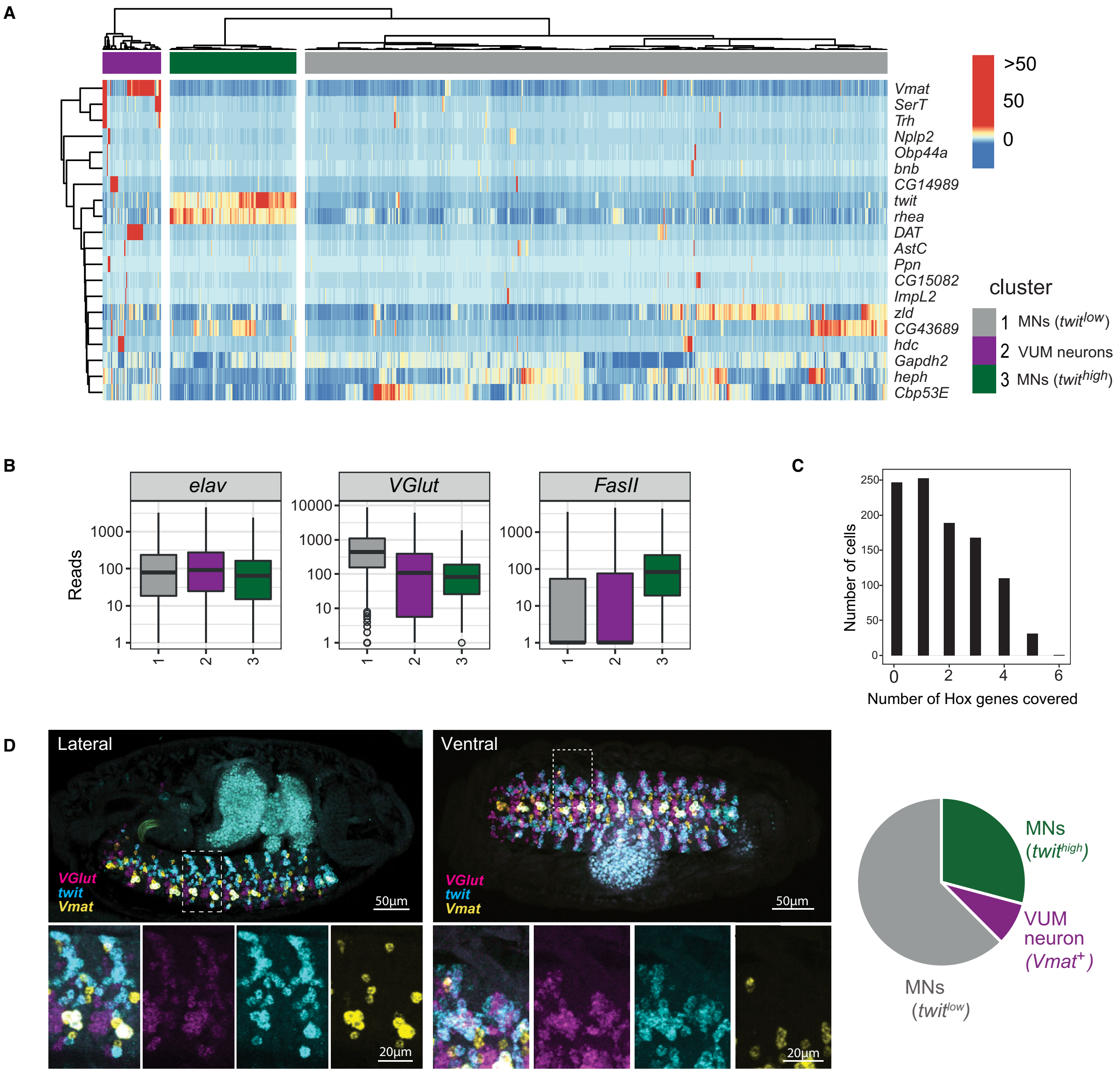

Figure EV2. scRNA-Seq data of embryonic *Drosophila* MNs identifies three major clusters.

A Heatmap depicting gene expression of single motoneuronal cells (columns) after hierarchical clustering using the 20 most variably expressed genes (rows) following the method of Li *et al* (2017). Hierarchical clustering was performed using ward linkage on an Euclidean distance metrics. Colour code represents gene expression levels (see Materials and Methods). Three distinct clusters with similar expression patterns are labelled in grey (cluster 1), purple (cluster 2) and green (cluster 3), these clusters correspond to clusters shown in Fig 1B.

B Expression of three marker genes, *elav* (pan-neural), *VGlut* (glutamatergic MNs) and *FasII* (axon) were evaluated for each of the three clusters shown in (A). See Materials and Methods, section *Data visualization* for a definition of boxplot elements. Individual data points correspond to single cells (biological replicates), $n = 758$ (cluster 1), $n = 76$ (cluster 2), $n = 165$ (cluster 3).

C Bar chart shows the number of cells expressing different numbers of *Hox* genes. In sum, 749 of 999 cells express at least one *Hox* gene (~ 75% *Hox* gene coverage).

D *Left panel:* Multiplex HCR visualizes the expression pattern of *VGlut*, a general marker for glutamatergic MNs, and two key marker genes, *twit* and *Vmat*, which drive the clustering shown in (A). The dashed boxes in the upper panels, which show the lateral and dorsal view of representative embryos, are displayed at higher resolution in the lower panel. *Right panel:* Venn diagram showing ratios of cells labelled with these key marker genes, which are compared with ratios expected from scRNA-Seq experiments.

 ©2022 The Authors

Figure EV3. Detailed analysis of single cell data identifies known MN subtypes and variable processes in stage 16 embryonic MNs.

A *Left panel*: ventral view of representative stage 16 and late-stage 17 embryos expressing UAS-*RFP* under the control of *OK371*-GAL4. The red dashed lines highlight the midline, the dashed boxes indicate one hemisegment. Within each hemisegment, RFP-positive cells were counted to define the average number of MNs present per abdominal hemisegment. *Right panel*: table depicting the average number of MNs per hemisegment counted in stage 16 and late-stage 17 embryos.

B Expression of known motoneuron subtype markers (Landgraf *et al*, 1999; Certel & Thor, 2004; Garces & Thor, 2006; Technau *et al*, 2014; Zarin *et al*, 2014; Couton *et al*, 2015) on $n = 758$ cells from the *twit*^{low} cluster. Columns correspond to single cells. Cells were assigned as dorsally projecting MNs (dMN) or ventrally projecting MNs (vMN) by computing expression scores on dMN and vMN markers. If a given marker was observed in a single cell, $-\log(p)$ was added to the respective score, where p is the total fraction of cells expressing a marker.

C Scatter plot comparing the fraction of MNs falling into the distinct classes according to literature (Zarin *et al*, 2014), and according to the assignment performed in (B).

D Bar chart depicting the expression of the marker genes in the different populations.

E Principal component analysis (PCA) of genes expressed in *twit*^{low} cells. GO term analysis for biological processes was performed on the top 10% genes with highest loadings on principal component 1, PC1 (log 10 *P*-value; left), principal component 2, PC2 (middle) and principal component 3, PC3 (right). GO term and SMART domain analysis was performed on the top 300 genes representing the most enriched candidates in the PCA. Dark grey indicates processes enriched among genes with positive loadings, light grey indicates processes enriched among genes with negative loadings. Together these analyses indicated that PC1 and PC2 are associated with metabolic processes, cellular differentiation and/or technical variation, while PC3 is associated with anterior/posterior patterning and synaptic processes.

F Principal component loadings plots highlighting homeodomain TF genes (red) as well as genes associated with the GO terms MN axon guidance (blue) and synaptic organization (green). Points with label correspond to the highest 5% of loadings.

©2022 The Authors

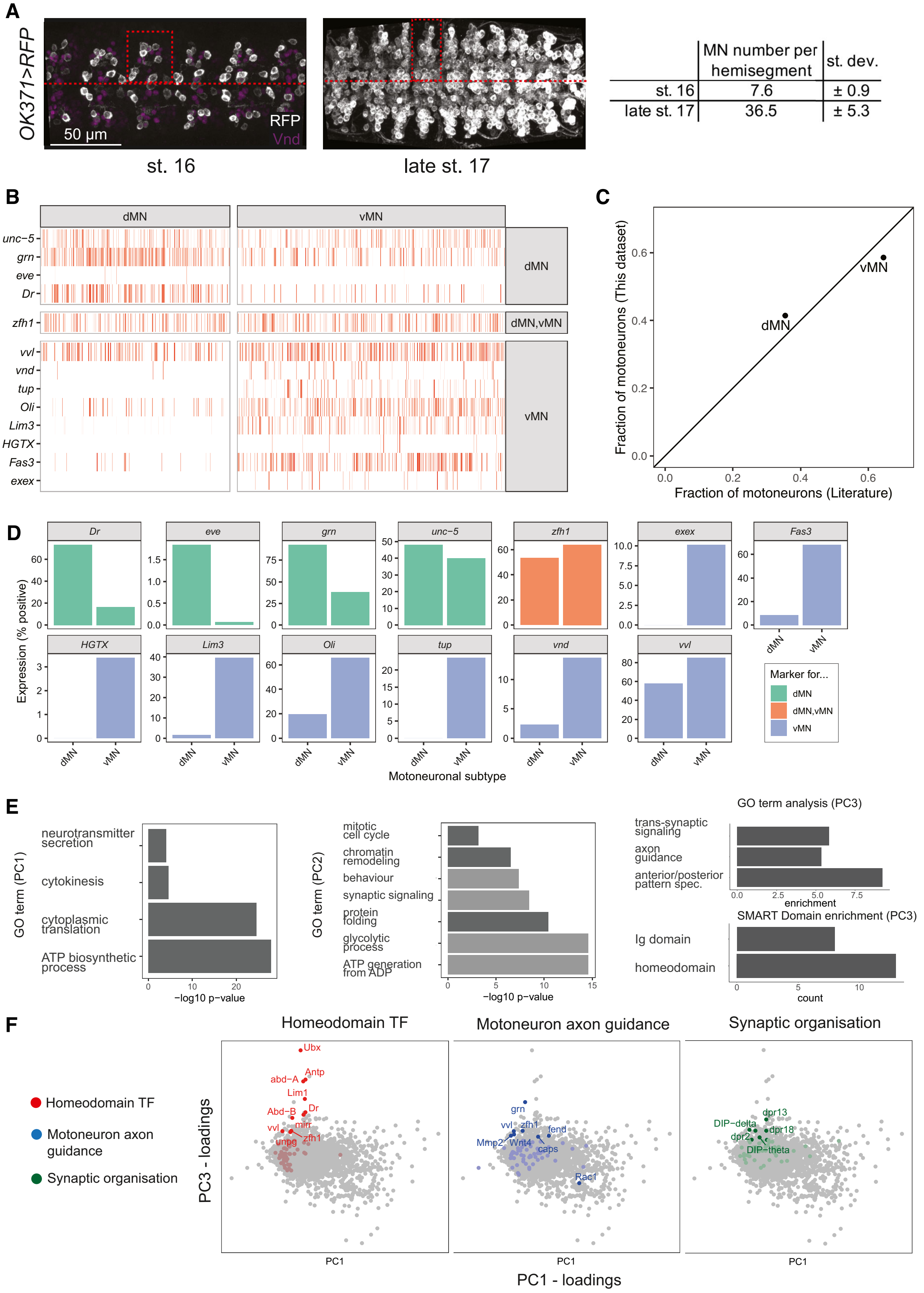

Figure EV3.

 ©2022 The Authors

Figure EV4. The AP axis can be spatially reconstructed based on immunofluorescence measurements of Hox proteins.

A Pipeline for protein intensity measurements of the seven Hox TFs (Lab, Dfd, Scr, Antp, Ubx, AbdA and AbdB) expressed along the AP axis of the ventral nerve cord in a consecutive order. *Upper panel:* The motoneuronal marker FasII (magenta) was used as reference to measure Hox TF expression patterns in different embryos in a standardized manner (see Materials and Methods). *Lower panel:* Procedure of translating different fluorescence intensity measurements into standardized graphs by Fiji.

B Normalized and smoothened protein measurements of Hox TFs (colour code) are combined in one graph (see Materials and Methods). *X*-axis: relative AP position in % (normalized to length of ventral nerve cord), *Y*-axis = relative fluorescence intensity measurement (normalized to max. intensity).

C Normalized and smoothened single-cell mRNA measurements of *Hox* gene expression (colour code) arranged along the inferred AP position (See Fig 1D, Materials and Methods).

D Proof of concept: comparison of expression of two candidate genes (*frq1* and *hth*) on the protein level (solid line) along the measured AP axis and the mRNA expression level (dashed line) along the inferred AP position (see Fig 1D, Materials and Methods), highlighting a high degree of agreement.

E Scatter plots relating principal component scores on PC3 and PC4 to AP position. PCA was performed using only cells from the $twit^{low}$ cluster. PCA was performed including (upper panel) or excluding (lower panel) *Hox* genes to rule out any qualitative biases created by targeted *HoxSeq*. *P*-values were computed based on the hypothesis that the true correlation is different from 0 using a fisher transform of correlation coefficients.

F *Left panel:* The homeodomain TFs Vvl and Mirr identified in ZiNB-WAVE analysis were investigated for the localization along the DV axis of the ventral nerve cord (highlighted in white) of an early-stage 17 *Drosophila* embryo. Here, co-expression of Dfd (magenta) and Mirr (blue) is shown. *Central panel:* Illustration highlighting the maxillary segment (Mx). *Right panel:* Zoom on the expression of Vvl and Mirr in the Dfd expressing maxillary segment (Mx; magenta) is shown, highlighting that Vvl and Mirr are expressed in ventral regions.

G Normalization strategy of scRNA-Seq data analysis modified for sparse and lowly expressed genes (see Materials and Methods). For gene “Low”, expression takes 0 or small values from 1 to 10 counts, for gene “High”, expression can be 0, low (1–10), or high (30-100). After normalization, quantitative differences between cells expressing gene “Low” are effectively voided, whereas they are preserved for gene “High”. *Left panel:* Histogram of raw data, *right panel*: Density plot after normalization.

H Scatter plot depicting for each homeo-cluster the strength of association with a technical covariate (sequencing depth). *P*-values were calculated using the Wilcoxon test contrasting sequencing depth in cells from that cluster, and all other cells. The dotted read line indicates the *P*-value required for significance (0.05). All associations are therefore not significant.

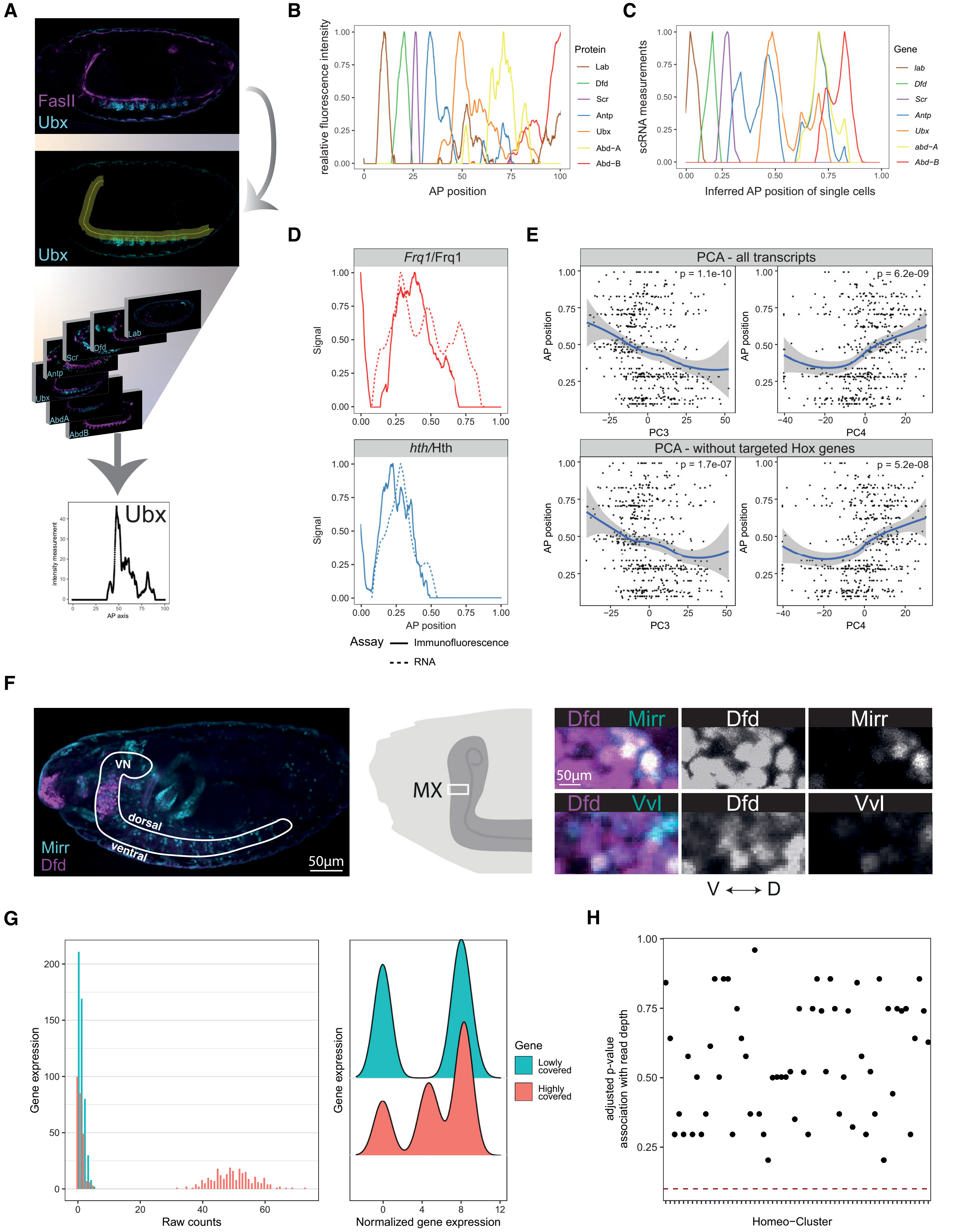

Figure EV4.

©2022 The Authors

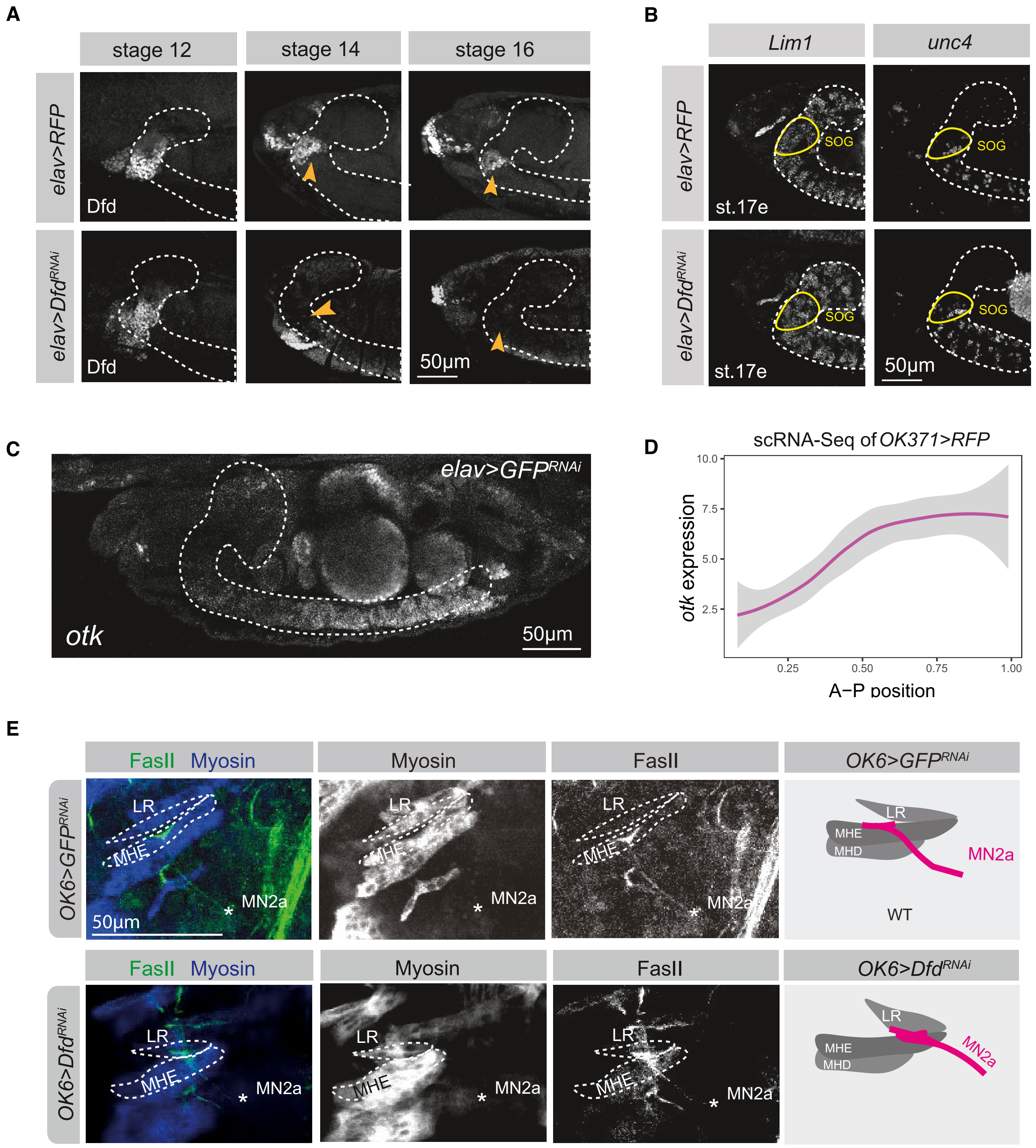
A
stage 12
stage 14
stage 16
elav>RFP
elav>Dfd^RNAi
Dfd
Dfd
50µm
B
Lim1
unc4
elav>RFP
elav>Dfd^RNAi
SOG
st.17e
50µm
C
elav>GFP^RNAi
otk
50µm
D
scRNA-Seq of OK371>RFP
otk expression
A−P position
10.0
7.5
5.0
2.5
0.25
0.50
0.75
1.00
E
FasII Myosin
Myosin
FasII
OK6>GFP^RNAi
OK6>Dfd^RNAi
LR
MHE
MHD
MN2a
50µm
WT

Figure EV5.

◀ ?

Figure EV5. Homeodomain TFs control Immunoglobulin gene expression in the embryonic nervous system.

A Representative confocal images of stage 12, 14 and 16 *Drosophila* control (*elav* > *RFP*) and *elav* > *Dfd*^{RNAi} embryos, depicting Dfd expression (red) and the ventral nerve cord, which is outlined by a white dashed line in all embryos (identified by Elav staining). The orange arrowheads highlight the area of neuronal Dfd expression, which is abolished in *elav* > *Dfd*^{RNAi} embryos at stage 14.

B HCR for identifying *Lim1* and *unc4* transcripts in *Drosophila* stage 17 embryos. RNA expression of both genes is analysed in control (*elav* > *RFP*) versus Dfd-depleted (*elav* > *Dfd*^{RNAi}) animals, the ventral nerve cord is highlighted by a white dashed line, the Dfd-expressing subesophageal ganglion (SOG) is indicated by a yellow circle.

C Representative confocal image of a stage 17 *Drosophila* embryo, highlighting expression of Ig gene *otk* along the ventral nerve cord (white dashed line) as detected by HCR.

D Graph depicting *otk* mRNA expression level along the inferred AP position based on the scRNA-Seq data.

E Representative confocal image of a stage 17 embryonic head with Myosin-expressing muscles (blue) and FasII-expressing axonal projections (green) in animals after RNA interference with *Dfd* in MNs by means of the *OK6*-GAL4 driver. A zoom on the projection (FasII staining) of MN2a to the MHE and LR muscles of an early-stage 17 *Drosophila* embryo are shown. Asterisks highlight the location of MN2a, which is identified by the FasII-expressing axonal projection emerging from a Dfd-expressing MN, which normally innervates the Dfd-expressing MHE muscle. The panel on the right side represents a schematic drawing of the confocal image shown on the left side, summarizing the innervation of the anterior muscles (LR, MHE, MHD) by projections emerging from MN2a (magenta) in the perturbation condition.

 ©2022 The Authors
