# Supplementary material for: Single‐cell RNA sequencing of motoneurons identifies regulators of synaptic wiring in Drosophila embryos
Source: Mol Syst Biol. 2022 Feb 28;18(3):e10255. doi: 10.15252/msb.202110255 (PMC8883443; doi:10.15252/msb.202110255)
Supplement: Supplementary file 2 — Dataset EV1 [file MSB-18-e10255-s003.docx]

**Homeodomain transcription factor gene expression in scRNA-Seq identified clusters:**

| AP | Gene | Mean |
| --- | --- | --- |
| 1 | zfh2 | 12.17 |
| 1 | Lim1 | 17.22 |
| 1 | NK7.1 | 12.66 |
| 1 | lab | 13.97 |
| 1 | exd | 11.31 |
| 1 | hth | 8.70 |
| 2 | dve | 18.22 |
| 2 | ct | 14.85 |
| 2 | Dr | 10.86 |
| 2 | oc | 14.17 |
| 2 | hth | 11.49 |
| 2 | ap | 9.52 |
| 2 | lab | 12.16 |
| 3 | zfh2 | 12.02 |
| 3 | hth | 15.78 |
| 3 | lab | 11.35 |
| 3 | exd | 7.56 |
| 4 | mirr | 17.70 |
| 4 | vvl | 17.57 |
| 4 | zfh2 | 15.45 |
| 4 | Lmx1a | 16.88 |
| 4 | hth | 13.17 |
| 4 | hbn | 11.11 |
| 4 | toy | 12.53 |
| 4 | Rx | 13.93 |
| 4 | exd | 9.95 |
| 4 | lab | 8.72 |
| 5 | pb | 14.47 |
| 5 | Hmx | 15.98 |
| 5 | hth | 17.08 |
| 5 | mirr | 16.67 |
| 5 | otp | 13.70 |
| 5 | tup | 8.61 |
| 5 | lab | 9.77 |
| 5 | eyg | 6.50 |
| 6 | hth | 18.08 |
| 6 | mirr | 14.61 |
| 6 | ro | 9.06 |
| 6 | exd | 14.36 |
| 6 | oc | 8.66 |
| 6 | lab | 9.92 |
| 6 | eyg | 10.98 |
| 6 | Hmx | 7.39 |
| 7 | zfh2 | 11.29 |
| 7 | otp | 14.62 |
| 7 | toy | 10.70 |
| 7 | NK7.1 | 15.88 |
| 7 | exd | 11.64 |
| 7 | eyg | 7.93 |
| 7 | lab | 6.58 |
| 8 | zfh2 | 17.59 |
| 8 | mirr | 11.11 |
| 8 | Lmx1a | 16.85 |
| 8 | CG4328 | 15.27 |
| 8 | hth | 11.02 |
| 8 | vvl | 10.19 |
| 8 | NK7.1 | 11.42 |
| 8 | lab | 6.76 |
| 8 | exd | 7.16 |
| 8 | Rx | 6.03 |
| 9 | hth | 19.04 |
| 9 | Lim3 | 17.35 |
| 9 | Ptx1 | 15.54 |
| 9 | Drgx | 11.36 |
| 9 | exd | 8.41 |
| 9 | vvl | 7.47 |
| 9 | lab | 9.90 |
| 10 | zfh2 | 17.52 |
| 10 | Lim1 | 17.59 |
| 10 | hth | 14.37 |
| 10 | toy | 11.07 |
| 10 | lab | 11.09 |
| 10 | NK7.1 | 7.76 |
| 11 | hth | 18.28 |
| 11 | toy | 16.05 |
| 11 | Lim1 | 16.19 |
| 11 | zfh2 | 16.02 |
| 11 | vvl | 14.88 |
| 11 | unc-4 | 14.76 |
| 11 | Dfd | 10.85 |
| 11 | exd | 11.20 |
| 11 | Dr | 9.21 |
| 11 | nub | 7.41 |
| 11 | eyg | 4.88 |
| 12 | hth | 18.95 |
| 12 | Lim1 | 16.87 |
| 12 | exd | 9.62 |
| 12 | Dr | 8.10 |
| 12 | Dfd | 6.43 |
| 12 | zfh2 | 6.27 |
| 13 | hth | 17.86 |
| 13 | mirr | 17.39 |
| 13 | zfh2 | 17.39 |
| 13 | pb | 15.90 |
| 13 | Dfd | 16.20 |
| 13 | exex | 15.68 |
| 13 | caup | 15.24 |
| 13 | vvl | 14.17 |
| 13 | exd | 13.85 |
| 13 | ara | 12.44 |
| 13 | Lim3 | 8.99 |
| 13 | lms | 5.81 |
| 13 | eyg | 4.53 |
| 14 | mirr | 17.64 |
| 14 | zfh2 | 17.39 |
| 14 | vvl | 16.93 |
| 14 | Dfd | 13.22 |
| 14 | Ptx1 | 8.86 |
| 14 | hth | 11.22 |
| 14 | Dr | 8.89 |
| 14 | NK7.1 | 9.19 |
| 15 | mirr | 17.62 |
| 15 | hth | 17.26 |
| 15 | vvl | 16.92 |
| 15 | zfh2 | 14.04 |
| 15 | nub | 14.95 |
| 15 | Dfd | 16.03 |
| 15 | B-H1 | 13.49 |
| 15 | tup | 12.37 |
| 15 | pdm2 | 10.35 |
| 15 | B-H2 | 13.96 |
| 15 | exd | 8.93 |
| 15 | NK7.1 | 6.57 |
| 16 | Lim1 | 17.89 |
| 16 | Drgx | 17.68 |
| 16 | exd | 12.36 |
| 16 | ct | 9.18 |
| 16 | toy | 8.55 |
| 16 | hth | 7.14 |
| 16 | lab | 6.55 |
| 16 | pros | 5.61 |
| 17 | dve | 18.04 |
| 17 | Ptx1 | 16.97 |
| 17 | tup | 12.09 |
| 17 | exd | 13.40 |
| 17 | eyg | 8.75 |
| 17 | vvl | 8.94 |
| 17 | hth | 7.46 |
| 17 | lab | 5.74 |
| 17 | NK7.1 | 5.20 |
| 17 | Vsx1 | 4.87 |
| 17 | Ubx | 4.87 |
| 18 | hth | 18.05 |
| 18 | mirr | 17.83 |
| 18 | zfh2 | 17.12 |
| 18 | Lim1 | 16.16 |
| 18 | unc-4 | 13.18 |
| 18 | toy | 14.53 |
| 18 | NK7.1 | 8.91 |
| 18 | slou | 8.44 |
| 18 | Dfd | 10.80 |
| 18 | exd | 6.79 |
| 19 | hth | 18.36 |
| 19 | vvl | 16.81 |
| 19 | mirr | 15.11 |
| 19 | Scr | 14.74 |
| 19 | zfh2 | 10.74 |
| 19 | Lim1 | 9.53 |
| 20 | hth | 18.38 |
| 20 | Lim1 | 16.99 |
| 20 | ct | 9.98 |
| 20 | Dr | 7.24 |
| 20 | Scr | 7.14 |
| 21 | hth | 17.36 |
| 21 | mirr | 17.38 |
| 21 | zfh2 | 17.49 |
| 21 | Scr | 16.33 |
| 21 | Lim3 | 13.33 |
| 21 | ara | 9.78 |
| 21 | caup | 15.67 |
| 21 | vvl | 14.08 |
| 21 | eyg | 7.49 |
| 22 | hth | 16.37 |
| 22 | ct | 16.70 |
| 22 | zfh2 | 17.00 |
| 22 | vvl | 12.54 |
| 22 | Scr | 13.64 |
| 23 | hth | 18.18 |
| 23 | zfh2 | 15.83 |
| 23 | Lim3 | 15.70 |
| 23 | lab | 8.54 |
| 23 | Scr | 7.62 |
| 23 | NK7.1 | 7.76 |
| 24 | nub | 16.62 |
| 24 | zfh2 | 17.53 |
| 24 | pdm2 | 14.50 |
| 24 | toy | 13.52 |
| 24 | exd | 10.78 |
| 24 | hth | 11.78 |
| 24 | Lim3 | 7.31 |
| 24 | oc | 7.74 |
| 24 | lms | 6.03 |
| 24 | NK7.1 | 6.64 |
| 25 | dve | 18.20 |
| 25 | zfh2 | 14.79 |
| 25 | oc | 14.71 |
| 25 | exd | 12.53 |
| 25 | hth | 9.69 |
| 25 | Lim3 | 8.13 |
| 25 | ap | 7.39 |
| 25 | NK7.1 | 7.19 |
| 25 | Antp | 5.47 |
| 26 | CG32532 | 16.36 |
| 26 | vvl | 17.09 |
| 26 | NK7.1 | 13.39 |
| 26 | B-H1 | 14.35 |
| 26 | toy | 12.88 |
| 26 | zfh2 | 10.91 |
| 26 | hth | 7.95 |
| 26 | Antp | 7.06 |
| 26 | B-H2 | 6.87 |
| 26 | exd | 5.61 |
| 27 | hth | 17.49 |
| 27 | zfh2 | 15.71 |
| 27 | vvl | 12.10 |
| 27 | Lim1 | 12.51 |
| 27 | Antp | 13.29 |
| 27 | exd | 8.21 |
| 28 | hth | 18.19 |
| 28 | Antp | 16.08 |
| 28 | mirr | 16.33 |
| 28 | zfh2 | 16.39 |
| 28 | Lim1 | 13.02 |
| 28 | ct | 14.94 |
| 28 | vvl | 13.89 |
| 28 | NK7.1 | 14.34 |
| 28 | oc | 13.65 |
| 28 | exd | 8.55 |
| 28 | Lim3 | 6.52 |
| 28 | dve | 8.27 |
| 29 | hth | 15.10 |
| 29 | Lim3 | 13.90 |
| 29 | exd | 10.80 |
| 29 | Abd-B | 7.61 |
| 29 | ct | 7.00 |
| 30 | hth | 16.77 |
| 30 | mirr | 12.86 |
| 30 | zfh2 | 14.41 |
| 30 | vvl | 15.96 |
| 30 | Lim1 | 15.82 |
| 30 | dve | 14.03 |
| 30 | oc | 11.37 |
| 30 | Ubx | 8.88 |
| 30 | Antp | 8.28 |
| 30 | exd | 7.65 |
| 30 | lab | 5.45 |
| 31 | Antp | 13.60 |
| 31 | zfh2 | 16.84 |
| 31 | NK7.1 | 14.37 |
| 31 | hth | 8.45 |
| 32 | hth | 17.84 |
| 32 | zfh2 | 16.75 |
| 32 | otp | 16.34 |
| 32 | vvl | 9.28 |
| 32 | Antp | 15.06 |
| 32 | Lmx1a | 11.37 |
| 32 | exd | 8.00 |
| 33 | Antp | 13.88 |
| 33 | unpg | 15.08 |
| 33 | mirr | 15.25 |
| 33 | Ubx | 13.34 |
| 33 | zfh2 | 16.18 |
| 33 | hth | 13.84 |
| 33 | vvl | 14.04 |
| 33 | abd-A | 8.34 |
| 33 | Abd-B | 8.46 |
| 34 | Ubx | 17.52 |
| 34 | zfh2 | 17.70 |
| 34 | Dr | 13.99 |
| 34 | Lim1 | 15.71 |
| 34 | Antp | 15.81 |
| 34 | hth | 10.78 |
| 34 | exd | 10.21 |
| 34 | ey | 9.43 |
| 35 | Antp | 17.20 |
| 35 | Ubx | 13.68 |
| 35 | mirr | 17.05 |
| 35 | vvl | 16.30 |
| 35 | zfh2 | 13.05 |
| 35 | ara | 12.13 |
| 35 | exex | 11.94 |
| 35 | Lim3 | 13.32 |
| 35 | exd | 8.90 |
| 35 | lab | 7.25 |
| 35 | caup | 7.44 |
| 36 | Ubx | 16.74 |
| 36 | mirr | 17.09 |
| 36 | Dr | 13.85 |
| 36 | zfh2 | 12.58 |
| 36 | Lim1 | 13.22 |
| 36 | vvl | 12.92 |
| 36 | hth | 10.43 |
| 36 | Antp | 8.24 |
| 36 | exd | 9.35 |
| 37 | Lim1 | 16.83 |
| 37 | Ubx | 14.35 |
| 37 | ct | 11.28 |
| 37 | abd-A | 14.95 |
| 37 | mirr | 13.60 |
| 38 | zfh2 | 17.37 |
| 38 | Ubx | 16.57 |
| 38 | mirr | 16.47 |
| 38 | Antp | 12.94 |
| 38 | Lim1 | 14.20 |
| 38 | hth | 11.72 |
| 38 | oc | 11.89 |
| 38 | vvl | 10.64 |
| 38 | abd-A | 11.55 |
| 39 | dve | 18.13 |
| 39 | hth | 16.54 |
| 39 | ct | 14.53 |
| 39 | Ubx | 11.08 |
| 39 | Antp | 14.15 |
| 39 | Lim1 | 13.89 |
| 39 | oc | 13.52 |
| 39 | NK7.1 | 9.78 |
| 39 | exd | 8.71 |
| 39 | nub | 8.01 |
| 39 | abd-A | 8.31 |
| 40 | zfh2 | 16.66 |
| 40 | Ubx | 16.01 |
| 40 | Lim1 | 16.58 |
| 40 | hth | 12.43 |
| 40 | Abd-B | 10.01 |
| 41 | hth | 16.34 |
| 41 | Antp | 15.14 |
| 41 | Lim1 | 16.62 |
| 41 | Ubx | 12.95 |
| 41 | Dr | 15.32 |
| 41 | ey | 15.11 |
| 41 | vnd | 12.79 |
| 41 | abd-A | 9.13 |
| 41 | exd | 7.93 |
| 42 | pb | 18.11 |
| 42 | unpg | 18.09 |
| 42 | Antp | 16.98 |
| 42 | Lim3 | 16.03 |
| 42 | abd-A | 15.50 |
| 42 | zfh2 | 15.19 |
| 42 | Ubx | 14.69 |
| 42 | ct | 14.60 |
| 42 | Vsx1 | 11.93 |
| 42 | lab | 10.90 |
| 42 | Abd-B | 7.80 |
| 42 | dve | 5.66 |
| 42 | lms | 3.83 |
| 42 | pros | 3.17 |
| 43 | Ubx | 17.48 |
| 43 | mirr | 17.17 |
| 43 | ct | 14.73 |
| 43 | Lim1 | 14.80 |
| 43 | vvl | 14.93 |
| 43 | abd-A | 12.64 |
| 43 | Antp | 14.41 |
| 43 | oc | 13.39 |
| 43 | NK7.1 | 7.84 |
| 43 | exd | 7.83 |
| 43 | hth | 7.81 |
| 44 | zfh2 | 14.90 |
| 44 | Ubx | 13.42 |
| 44 | abd-A | 13.70 |
| 44 | hth | 13.19 |
| 44 | Antp | 12.39 |
| 45 | hth | 17.41 |
| 45 | zfh2 | 15.59 |
| 45 | Ubx | 16.68 |
| 45 | Lim1 | 16.32 |
| 45 | abd-A | 16.09 |
| 45 | Antp | 11.72 |
| 45 | oc | 8.38 |
| 46 | dve | 17.26 |
| 46 | mirr | 15.73 |
| 46 | Ubx | 17.03 |
| 46 | abd-A | 13.34 |
| 46 | Antp | 13.55 |
| 46 | zfh2 | 12.87 |
| 46 | Lim1 | 15.90 |
| 46 | ct | 13.72 |
| 46 | oc | 14.19 |
| 46 | NK7.1 | 10.63 |
| 46 | hth | 7.04 |
| 47 | mirr | 15.64 |
| 47 | Ubx | 15.70 |
| 47 | hth | 16.26 |
| 47 | abd-A | 15.24 |
| 47 | Lim1 | 12.22 |
| 47 | ct | 11.05 |
| 47 | Antp | 9.17 |
| 47 | exd | 7.58 |
| 47 | NK7.1 | 7.02 |
| 47 | vvl | 7.07 |
| 48 | pb | 18.20 |
| 48 | Ubx | 16.86 |
| 48 | Lim1 | 15.52 |
| 48 | Dr | 15.25 |
| 48 | abd-A | 12.33 |
| 48 | zfh2 | 16.19 |
| 48 | hth | 10.22 |
| 48 | ey | 12.86 |
| 48 | NK7.1 | 9.11 |
| 48 | exd | 9.25 |
| 48 | eyg | 4.52 |
| 49 | vvl | 17.65 |
| 49 | ap | 16.88 |
| 49 | zfh2 | 15.92 |
| 49 | slou | 15.77 |
| 49 | abd-A | 10.67 |
| 49 | exd | 14.49 |
| 49 | Ubx | 13.51 |
| 49 | nub | 8.93 |
| 50 | Ubx | 16.65 |
| 50 | Antp | 15.46 |
| 50 | zfh2 | 15.86 |
| 50 | abd-A | 16.43 |
| 50 | Lim1 | 16.10 |
| 50 | ey | 13.45 |
| 50 | Dr | 12.76 |
| 50 | Abd-B | 8.28 |
| 50 | vnd | 7.80 |
| 50 | hth | 7.32 |
| 51 | Ubx | 14.99 |
| 51 | toy | 17.11 |
| 51 | zfh2 | 15.73 |
| 51 | abd-A | 12.18 |
| 51 | Dr | 15.82 |
| 51 | Antp | 15.38 |
| 51 | vvl | 12.15 |
| 51 | hth | 11.17 |
| 51 | Lim1 | 11.91 |
| 52 | zfh2 | 17.98 |
| 52 | abd-A | 14.32 |
| 52 | slou | 16.13 |
| 52 | Lim1 | 16.66 |
| 52 | hth | 15.25 |
| 52 | unc-4 | 16.00 |
| 52 | Ubx | 10.53 |
| 52 | Antp | 8.46 |
| 52 | lab | 4.94 |
| 53 | abd-A | 16.90 |
| 53 | vvl | 14.59 |
| 53 | Ptx1 | 16.25 |
| 53 | Abd-B | 11.46 |
| 53 | Lim3 | 13.57 |
| 53 | NK7.1 | 10.89 |
| 53 | hth | 7.14 |
| 53 | exd | 6.11 |
| 54 | mirr | 15.76 |
| 54 | ct | 16.77 |
| 54 | Ubx | 16.42 |
| 54 | Antp | 12.92 |
| 54 | Abd-B | 15.88 |
| 54 | abd-A | 13.16 |
| 54 | vvl | 11.49 |
| 54 | Lim1 | 14.39 |
| 54 | oc | 14.58 |
| 54 | hth | 9.69 |
| 54 | NK7.1 | 7.61 |
| 55 | Abd-B | 16.01 |
| 55 | Lim1 | 17.04 |
| 55 | zfh2 | 16.52 |
| 55 | Dr | 15.13 |
| 55 | abd-A | 13.51 |
| 55 | ey | 11.54 |
| 55 | pb | 8.60 |
| 56 | abd-A | 18.00 |
| 56 | Abd-B | 14.21 |
| 56 | Lim1 | 15.77 |
| 56 | Dr | 9.96 |
| 56 | Antp | 9.34 |
| 56 | hth | 7.17 |
| 56 | vvl | 7.22 |
| 57 | mirr | 14.88 |
| 57 | zfh2 | 16.47 |
| 57 | Lim1 | 16.72 |
| 57 | abd-A | 15.98 |
| 57 | Abd-B | 12.54 |
| 57 | slou | 13.69 |
| 57 | Ptx1 | 13.48 |
| 57 | toy | 11.21 |
| 57 | unc-4 | 14.47 |
| 57 | Antp | 7.53 |
| 57 | eyg | 7.74 |
| 57 | hth | 9.17 |
| 58 | vvl | 16.82 |
| 58 | Dr | 16.87 |
| 58 | Ubx | 12.35 |
| 58 | zfh2 | 16.87 |
| 58 | mirr | 16.85 |
| 58 | Abd-B | 15.89 |
| 58 | Lim1 | 16.07 |
| 58 | abd-A | 15.46 |
| 58 | Antp | 14.29 |
| 59 | zfh2 | 17.40 |
| 59 | Abd-B | 16.39 |
| 59 | mirr | 14.35 |
| 59 | abd-A | 13.10 |
| 59 | Lim3 | 14.19 |
| 59 | vvl | 10.18 |
| 59 | hth | 13.86 |
| 59 | NK7.1 | 8.44 |
| 60 | zfh2 | 18.26 |
| 60 | Abd-B | 16.40 |
| 60 | vvl | 14.52 |
| 60 | Lim1 | 11.39 |
| 60 | oc | 11.01 |
| 60 | abd-A | 11.30 |

**Immunglobulin gene expression in scRNA-Seq identified clusters:**

| AP | Gene | mean |
| --- | --- | --- |
| 1 | CG17716 | 12.50 |
| 1 | Dscam2 | 14.19 |
| 1 | Ptp99A | 12.80 |
| 1 | klg | 9.08 |
| 1 | kek2 | 11.61 |
| 1 | CG42313 | 10.56 |
| 1 | CG34114 | 8.90 |
| 1 | dpr1 | 8.10 |
| 1 | dpr6 | 7.72 |
| 1 | beat-VII | 8.53 |
| 1 | beat-IIa | 10.27 |
| 1 | dpr8 | 11.72 |
| 1 | kek1 | 8.78 |
| 1 | Fas2 | 10.70 |
| 1 | side | 8.59 |
| 1 | hbs | 10.51 |
| 1 | CG34371 | 10.13 |
| 1 | dpr11 | 7.53 |
| 1 | ed | 8.01 |
| 1 | dpr9 | 7.73 |
| 1 | dpr2 | 7.51 |
| 1 | Dscam3 | 8.71 |
| 1 | fred | 7.04 |
| 1 | kirre | 6.14 |
| 1 | Dscam4 | 6.63 |
| 2 | kek2 | 11.18 |
| 2 | side | 12.29 |
| 2 | Ptp99A | 15.10 |
| 2 | beat-IIa | 14.48 |
| 2 | kek1 | 11.07 |
| 2 | dpr8 | 10.84 |
| 2 | robo3 | 12.08 |
| 2 | klg | 9.08 |
| 2 | CG34353 | 12.02 |
| 2 | CG17716 | 10.52 |
| 2 | dpr13 | 10.09 |
| 2 | beat-VI | 10.66 |
| 2 | Dscam4 | 13.25 |
| 2 | CG34114 | 8.39 |
| 2 | dpr1 | 8.70 |
| 2 | kirre | 8.01 |
| 2 | beat-VII | 7.57 |
| 2 | Dscam2 | 7.49 |
| 2 | dpr6 | 6.32 |
| 2 | robo2 | 7.34 |
| 2 | nolo | 5.64 |
| 2 | dpr9 | 6.01 |
| 2 | dpr18 | 5.65 |
| 3 | Ptp99A | 13.63 |
| 3 | side | 12.26 |
| 3 | kek1 | 11.10 |
| 3 | dpr13 | 8.74 |
| 3 | Dscam2 | 8.99 |
| 3 | CG17716 | 9.12 |
| 3 | kek2 | 8.49 |
| 3 | Dscam4 | 8.58 |
| 3 | dpr9 | 8.32 |
| 3 | beat-IIa | 8.00 |
| 3 | kirre | 8.85 |
| 3 | Dscam3 | 8.02 |
| 3 | klg | 7.55 |
| 3 | dpr8 | 7.85 |
| 3 | hbs | 6.30 |
| 3 | CG12484 | 6.90 |
| 3 | CG34371 | 6.51 |
| 3 | kek3 | 6.25 |
| 3 | fred | 6.41 |
| 4 | beat-VI | 14.86 |
| 4 | Ptp99A | 15.57 |
| 4 | beat-IIa | 12.34 |
| 4 | CG17716 | 10.50 |
| 4 | kek1 | 14.99 |
| 4 | dpr1 | 10.80 |
| 4 | beat-IIIb | 8.86 |
| 4 | Dscam4 | 12.28 |
| 4 | side | 9.61 |
| 4 | Fas2 | 8.27 |
| 4 | kek2 | 13.33 |
| 4 | dpr9 | 11.42 |
| 4 | CG31814 | 8.24 |
| 4 | beat-VII | 9.63 |
| 4 | CG34353 | 8.92 |
| 4 | dpr17 | 7.25 |
| 4 | kirre | 6.80 |
| 4 | dpr8 | 7.68 |
| 4 | nolo | 6.58 |
| 4 | kek3 | 5.73 |
| 4 | CG12484 | 5.45 |
| 4 | CG34114 | 7.24 |
| 5 | klg | 13.66 |
| 5 | Dscam2 | 12.99 |
| 5 | CG42313 | 12.42 |
| 5 | side | 11.95 |
| 5 | kek1 | 9.13 |
| 5 | CG17716 | 11.95 |
| 5 | Dscam4 | 13.55 |
| 5 | Ptp99A | 11.61 |
| 5 | CG34114 | 8.41 |
| 5 | beat-IIa | 10.62 |
| 5 | ed | 10.96 |
| 5 | beat-VI | 8.30 |
| 5 | hig | 9.93 |
| 5 | nolo | 7.65 |
| 5 | dpr17 | 8.24 |
| 5 | dpr20 | 7.97 |
| 5 | kek3 | 8.29 |
| 5 | dpr13 | 8.81 |
| 5 | dpr3 | 7.71 |
| 5 | dpr2 | 6.80 |
| 5 | dpr9 | 7.28 |
| 6 | side | 12.65 |
| 6 | CG12484 | 15.46 |
| 6 | kek2 | 11.64 |
| 6 | dpr13 | 11.75 |
| 6 | kek1 | 10.63 |
| 6 | Ptp99A | 10.92 |
| 6 | CG17716 | 12.83 |
| 6 | dpr9 | 12.37 |
| 6 | dpr8 | 8.99 |
| 6 | Dscam4 | 12.90 |
| 6 | CG34371 | 11.16 |
| 6 | beat-Ib | 7.98 |
| 6 | klg | 8.92 |
| 6 | nolo | 7.59 |
| 6 | dpr3 | 7.45 |
| 6 | kek3 | 6.75 |
| 6 | beat-IIa | 6.44 |
| 6 | dpr6 | 7.31 |
| 7 | CG12484 | 15.32 |
| 7 | Ptp99A | 15.98 |
| 7 | kek1 | 11.80 |
| 7 | kek2 | 10.17 |
| 7 | Dscam2 | 8.75 |
| 7 | Fas2 | 10.41 |
| 7 | dpr9 | 14.05 |
| 7 | Dscam3 | 10.72 |
| 7 | kirre | 11.07 |
| 7 | Dscam4 | 11.90 |
| 7 | dpr18 | 9.82 |
| 7 | side | 10.29 |
| 7 | dpr8 | 12.40 |
| 7 | robo3 | 7.80 |
| 7 | dpr17 | 7.42 |
| 7 | kek5 | 9.69 |
| 7 | klg | 8.81 |
| 7 | CG34114 | 7.19 |
| 7 | beat-Ib | 7.50 |
| 7 | ed | 7.56 |
| 7 | dpr13 | 6.80 |
| 8 | side | 13.15 |
| 8 | CG17716 | 12.90 |
| 8 | kek1 | 12.50 |
| 8 | dpr9 | 12.83 |
| 8 | beat-VI | 11.12 |
| 8 | CG12484 | 9.37 |
| 8 | Ptp99A | 12.65 |
| 8 | Dscam4 | 10.80 |
| 8 | kek2 | 10.64 |
| 8 | dpr8 | 10.04 |
| 8 | kek3 | 8.85 |
| 8 | kek5 | 9.02 |
| 8 | kirre | 11.50 |
| 8 | ed | 7.55 |
| 8 | dpr1 | 7.67 |
| 8 | CG42313 | 7.40 |
| 8 | hig | 7.04 |
| 8 | hbs | 6.62 |
| 8 | beat-Ic | 7.58 |
| 8 | fred | 6.58 |
| 8 | beat-VII | 6.12 |
| 8 | nolo | 6.91 |
| 8 | CG34114 | 6.89 |
| 8 | beat-IIa | 6.40 |
| 8 | dpr17 | 5.26 |
| 8 | dpr18 | 5.84 |
| 9 | DIP-beta | 13.13 |
| 9 | Fas2 | 14.45 |
| 9 | beat-Ic | 12.41 |
| 9 | kek2 | 14.95 |
| 9 | dpr13 | 15.00 |
| 9 | dpr9 | 11.62 |
| 9 | CG17716 | 14.39 |
| 9 | Dscam2 | 11.92 |
| 9 | dpr6 | 14.55 |
| 9 | ed | 11.81 |
| 9 | CG12484 | 11.11 |
| 9 | nolo | 11.58 |
| 9 | Ptp99A | 13.92 |
| 9 | CG14372 | 13.44 |
| 9 | dpr8 | 10.72 |
| 9 | Dscam3 | 10.68 |
| 9 | dpr1 | 8.67 |
| 9 | Dscam4 | 13.82 |
| 9 | kirre | 12.67 |
| 9 | CG42313 | 10.81 |
| 9 | beat-Ib | 7.96 |
| 9 | beat-Va | 9.62 |
| 9 | fred | 9.50 |
| 9 | dpr2 | 7.83 |
| 9 | DIP-theta | 7.88 |
| 9 | beat-Vc | 8.13 |
| 9 | dpr11 | 6.22 |
| 9 | beat-IIa | 6.95 |
| 9 | dpr20 | 6.24 |
| 10 | Ptp99A | 15.74 |
| 10 | CG42313 | 10.06 |
| 10 | kek2 | 11.43 |
| 10 | CG34114 | 10.92 |
| 10 | dpr9 | 9.46 |
| 10 | dpr8 | 8.02 |
| 10 | beat-IIIb | 9.08 |
| 10 | Dscam2 | 9.45 |
| 10 | kek1 | 7.92 |
| 10 | Dscam3 | 10.91 |
| 10 | side | 8.87 |
| 10 | beat-VII | 9.54 |
| 10 | dpr10 | 8.88 |
| 10 | dpr1 | 8.05 |
| 10 | kirre | 11.29 |
| 10 | fred | 7.40 |
| 10 | dpr13 | 8.06 |
| 10 | CG17716 | 8.90 |
| 10 | CG34353 | 7.48 |
| 10 | dpr6 | 7.83 |
| 10 | beat-VI | 8.05 |
| 10 | Dscam4 | 8.67 |
| 10 | klg | 7.36 |
| 11 | Ptp99A | 15.36 |
| 11 | dpr8 | 10.08 |
| 11 | CG17716 | 13.08 |
| 11 | kek2 | 12.09 |
| 11 | dpr13 | 10.62 |
| 11 | kek1 | 11.13 |
| 11 | robo3 | 10.52 |
| 11 | CG12484 | 9.63 |
| 11 | klg | 9.36 |
| 11 | dpr6 | 9.39 |
| 11 | beat-IIa | 8.65 |
| 11 | CG34371 | 8.54 |
| 11 | robo2 | 9.57 |
| 11 | Dscam2 | 8.68 |
| 11 | beat-VI | 9.10 |
| 11 | kek3 | 7.37 |
| 11 | dpr9 | 10.80 |
| 11 | beat-IV | 8.16 |
| 11 | kirre | 7.42 |
| 11 | CG34114 | 7.62 |
| 11 | beat-VII | 7.36 |
| 11 | DIP-gamma | 7.46 |
| 11 | dpr11 | 7.49 |
| 11 | side | 7.48 |
| 11 | ed | 6.41 |
| 11 | dpr10 | 6.11 |
| 11 | otk | 6.00 |
| 11 | fred | 6.88 |
| 11 | hbs | 5.35 |
| 12 | Ptp99A | 14.51 |
| 12 | Dscam2 | 13.14 |
| 12 | dpr6 | 9.10 |
| 12 | kek1 | 11.18 |
| 12 | beat-Ic | 9.54 |
| 12 | CG17716 | 9.02 |
| 12 | klg | 9.58 |
| 12 | CG42313 | 8.66 |
| 12 | CG12484 | 9.08 |
| 12 | CG14372 | 9.69 |
| 12 | beat-IIa | 8.57 |
| 12 | dpr10 | 8.15 |
| 12 | Dscam4 | 7.25 |
| 12 | side | 8.47 |
| 12 | dpr11 | 7.55 |
| 12 | dpr1 | 8.28 |
| 12 | robo3 | 9.22 |
| 12 | dpr9 | 7.19 |
| 12 | ed | 7.63 |
| 12 | CG31814 | 7.61 |
| 12 | kek2 | 7.86 |
| 12 | kek3 | 7.17 |
| 12 | hbs | 8.77 |
| 12 | dpr8 | 7.76 |
| 12 | CG34371 | 7.04 |
| 12 | robo2 | 7.08 |
| 12 | Dscam3 | 7.18 |
| 12 | dpr13 | 7.22 |
| 12 | beat-Vc | 6.67 |
| 13 | robo2 | 10.86 |
| 13 | beat-Ic | 13.19 |
| 13 | Ptp99A | 15.80 |
| 13 | side | 15.27 |
| 13 | CG17716 | 14.82 |
| 13 | klg | 14.65 |
| 13 | robo3 | 12.36 |
| 13 | kek1 | 14.26 |
| 13 | beat-IIIc | 11.49 |
| 13 | CG42313 | 12.85 |
| 13 | beat-IIa | 12.79 |
| 13 | kek5 | 13.59 |
| 13 | dpr3 | 13.04 |
| 13 | kek3 | 12.77 |
| 13 | Fas2 | 9.32 |
| 13 | beat-IV | 8.99 |
| 13 | beat-Ia | 9.22 |
| 13 | dpr13 | 9.68 |
| 13 | dpr10 | 10.44 |
| 13 | kek2 | 8.61 |
| 13 | otk | 8.53 |
| 13 | beat-IIIa | 8.27 |
| 13 | CG14372 | 8.53 |
| 13 | dpr2 | 8.20 |
| 13 | CG34353 | 7.88 |
| 13 | Dscam2 | 7.13 |
| 13 | dpr6 | 6.93 |
| 13 | dpr9 | 6.81 |
| 13 | nolo | 5.75 |
| 13 | Dscam4 | 5.73 |
| 13 | dpr17 | 4.66 |
| 13 | hbs | 4.92 |
| 14 | kek1 | 13.18 |
| 14 | dpr8 | 12.47 |
| 14 | beat-VI | 13.81 |
| 14 | CG17716 | 8.31 |
| 14 | Ptp99A | 14.13 |
| 14 | kek2 | 14.11 |
| 14 | Dscam2 | 12.26 |
| 14 | CG31814 | 12.09 |
| 14 | fred | 9.66 |
| 14 | dpr13 | 10.34 |
| 14 | dpr9 | 11.80 |
| 14 | Dscam4 | 11.95 |
| 14 | ed | 7.97 |
| 14 | dpr10 | 9.64 |
| 14 | robo2 | 8.91 |
| 14 | robo3 | 9.63 |
| 14 | beat-IIa | 6.97 |
| 14 | CG14372 | 6.36 |
| 14 | kirre | 6.04 |
| 15 | klg | 16.31 |
| 15 | dpr1 | 16.11 |
| 15 | CG17716 | 15.85 |
| 15 | CG12484 | 14.89 |
| 15 | kek2 | 14.88 |
| 15 | beat-Ic | 14.40 |
| 15 | Ptp99A | 12.32 |
| 15 | robo2 | 12.09 |
| 15 | kek1 | 14.18 |
| 15 | dpr13 | 9.56 |
| 15 | beat-VI | 12.16 |
| 15 | robo3 | 9.36 |
| 15 | CG34114 | 13.21 |
| 15 | CG42313 | 9.87 |
| 15 | beat-Ib | 12.69 |
| 15 | beat-IV | 10.96 |
| 15 | DIP-gamma | 10.88 |
| 15 | kirre | 12.43 |
| 15 | dpr10 | 10.43 |
| 15 | otk | 8.89 |
| 15 | dpr8 | 9.71 |
| 15 | dpr18 | 9.34 |
| 15 | nolo | 9.24 |
| 15 | DIP-zeta | 6.75 |
| 15 | beat-VII | 6.49 |
| 15 | CG34371 | 6.77 |
| 15 | side | 6.79 |
| 15 | dpr20 | 5.91 |
| 15 | Dscam3 | 5.40 |
| 15 | beat-IIa | 5.48 |
| 16 | side | 13.07 |
| 16 | CG17716 | 14.89 |
| 16 | Ptp99A | 14.96 |
| 16 | beat-Ic | 12.41 |
| 16 | klg | 13.56 |
| 16 | dpr6 | 14.28 |
| 16 | beat-VI | 10.00 |
| 16 | beat-IIIb | 11.19 |
| 16 | CG34353 | 9.53 |
| 16 | dpr9 | 10.77 |
| 16 | beat-Ib | 10.43 |
| 16 | kek2 | 11.60 |
| 16 | dpr8 | 10.33 |
| 16 | ed | 8.04 |
| 16 | robo3 | 8.84 |
| 16 | beat-VII | 10.74 |
| 16 | Dscam4 | 8.46 |
| 16 | hbs | 6.95 |
| 16 | otk | 6.77 |
| 16 | CG34371 | 7.06 |
| 16 | beat-Vc | 7.42 |
| 16 | fred | 7.11 |
| 16 | dpr3 | 6.43 |
| 16 | sdk | 6.67 |
| 16 | beat-IIa | 6.23 |
| 16 | dpr11 | 6.14 |
| 16 | CG34114 | 6.38 |
| 16 | dpr20 | 6.42 |
| 16 | beat-IIIa | 5.53 |
| 16 | dpr10 | 5.84 |
| 17 | Dscam2 | 16.77 |
| 17 | Ptp99A | 15.67 |
| 17 | kek1 | 15.02 |
| 17 | dpr8 | 14.98 |
| 17 | dpr1 | 15.14 |
| 17 | CG34353 | 14.22 |
| 17 | beat-IIIb | 14.16 |
| 17 | beat-IIIc | 10.93 |
| 17 | Dscam4 | 12.85 |
| 17 | ed | 12.68 |
| 17 | klg | 10.41 |
| 17 | side | 13.67 |
| 17 | fred | 10.09 |
| 17 | dpr6 | 9.90 |
| 17 | CG31814 | 9.73 |
| 17 | hbs | 11.14 |
| 17 | beat-VI | 9.78 |
| 17 | kirre | 8.92 |
| 17 | beat-IIIa | 8.75 |
| 17 | kek3 | 8.79 |
| 17 | kek2 | 8.33 |
| 17 | beat-Ib | 7.20 |
| 17 | beat-Ic | 7.08 |
| 17 | kek5 | 7.23 |
| 17 | CG34114 | 6.69 |
| 17 | CG17716 | 6.83 |
| 17 | CG34371 | 6.45 |
| 17 | beat-VII | 6.67 |
| 17 | Dscam3 | 6.00 |
| 17 | nolo | 5.42 |
| 17 | dpr20 | 4.58 |
| 18 | dpr13 | 16.20 |
| 18 | CG31814 | 15.48 |
| 18 | dpr9 | 12.01 |
| 18 | Dscam2 | 9.36 |
| 18 | kek1 | 9.23 |
| 18 | CG34353 | 12.07 |
| 18 | CG17716 | 12.04 |
| 18 | kek2 | 14.64 |
| 18 | beat-Vc | 8.99 |
| 18 | DIP-gamma | 8.63 |
| 18 | CG42313 | 11.55 |
| 18 | CG34114 | 8.78 |
| 18 | Ptp99A | 13.32 |
| 18 | side | 8.72 |
| 18 | dpr20 | 11.20 |
| 18 | dpr17 | 11.21 |
| 18 | fred | 11.28 |
| 18 | klg | 10.72 |
| 18 | kek3 | 8.22 |
| 18 | beat-VII | 10.28 |
| 18 | dpr6 | 7.94 |
| 18 | beat-IIIb | 8.67 |
| 18 | beat-Va | 10.43 |
| 18 | kirre | 10.13 |
| 18 | dpr8 | 11.25 |
| 18 | robo3 | 9.77 |
| 18 | beat-Ic | 8.41 |
| 18 | beat-IIa | 7.42 |
| 18 | Dscam4 | 6.23 |
| 18 | CG12484 | 6.37 |
| 18 | CG14372 | 7.26 |
| 18 | beat-IV | 7.55 |
| 19 | dpr13 | 13.45 |
| 19 | side | 15.04 |
| 19 | CG17716 | 14.89 |
| 19 | robo2 | 13.32 |
| 19 | dpr1 | 11.67 |
| 19 | CG34114 | 9.95 |
| 19 | kek1 | 12.64 |
| 19 | kek2 | 13.08 |
| 19 | Ptp99A | 12.68 |
| 19 | CG34371 | 7.70 |
| 19 | beat-IIa | 9.28 |
| 19 | dpr9 | 11.42 |
| 19 | robo3 | 8.63 |
| 19 | beat-VI | 7.92 |
| 19 | dpr10 | 7.41 |
| 19 | CG12484 | 8.05 |
| 19 | Dscam2 | 8.93 |
| 19 | beat-Ic | 6.94 |
| 19 | CG42313 | 7.38 |
| 19 | beat-IV | 6.28 |
| 20 | kek1 | 15.01 |
| 20 | Dscam2 | 10.92 |
| 20 | side-VIII | 8.79 |
| 20 | side | 12.47 |
| 20 | dpr9 | 10.70 |
| 20 | Ptp99A | 12.55 |
| 20 | tei | 9.02 |
| 20 | beat-IIa | 12.49 |
| 20 | side-II | 11.59 |
| 20 | dpr2 | 9.83 |
| 20 | kek2 | 12.22 |
| 20 | Dscam4 | 11.71 |
| 20 | beat-IIIb | 9.87 |
| 20 | kek3 | 7.97 |
| 20 | dpr10 | 7.77 |
| 20 | beat-Ic | 7.78 |
| 20 | dpr8 | 7.56 |
| 20 | beat-IIIa | 7.66 |
| 20 | kek5 | 7.25 |
| 20 | side-IV | 7.24 |
| 20 | dpr5 | 6.28 |
| 21 | robo2 | 16.42 |
| 21 | klg | 9.88 |
| 21 | CG17716 | 15.23 |
| 21 | kek1 | 15.20 |
| 21 | robo3 | 11.92 |
| 21 | kek2 | 14.58 |
| 21 | side | 14.63 |
| 21 | CG34371 | 11.49 |
| 21 | dpr9 | 14.47 |
| 21 | Ptp99A | 14.53 |
| 21 | dpr1 | 11.32 |
| 21 | dpr6 | 8.42 |
| 21 | beat-Vc | 12.99 |
| 21 | beat-Ia | 8.03 |
| 21 | beat-IIIb | 10.64 |
| 21 | dpr13 | 13.15 |
| 21 | dpr11 | 8.18 |
| 21 | dpr8 | 10.59 |
| 21 | beat-IIIa | 10.39 |
| 21 | beat-IIIc | 10.21 |
| 21 | DIP-gamma | 7.47 |
| 21 | beat-VI | 9.11 |
| 21 | dpr10 | 9.52 |
| 21 | kek3 | 9.29 |
| 21 | CG31814 | 6.44 |
| 21 | Dscam4 | 5.83 |
| 22 | Dscam4 | 11.31 |
| 22 | kek1 | 12.48 |
| 22 | dpr13 | 14.53 |
| 22 | side | 14.92 |
| 22 | CG17716 | 12.55 |
| 22 | CG34114 | 12.48 |
| 22 | dpr1 | 10.68 |
| 22 | Fas2 | 10.57 |
| 22 | dpr11 | 11.80 |
| 22 | beat-IIa | 8.20 |
| 22 | Ptp99A | 11.77 |
| 22 | Dscam2 | 11.83 |
| 22 | dpr8 | 10.10 |
| 22 | beat-IIIc | 7.98 |
| 22 | kirre | 10.88 |
| 22 | nolo | 10.93 |
| 22 | kek3 | 9.29 |
| 22 | beat-Ic | 9.23 |
| 22 | beat-Ib | 6.69 |
| 22 | dpr9 | 7.54 |
| 22 | kek2 | 5.76 |
| 22 | ed | 5.95 |
| 22 | dpr3 | 6.88 |
| 23 | side | 15.49 |
| 23 | kek1 | 11.37 |
| 23 | Ptp99A | 9.55 |
| 23 | nolo | 9.29 |
| 23 | dpr13 | 10.38 |
| 23 | dpr9 | 14.26 |
| 23 | dpr11 | 12.11 |
| 23 | kek2 | 9.02 |
| 23 | beat-IIa | 10.86 |
| 23 | beat-VI | 9.81 |
| 23 | CG17716 | 8.33 |
| 23 | Fas2 | 7.92 |
| 23 | kek3 | 7.22 |
| 23 | beat-Ia | 5.93 |
| 23 | beat-Ic | 5.89 |
| 24 | CG34353 | 11.32 |
| 24 | ed | 9.38 |
| 24 | kek1 | 12.12 |
| 24 | CG17716 | 12.61 |
| 24 | side | 9.07 |
| 24 | beat-Ic | 9.68 |
| 24 | dpr9 | 12.19 |
| 24 | Ptp99A | 11.73 |
| 24 | robo3 | 10.18 |
| 24 | CG42313 | 8.84 |
| 24 | Dscam2 | 10.62 |
| 24 | dpr8 | 9.63 |
| 24 | robo2 | 9.11 |
| 24 | kek2 | 8.14 |
| 24 | CG34114 | 9.16 |
| 24 | beat-VI | 9.52 |
| 24 | beat-Ib | 8.04 |
| 24 | kirre | 7.04 |
| 24 | beat-IIa | 7.37 |
| 24 | klg | 8.07 |
| 24 | kek3 | 6.52 |
| 24 | beat-IV | 6.50 |
| 24 | Dscam4 | 5.35 |
| 24 | dpr2 | 5.85 |
| 24 | dpr13 | 6.03 |
| 25 | CG12484 | 13.00 |
| 25 | side | 14.89 |
| 25 | kek1 | 11.71 |
| 25 | dpr9 | 9.86 |
| 25 | klg | 9.77 |
| 25 | beat-IIa | 11.06 |
| 25 | CG34353 | 13.50 |
| 25 | Dscam2 | 10.88 |
| 25 | CG17716 | 14.06 |
| 25 | CG34114 | 13.63 |
| 25 | robo2 | 9.00 |
| 25 | dpr20 | 8.88 |
| 25 | ed | 10.97 |
| 25 | Ptp99A | 10.42 |
| 25 | dpr11 | 8.84 |
| 25 | kek5 | 8.58 |
| 25 | kirre | 11.10 |
| 25 | kek2 | 9.60 |
| 25 | hbs | 8.17 |
| 25 | Dscam4 | 9.36 |
| 25 | dpr3 | 7.47 |
| 25 | dpr13 | 7.28 |
| 25 | dpr6 | 7.36 |
| 25 | CG42313 | 7.37 |
| 25 | dpr8 | 7.43 |
| 25 | beat-VI | 7.20 |
| 25 | beat-Ic | 7.09 |
| 25 | kek3 | 6.17 |
| 25 | CG14372 | 5.46 |
| 25 | dpr2 | 5.30 |
| 26 | Fas2 | 16.10 |
| 26 | Dscam2 | 12.77 |
| 26 | dpr8 | 13.79 |
| 26 | dpr6 | 10.17 |
| 26 | Ptp99A | 12.10 |
| 26 | beat-VI | 10.19 |
| 26 | beat-IIIb | 10.07 |
| 26 | CG14372 | 12.79 |
| 26 | kek2 | 11.90 |
| 26 | fred | 12.00 |
| 26 | CG34114 | 11.07 |
| 26 | robo3 | 9.19 |
| 26 | side | 9.10 |
| 26 | dpr20 | 9.00 |
| 26 | ed | 10.61 |
| 26 | CG12484 | 8.87 |
| 26 | dpr10 | 8.74 |
| 26 | CG17716 | 9.84 |
| 26 | dpr1 | 10.10 |
| 26 | CG34353 | 8.96 |
| 26 | dpr11 | 9.74 |
| 26 | hbs | 8.39 |
| 26 | kirre | 6.78 |
| 26 | otk | 7.27 |
| 26 | dpr9 | 7.33 |
| 26 | kek3 | 6.31 |
| 26 | kek1 | 6.60 |
| 26 | DIP-beta | 4.94 |
| 27 | Ptp99A | 13.36 |
| 27 | dpr1 | 10.11 |
| 27 | Fas2 | 11.79 |
| 27 | beat-VI | 11.55 |
| 27 | dpr9 | 11.75 |
| 27 | side | 10.08 |
| 27 | CG17716 | 10.57 |
| 27 | klg | 8.51 |
| 27 | kek1 | 8.92 |
| 27 | beat-VII | 10.03 |
| 27 | CG34353 | 7.61 |
| 27 | dpr6 | 8.91 |
| 27 | kek3 | 7.76 |
| 27 | Dscam2 | 7.90 |
| 27 | kek2 | 7.71 |
| 27 | ed | 9.77 |
| 27 | robo3 | 8.08 |
| 27 | otk | 7.05 |
| 27 | dpr8 | 9.28 |
| 27 | beat-Ic | 7.36 |
| 27 | CG34114 | 6.46 |
| 27 | CG14372 | 7.02 |
| 27 | kirre | 6.16 |
| 27 | Dscam4 | 6.56 |
| 28 | klg | 13.04 |
| 28 | Dscam2 | 13.16 |
| 28 | beat-IIa | 13.33 |
| 28 | CG42313 | 10.34 |
| 28 | CG12484 | 9.70 |
| 28 | Ptp99A | 12.60 |
| 28 | CG17716 | 9.69 |
| 28 | kek5 | 8.48 |
| 28 | Dscam4 | 8.29 |
| 28 | kirre | 8.43 |
| 28 | fred | 8.56 |
| 28 | side | 8.31 |
| 28 | ed | 7.49 |
| 28 | DIP-iota | 8.04 |
| 28 | Fas2 | 7.21 |
| 28 | dpr13 | 7.91 |
| 28 | dpr9 | 7.28 |
| 28 | beat-VI | 7.19 |
| 28 | dpr1 | 7.19 |
| 28 | dpr18 | 6.38 |
| 28 | kek1 | 7.09 |
| 28 | dpr11 | 6.00 |
| 28 | hbs | 6.01 |
| 28 | DIP-gamma | 6.65 |
| 28 | nolo | 5.87 |
| 28 | dpr20 | 6.13 |
| 28 | hig | 5.83 |
| 28 | CG14372 | 4.67 |
| 28 | beat-Vc | 6.58 |
| 29 | CG17716 | 11.31 |
| 29 | CG42313 | 10.32 |
| 29 | dpr1 | 10.24 |
| 29 | CG34114 | 10.17 |
| 29 | Dscam2 | 10.00 |
| 29 | kek1 | 12.50 |
| 29 | Ptp99A | 11.10 |
| 29 | dpr9 | 10.98 |
| 29 | kek2 | 12.18 |
| 29 | klg | 9.99 |
| 29 | dpr13 | 9.76 |
| 29 | Dscam4 | 12.17 |
| 29 | kirre | 9.40 |
| 29 | dpr6 | 9.35 |
| 29 | CG34353 | 9.69 |
| 29 | hig | 8.23 |
| 29 | hbs | 8.30 |
| 29 | beat-Ia | 7.62 |
| 29 | dpr2 | 7.01 |
| 29 | dpr10 | 6.75 |
| 29 | beat-Va | 6.75 |
| 29 | kek5 | 6.75 |
| 29 | CG14372 | 6.46 |
| 29 | robo3 | 6.58 |
| 29 | dpr3 | 6.21 |
| 29 | beat-IIIb | 6.56 |
| 29 | beat-IIIc | 6.63 |
| 29 | Fas2 | 6.08 |
| 29 | ed | 5.14 |
| 30 | CG17716 | 12.73 |
| 30 | dpr8 | 11.78 |
| 30 | klg | 11.37 |
| 30 | beat-VI | 10.74 |
| 30 | kek1 | 13.37 |
| 30 | beat-IIa | 11.55 |
| 30 | dpr9 | 12.56 |
| 30 | dpr6 | 9.27 |
| 30 | Ptp99A | 10.68 |
| 30 | kek2 | 9.74 |
| 30 | dpr1 | 9.84 |
| 30 | Dscam2 | 11.30 |
| 30 | side | 9.48 |
| 30 | dpr11 | 8.26 |
| 30 | dpr13 | 9.07 |
| 30 | dpr2 | 7.86 |
| 30 | dpr10 | 9.25 |
| 30 | beat-IIIb | 8.24 |
| 30 | kek3 | 8.11 |
| 30 | CG34114 | 6.97 |
| 30 | kirre | 7.04 |
| 30 | dpr3 | 6.98 |
| 30 | Fas2 | 8.30 |
| 30 | beat-Ic | 7.03 |
| 30 | Dscam4 | 7.16 |
| 30 | CG34371 | 6.36 |
| 31 | kek1 | 10.83 |
| 31 | Ptp99A | 12.69 |
| 31 | side | 10.84 |
| 31 | beat-VI | 10.69 |
| 31 | dpr13 | 10.49 |
| 31 | klg | 10.31 |
| 31 | CG12484 | 10.18 |
| 31 | CG17716 | 9.96 |
| 31 | Dscam4 | 10.71 |
| 31 | dpr9 | 9.74 |
| 31 | dpr10 | 7.45 |
| 31 | kek2 | 8.07 |
| 31 | kirre | 8.86 |
| 31 | beat-IIIa | 6.93 |
| 31 | dpr1 | 7.72 |
| 31 | dpr11 | 7.05 |
| 31 | beat-IIa | 6.49 |
| 31 | Dscam2 | 8.15 |
| 31 | CG14372 | 7.65 |
| 31 | CG34114 | 6.83 |
| 31 | CG31814 | 6.83 |
| 31 | ed | 6.97 |
| 32 | kek2 | 15.47 |
| 32 | klg | 12.74 |
| 32 | beat-VI | 9.75 |
| 32 | CG34114 | 11.03 |
| 32 | Fas2 | 11.96 |
| 32 | CG34353 | 14.44 |
| 32 | beat-VII | 11.57 |
| 32 | dpr9 | 11.73 |
| 32 | beat-Ia | 10.83 |
| 32 | dpr13 | 11.51 |
| 32 | dpr1 | 8.72 |
| 32 | dpr8 | 8.95 |
| 32 | dpr17 | 8.26 |
| 32 | beat-IIa | 11.15 |
| 32 | CG17716 | 8.19 |
| 32 | DIP-gamma | 7.91 |
| 32 | Ptp99A | 9.74 |
| 32 | Dscam4 | 9.22 |
| 32 | kek5 | 7.26 |
| 32 | dpr6 | 6.99 |
| 32 | dpr18 | 4.90 |
| 32 | dpr11 | 5.65 |
| 32 | CG12484 | 7.13 |
| 33 | CG17716 | 14.29 |
| 33 | kek1 | 15.58 |
| 33 | dpr13 | 13.05 |
| 33 | dpr5 | 9.75 |
| 33 | CG12484 | 9.04 |
| 33 | otk | 11.78 |
| 33 | dpr9 | 11.20 |
| 33 | beat-IIIb | 10.67 |
| 33 | otk2 | 7.73 |
| 33 | side | 8.13 |
| 33 | klg | 10.11 |
| 33 | beat-Vc | 9.10 |
| 33 | CG42313 | 8.02 |
| 33 | beat-IIIa | 10.85 |
| 33 | dpr8 | 9.07 |
| 33 | Dscam2 | 7.63 |
| 33 | Ptp99A | 8.54 |
| 33 | CG34114 | 9.98 |
| 33 | kek2 | 8.96 |
| 33 | beat-Va | 7.33 |
| 33 | CG34353 | 7.07 |
| 33 | dpr11 | 7.02 |
| 33 | Fas2 | 6.45 |
| 34 | CG17716 | 16.01 |
| 34 | dpr13 | 11.60 |
| 34 | dpr8 | 9.34 |
| 34 | kek1 | 12.15 |
| 34 | kek2 | 10.80 |
| 34 | kek5 | 11.08 |
| 34 | dpr11 | 8.85 |
| 34 | kek3 | 8.60 |
| 34 | otk | 9.34 |
| 34 | Dscam2 | 8.47 |
| 34 | Dscam4 | 8.59 |
| 34 | Ptp99A | 9.14 |
| 34 | dpr9 | 7.78 |
| 34 | beat-VI | 8.54 |
| 34 | beat-IIa | 8.84 |
| 34 | CG42313 | 8.43 |
| 34 | robo3 | 8.41 |
| 34 | beat-Vc | 7.06 |
| 34 | klg | 7.10 |
| 34 | DIP-theta | 7.36 |
| 34 | Fas2 | 6.29 |
| 35 | kek1 | 15.88 |
| 35 | CG12484 | 12.10 |
| 35 | robo2 | 10.55 |
| 35 | klg | 12.28 |
| 35 | robo3 | 14.69 |
| 35 | CG17716 | 11.24 |
| 35 | kek3 | 10.81 |
| 35 | Ptp99A | 14.37 |
| 35 | beat-IIIb | 13.95 |
| 35 | kek2 | 14.13 |
| 35 | dpr13 | 11.04 |
| 35 | kek5 | 11.51 |
| 35 | beat-IIIc | 11.13 |
| 35 | otk | 7.83 |
| 35 | Dscam4 | 10.13 |
| 35 | dpr9 | 7.42 |
| 35 | side | 9.38 |
| 35 | beat-IIIa | 8.76 |
| 35 | kirre | 9.25 |
| 35 | CG14372 | 8.52 |
| 35 | DIP-beta | 7.66 |
| 35 | beat-IIa | 6.49 |
| 35 | beat-IV | 6.86 |
| 35 | dpr10 | 7.23 |
| 35 | dpr18 | 6.78 |
| 36 | dpr13 | 13.35 |
| 36 | Fas2 | 11.08 |
| 36 | CG17716 | 14.52 |
| 36 | side | 13.57 |
| 36 | Ptp99A | 12.13 |
| 36 | beat-VI | 10.53 |
| 36 | CG34353 | 9.23 |
| 36 | Dscam2 | 9.62 |
| 36 | robo2 | 10.76 |
| 36 | klg | 10.09 |
| 36 | beat-IIa | 9.21 |
| 36 | robo3 | 9.78 |
| 36 | Dscam3 | 8.39 |
| 36 | otk2 | 9.86 |
| 36 | dpr1 | 8.41 |
| 36 | dpr9 | 7.64 |
| 36 | dpr10 | 8.34 |
| 36 | CG34114 | 7.15 |
| 36 | kek2 | 9.86 |
| 36 | hig | 7.62 |
| 36 | Dscam4 | 12.21 |
| 36 | kirre | 7.64 |
| 36 | CG12484 | 7.61 |
| 36 | ed | 7.95 |
| 36 | fred | 7.79 |
| 36 | dpr8 | 6.82 |
| 36 | kek1 | 6.71 |
| 37 | Ptp99A | 10.33 |
| 37 | Dscam2 | 12.68 |
| 37 | dpr1 | 10.98 |
| 37 | CG42313 | 12.79 |
| 37 | side | 8.68 |
| 37 | beat-IIa | 12.41 |
| 37 | dpr9 | 14.62 |
| 37 | CG17716 | 11.46 |
| 37 | klg | 13.86 |
| 37 | beat-VII | 7.88 |
| 37 | robo3 | 12.18 |
| 37 | CG14372 | 7.25 |
| 37 | fred | 7.40 |
| 37 | kirre | 8.31 |
| 37 | kek1 | 7.09 |
| 37 | beat-VI | 6.49 |
| 37 | dpr6 | 7.62 |
| 37 | dpr11 | 5.81 |
| 37 | CG12484 | 7.26 |
| 37 | beat-IV | 7.31 |
| 38 | Dscam2 | 15.46 |
| 38 | CG42313 | 14.73 |
| 38 | kek1 | 14.39 |
| 38 | CG12484 | 14.69 |
| 38 | CG17716 | 14.97 |
| 38 | side | 14.07 |
| 38 | beat-Ic | 13.75 |
| 38 | kek2 | 13.45 |
| 38 | kek3 | 13.42 |
| 38 | Ptp99A | 13.57 |
| 38 | dpr6 | 13.45 |
| 38 | beat-Ia | 12.09 |
| 38 | kirre | 12.31 |
| 38 | beat-Ib | 9.17 |
| 38 | dpr9 | 12.32 |
| 38 | beat-IIa | 12.81 |
| 38 | Fas2 | 13.01 |
| 38 | dpr1 | 8.19 |
| 38 | dpr2 | 8.95 |
| 38 | beat-VI | 10.30 |
| 38 | dpr13 | 9.94 |
| 38 | dpr10 | 9.38 |
| 38 | dpr8 | 11.05 |
| 38 | DIP-beta | 8.06 |
| 38 | otk2 | 7.32 |
| 38 | beat-Va | 7.87 |
| 38 | beat-Vc | 8.17 |
| 38 | CG14372 | 5.87 |
| 38 | hbs | 8.13 |
| 38 | otk | 7.01 |
| 39 | Dscam2 | 17.24 |
| 39 | dpr8 | 14.46 |
| 39 | Ptp99A | 14.97 |
| 39 | CG17716 | 12.36 |
| 39 | dpr9 | 14.71 |
| 39 | dpr1 | 14.87 |
| 39 | klg | 11.62 |
| 39 | side | 12.64 |
| 39 | kek2 | 10.02 |
| 39 | dpr13 | 11.91 |
| 39 | kek1 | 11.15 |
| 39 | dpr11 | 12.42 |
| 39 | dpr5 | 12.99 |
| 39 | CG12484 | 10.43 |
| 39 | dpr3 | 9.78 |
| 39 | beat-VI | 9.66 |
| 39 | dpr20 | 9.89 |
| 39 | DIP-delta | 10.51 |
| 39 | beat-IIa | 10.40 |
| 39 | dpr18 | 8.67 |
| 39 | dpr2 | 9.34 |
| 39 | kirre | 7.65 |
| 39 | CG34114 | 7.12 |
| 39 | Dscam4 | 8.08 |
| 39 | beat-Va | 6.45 |
| 40 | Dscam2 | 12.10 |
| 40 | dpr9 | 11.14 |
| 40 | side | 9.62 |
| 40 | CG17716 | 13.56 |
| 40 | dpr13 | 9.37 |
| 40 | dpr1 | 10.71 |
| 40 | beat-IIa | 12.45 |
| 40 | Ptp99A | 10.30 |
| 40 | kek1 | 8.04 |
| 40 | dpr17 | 7.44 |
| 40 | Dscam4 | 8.21 |
| 40 | beat-VII | 7.72 |
| 40 | dpr6 | 9.45 |
| 40 | beat-VI | 8.59 |
| 40 | kirre | 8.92 |
| 40 | Fas2 | 8.90 |
| 40 | dpr8 | 7.79 |
| 40 | kek3 | 7.03 |
| 40 | CG34114 | 6.36 |
| 40 | klg | 7.89 |
| 40 | kek2 | 6.74 |
| 41 | Ptp99A | 16.16 |
| 41 | dpr11 | 13.90 |
| 41 | dpr13 | 15.70 |
| 41 | CG42313 | 13.72 |
| 41 | CG17716 | 13.37 |
| 41 | Dscam3 | 12.37 |
| 41 | robo3 | 14.67 |
| 41 | Dscam2 | 12.03 |
| 41 | kek5 | 12.04 |
| 41 | dpr1 | 9.39 |
| 41 | dpr6 | 10.57 |
| 41 | kek2 | 7.67 |
| 41 | beat-IIa | 8.85 |
| 41 | dpr8 | 8.42 |
| 41 | dpr10 | 9.04 |
| 41 | kek1 | 8.73 |
| 41 | beat-Va | 6.16 |
| 42 | CG17716 | 16.32 |
| 42 | Dscam2 | 15.57 |
| 42 | CG12484 | 15.51 |
| 42 | dpr20 | 14.89 |
| 42 | dpr2 | 14.64 |
| 42 | kek1 | 14.12 |
| 42 | side | 14.10 |
| 42 | dpr13 | 13.93 |
| 42 | otk2 | 13.82 |
| 42 | otk | 13.73 |
| 42 | robo3 | 13.52 |
| 42 | dpr9 | 13.38 |
| 42 | Fas2 | 12.68 |
| 42 | kirre | 12.57 |
| 42 | kek2 | 12.14 |
| 42 | CG34353 | 11.39 |
| 42 | klg | 10.93 |
| 42 | Dscam4 | 10.49 |
| 42 | dpr11 | 8.46 |
| 42 | dpr1 | 8.10 |
| 42 | beat-Vc | 7.55 |
| 42 | beat-Ia | 7.32 |
| 42 | beat-VII | 7.19 |
| 42 | beat-Ib | 7.14 |
| 42 | CG14372 | 7.13 |
| 42 | dpr17 | 7.07 |
| 42 | Ptp99A | 7.02 |
| 42 | kek3 | 6.97 |
| 42 | robo2 | 6.81 |
| 42 | CG42313 | 6.63 |
| 42 | dpr6 | 6.18 |
| 42 | CG34114 | 5.92 |
| 42 | CG34371 | 5.29 |
| 42 | dpr3 | 5.06 |
| 42 | hig | 4.63 |
| 42 | beat-Va | 4.10 |
| 42 | hbs | 3.02 |
| 42 | DIP-gamma | 2.53 |
| 43 | side | 10.41 |
| 43 | dpr8 | 13.44 |
| 43 | klg | 13.37 |
| 43 | Dscam2 | 12.26 |
| 43 | CG42313 | 12.33 |
| 43 | CG17716 | 9.31 |
| 43 | beat-IIa | 12.68 |
| 43 | CG34353 | 10.31 |
| 43 | beat-VII | 10.28 |
| 43 | dpr13 | 10.05 |
| 43 | DIP-delta | 10.38 |
| 43 | kek5 | 9.91 |
| 43 | dpr9 | 9.38 |
| 43 | hbs | 8.50 |
| 43 | Ptp99A | 9.74 |
| 43 | Dscam4 | 8.45 |
| 43 | nolo | 6.03 |
| 43 | DIP-theta | 7.73 |
| 43 | beat-IIIa | 6.81 |
| 44 | dpr13 | 12.94 |
| 44 | CG42313 | 11.53 |
| 44 | CG17716 | 12.62 |
| 44 | Dscam2 | 9.20 |
| 44 | side | 8.82 |
| 44 | kek1 | 10.54 |
| 44 | Ptp99A | 10.33 |
| 44 | klg | 9.13 |
| 44 | nolo | 8.74 |
| 44 | dpr9 | 7.99 |
| 44 | beat-IIa | 9.15 |
| 44 | beat-VI | 7.98 |
| 44 | kirre | 6.37 |
| 44 | kek3 | 6.81 |
| 44 | Dscam4 | 6.52 |
| 45 | Dscam2 | 13.90 |
| 45 | beat-Ic | 14.50 |
| 45 | kek1 | 14.22 |
| 45 | CG17716 | 12.24 |
| 45 | dpr13 | 12.02 |
| 45 | CG34114 | 10.00 |
| 45 | DIP-gamma | 8.68 |
| 45 | beat-VI | 10.01 |
| 45 | kek3 | 8.15 |
| 45 | dpr10 | 11.84 |
| 45 | DIP-eta | 9.32 |
| 45 | beat-Ib | 9.04 |
| 45 | klg | 8.49 |
| 45 | otk | 8.23 |
| 45 | dpr9 | 8.16 |
| 45 | Ptp99A | 7.68 |
| 45 | Dscam4 | 7.64 |
| 45 | kek2 | 7.09 |
| 45 | DIP-beta | 7.25 |
| 45 | dpr8 | 7.36 |
| 45 | dpr6 | 7.39 |
| 45 | hbs | 6.02 |
| 45 | CG42313 | 6.23 |
| 46 | Dscam2 | 15.59 |
| 46 | klg | 14.72 |
| 46 | beat-VI | 13.72 |
| 46 | side | 13.07 |
| 46 | dpr13 | 12.17 |
| 46 | kek1 | 14.96 |
| 46 | dpr1 | 11.37 |
| 46 | Ptp99A | 13.54 |
| 46 | kek2 | 11.84 |
| 46 | beat-IV | 9.86 |
| 46 | kirre | 11.48 |
| 46 | CG17716 | 9.49 |
| 46 | DIP-delta | 11.80 |
| 46 | dpr8 | 9.27 |
| 46 | CG12484 | 9.56 |
| 46 | dpr9 | 11.91 |
| 46 | CG34353 | 8.84 |
| 46 | Dscam4 | 9.89 |
| 46 | beat-VII | 7.69 |
| 46 | ed | 8.55 |
| 46 | beat-Ic | 7.28 |
| 46 | beat-IIa | 6.39 |
| 46 | kek5 | 6.55 |
| 46 | CG42313 | 6.34 |
| 47 | klg | 13.02 |
| 47 | beat-VI | 12.95 |
| 47 | Dscam2 | 12.05 |
| 47 | beat-IIa | 11.64 |
| 47 | CG17716 | 8.81 |
| 47 | dpr8 | 11.43 |
| 47 | kirre | 12.24 |
| 47 | Ptp99A | 9.77 |
| 47 | beat-Ic | 8.62 |
| 47 | Dscam4 | 10.60 |
| 47 | robo3 | 8.42 |
| 47 | dpr6 | 8.41 |
| 47 | ed | 10.60 |
| 47 | dpr18 | 7.30 |
| 47 | CG14372 | 7.32 |
| 47 | beat-VII | 8.19 |
| 47 | dpr9 | 7.22 |
| 47 | dpr1 | 8.18 |
| 47 | side | 8.01 |
| 47 | otk | 6.91 |
| 47 | dpr10 | 6.77 |
| 47 | Fas2 | 6.85 |
| 47 | beat-IIIc | 6.71 |
| 47 | kek5 | 5.89 |
| 48 | dpr1 | 12.34 |
| 48 | CG17716 | 14.98 |
| 48 | Ptp99A | 15.28 |
| 48 | beat-VI | 11.63 |
| 48 | CG34114 | 9.65 |
| 48 | beat-Ic | 12.85 |
| 48 | kek1 | 13.23 |
| 48 | dpr9 | 14.15 |
| 48 | dpr8 | 14.00 |
| 48 | hbs | 10.66 |
| 48 | side | 9.72 |
| 48 | Dscam2 | 8.94 |
| 48 | beat-Ib | 11.87 |
| 48 | otk | 10.45 |
| 48 | Dscam4 | 8.99 |
| 48 | beat-IIa | 12.00 |
| 48 | dpr10 | 9.87 |
| 48 | Fas2 | 9.70 |
| 48 | DIP-beta | 11.25 |
| 48 | dpr13 | 8.74 |
| 48 | beat-VII | 9.62 |
| 48 | DIP-eta | 7.44 |
| 48 | ed | 7.79 |
| 48 | nolo | 7.82 |
| 48 | hig | 7.78 |
| 48 | DIP-delta | 10.15 |
| 48 | beat-IV | 7.16 |
| 48 | beat-Ia | 7.43 |
| 48 | Dscam3 | 6.62 |
| 48 | dpr6 | 7.44 |
| 48 | dpr20 | 6.41 |
| 48 | CG34353 | 6.23 |
| 48 | CG31814 | 5.21 |
| 48 | kek2 | 5.10 |
| 49 | klg | 15.68 |
| 49 | beat-VI | 10.65 |
| 49 | dpr13 | 14.44 |
| 49 | Dscam2 | 14.95 |
| 49 | side | 14.99 |
| 49 | Fas2 | 13.99 |
| 49 | DIP-gamma | 9.59 |
| 49 | beat-Ic | 9.79 |
| 49 | CG34353 | 14.21 |
| 49 | kek3 | 9.27 |
| 49 | beat-VII | 9.51 |
| 49 | dpr8 | 9.46 |
| 49 | dpr9 | 8.56 |
| 49 | dpr10 | 8.40 |
| 49 | kek2 | 8.68 |
| 49 | kek1 | 9.24 |
| 49 | CG17716 | 13.06 |
| 49 | Dscam4 | 11.77 |
| 49 | hig | 12.88 |
| 49 | dpr1 | 9.29 |
| 49 | CG14372 | 8.17 |
| 49 | robo3 | 8.10 |
| 49 | kirre | 10.75 |
| 49 | beat-IIIb | 7.70 |
| 49 | Ptp99A | 11.55 |
| 49 | dpr18 | 7.84 |
| 49 | beat-Va | 7.51 |
| 49 | dpr3 | 7.95 |
| 49 | CG12484 | 8.41 |
| 49 | beat-Vc | 7.29 |
| 49 | kek5 | 7.23 |
| 50 | CG17716 | 15.45 |
| 50 | dpr13 | 12.27 |
| 50 | kek2 | 11.46 |
| 50 | dpr11 | 9.22 |
| 50 | beat-IIIb | 10.82 |
| 50 | kek3 | 10.75 |
| 50 | kek1 | 10.58 |
| 50 | Dscam4 | 10.42 |
| 50 | otk2 | 7.99 |
| 50 | Dscam2 | 9.30 |
| 50 | beat-IIa | 10.79 |
| 50 | dpr10 | 8.35 |
| 50 | dpr8 | 9.29 |
| 50 | Ptp99A | 9.28 |
| 50 | kek5 | 7.03 |
| 50 | Dscam3 | 7.34 |
| 50 | beat-VI | 8.00 |
| 50 | otk | 6.52 |
| 51 | kek1 | 14.30 |
| 51 | robo2 | 8.74 |
| 51 | dpr1 | 11.75 |
| 51 | dpr13 | 10.91 |
| 51 | CG17716 | 10.89 |
| 51 | side | 10.51 |
| 51 | Dscam2 | 12.44 |
| 51 | DIP-beta | 11.55 |
| 51 | Ptp99A | 13.57 |
| 51 | kek3 | 11.12 |
| 51 | CG42313 | 11.23 |
| 51 | kek2 | 9.01 |
| 51 | beat-IV | 9.19 |
| 51 | robo3 | 13.10 |
| 51 | Dscam4 | 8.01 |
| 51 | dpr8 | 8.49 |
| 51 | CG14372 | 7.36 |
| 51 | dpr10 | 6.82 |
| 51 | dpr9 | 7.55 |
| 51 | ed | 6.29 |
| 51 | kek5 | 6.52 |
| 51 | fred | 7.16 |
| 51 | nolo | 8.15 |
| 51 | hbs | 7.75 |
| 51 | CG12484 | 7.71 |
| 52 | dpr9 | 16.19 |
| 52 | Dscam2 | 15.40 |
| 52 | side | 16.06 |
| 52 | beat-IIa | 13.61 |
| 52 | CG17716 | 14.40 |
| 52 | CG34114 | 12.14 |
| 52 | CG42313 | 10.07 |
| 52 | CG12484 | 11.29 |
| 52 | dpr2 | 13.82 |
| 52 | beat-Ic | 9.04 |
| 52 | dpr18 | 8.89 |
| 52 | dpr6 | 8.88 |
| 52 | Dscam4 | 10.38 |
| 52 | fred | 12.47 |
| 52 | dpr10 | 9.01 |
| 52 | beat-VII | 7.98 |
| 52 | kek2 | 8.28 |
| 52 | ed | 7.45 |
| 52 | Ptp99A | 7.36 |
| 52 | kek1 | 7.13 |
| 52 | robo3 | 6.91 |
| 52 | CG31814 | 6.22 |
| 52 | beat-Ia | 5.53 |
| 52 | otk | 6.00 |
| 52 | dpr8 | 5.40 |
| 52 | kek5 | 4.29 |
| 53 | klg | 16.42 |
| 53 | side | 15.28 |
| 53 | kek1 | 10.54 |
| 53 | kirre | 11.71 |
| 53 | beat-IIIb | 9.55 |
| 53 | dpr10 | 13.55 |
| 53 | CG34114 | 9.44 |
| 53 | dpr8 | 11.60 |
| 53 | dpr9 | 13.68 |
| 53 | hbs | 10.76 |
| 53 | otk | 9.07 |
| 53 | Dscam2 | 9.30 |
| 53 | beat-IIa | 13.17 |
| 53 | CG31814 | 9.32 |
| 53 | dpr17 | 9.10 |
| 53 | Ptp99A | 8.48 |
| 53 | CG17716 | 8.74 |
| 53 | fred | 7.97 |
| 53 | Dscam4 | 10.79 |
| 53 | beat-VI | 8.09 |
| 53 | Dscam3 | 7.57 |
| 53 | CG34353 | 7.58 |
| 53 | Fas2 | 7.28 |
| 53 | kek2 | 7.24 |
| 53 | dpr6 | 7.41 |
| 53 | dpr1 | 6.93 |
| 53 | beat-Ic | 6.73 |
| 53 | ed | 6.37 |
| 53 | robo3 | 5.69 |
| 53 | CG34371 | 5.30 |
| 54 | Dscam2 | 13.09 |
| 54 | CG17716 | 9.01 |
| 54 | CG42313 | 10.78 |
| 54 | side | 10.54 |
| 54 | klg | 12.65 |
| 54 | beat-IIa | 12.47 |
| 54 | dpr8 | 8.93 |
| 54 | CG34353 | 9.28 |
| 54 | kek2 | 8.56 |
| 54 | kek1 | 7.32 |
| 54 | Ptp99A | 8.09 |
| 54 | beat-VII | 8.80 |
| 54 | kirre | 8.29 |
| 54 | DIP-delta | 7.90 |
| 54 | Dscam4 | 7.73 |
| 54 | dpr9 | 6.68 |
| 54 | dpr6 | 6.45 |
| 54 | beat-VI | 6.19 |
| 54 | otk | 6.10 |
| 55 | dpr1 | 14.19 |
| 55 | kek1 | 15.29 |
| 55 | CG17716 | 13.46 |
| 55 | otk | 10.49 |
| 55 | beat-Ic | 8.95 |
| 55 | dpr8 | 9.07 |
| 55 | beat-VI | 10.56 |
| 55 | kek5 | 9.89 |
| 55 | otk2 | 11.54 |
| 55 | Ptp99A | 10.20 |
| 55 | hig | 9.87 |
| 55 | ed | 10.29 |
| 55 | Dscam4 | 9.24 |
| 55 | beat-IIa | 8.95 |
| 55 | hbs | 8.51 |
| 55 | beat-VII | 7.94 |
| 55 | CG34114 | 7.94 |
| 55 | DIP-gamma | 7.47 |
| 55 | CG34353 | 7.06 |
| 55 | side | 7.10 |
| 55 | dpr13 | 7.23 |
| 55 | Dscam2 | 6.71 |
| 55 | CG42313 | 7.12 |
| 55 | dpr20 | 6.54 |
| 55 | fred | 6.41 |
| 55 | kek2 | 6.26 |
| 56 | Ptp99A | 14.90 |
| 56 | CG42313 | 12.64 |
| 56 | dpr13 | 12.59 |
| 56 | CG34353 | 10.15 |
| 56 | CG17716 | 14.84 |
| 56 | dpr11 | 12.22 |
| 56 | kek1 | 9.62 |
| 56 | Fas2 | 9.98 |
| 56 | Dscam2 | 11.22 |
| 56 | dpr8 | 9.35 |
| 56 | robo3 | 9.12 |
| 56 | kek2 | 10.50 |
| 56 | dpr9 | 10.72 |
| 56 | kirre | 12.14 |
| 56 | dpr6 | 10.75 |
| 56 | beat-Vc | 10.52 |
| 56 | kek5 | 8.26 |
| 56 | Dscam4 | 7.88 |
| 56 | beat-IIa | 8.31 |
| 56 | robo2 | 7.90 |
| 56 | dpr1 | 8.13 |
| 56 | side | 7.63 |
| 56 | CG12484 | 7.48 |
| 56 | beat-IIIb | 6.88 |
| 56 | otk2 | 6.52 |
| 56 | fred | 6.56 |
| 56 | CG34114 | 6.16 |
| 56 | ed | 6.20 |
| 56 | hbs | 6.26 |
| 56 | Dscam3 | 6.03 |
| 56 | CG34371 | 5.69 |
| 57 | side | 15.60 |
| 57 | beat-VI | 13.66 |
| 57 | dpr13 | 13.95 |
| 57 | Dscam2 | 13.29 |
| 57 | Fas2 | 13.30 |
| 57 | CG17716 | 12.46 |
| 57 | CG34353 | 11.16 |
| 57 | kek1 | 10.13 |
| 57 | dpr6 | 11.99 |
| 57 | kek2 | 13.81 |
| 57 | otk2 | 8.00 |
| 57 | robo3 | 11.48 |
| 57 | robo2 | 8.29 |
| 57 | kirre | 12.93 |
| 57 | otk | 9.18 |
| 57 | dpr20 | 8.23 |
| 57 | DIP-gamma | 7.95 |
| 57 | CG34371 | 9.09 |
| 57 | Dscam3 | 7.48 |
| 57 | dpr9 | 7.82 |
| 57 | dpr10 | 8.35 |
| 57 | dpr18 | 7.71 |
| 57 | beat-Vc | 7.36 |
| 57 | Ptp99A | 8.29 |
| 57 | beat-Va | 5.89 |
| 57 | beat-IIa | 6.58 |
| 57 | beat-VII | 6.79 |
| 57 | dpr17 | 5.40 |
| 58 | CG17716 | 16.43 |
| 58 | Ptp99A | 15.09 |
| 58 | dpr13 | 15.89 |
| 58 | Fas2 | 14.92 |
| 58 | robo2 | 12.92 |
| 58 | dpr6 | 12.78 |
| 58 | otk | 9.99 |
| 58 | DIP-delta | 9.73 |
| 58 | hig | 11.54 |
| 58 | side | 13.88 |
| 58 | kek2 | 13.62 |
| 58 | dpr1 | 8.05 |
| 58 | CG34371 | 13.51 |
| 58 | CG34114 | 7.67 |
| 58 | Dscam3 | 9.44 |
| 58 | dpr10 | 11.17 |
| 58 | beat-VI | 7.69 |
| 58 | otk2 | 7.49 |
| 58 | DIP-theta | 7.31 |
| 58 | Dscam4 | 11.84 |
| 58 | robo3 | 12.11 |
| 58 | beat-IV | 8.73 |
| 58 | CG34353 | 8.41 |
| 58 | dpr8 | 6.54 |
| 58 | dpr18 | 6.18 |
| 59 | CG17716 | 13.95 |
| 59 | beat-IIIc | 10.57 |
| 59 | Ptp99A | 14.33 |
| 59 | kek3 | 12.95 |
| 59 | kek2 | 13.62 |
| 59 | kek1 | 12.54 |
| 59 | beat-IIIb | 11.22 |
| 59 | otk2 | 11.38 |
| 59 | klg | 10.05 |
| 59 | CG12484 | 9.56 |
| 59 | otk | 8.17 |
| 59 | dpr9 | 10.78 |
| 59 | CG42313 | 10.49 |
| 59 | beat-Ic | 8.91 |
| 59 | Dscam2 | 10.12 |
| 59 | robo3 | 9.54 |
| 59 | Dscam4 | 9.22 |
| 59 | CG34114 | 7.63 |
| 59 | kirre | 7.26 |
| 59 | dpr10 | 7.23 |
| 59 | beat-VI | 8.11 |
| 59 | beat-IIa | 8.32 |
| 59 | side | 7.03 |
| 59 | dpr8 | 7.21 |
| 59 | ed | 7.26 |
| 59 | dpr6 | 6.94 |
| 59 | CG34371 | 5.47 |
| 60 | dpr13 | 14.00 |
| 60 | Dscam2 | 14.82 |
| 60 | CG42313 | 10.79 |
| 60 | beat-Ib | 12.23 |
| 60 | DIP-eta | 10.43 |
| 60 | ed | 12.02 |
| 60 | CG34114 | 10.18 |
| 60 | kek3 | 13.90 |
| 60 | beat-Ic | 10.69 |
| 60 | Ptp99A | 13.56 |
| 60 | DIP-beta | 8.20 |
| 60 | dpr1 | 8.52 |
| 60 | kek2 | 9.24 |
| 60 | kek1 | 9.37 |
| 60 | CG34371 | 11.92 |
| 60 | kirre | 10.48 |
| 60 | otk2 | 8.69 |
| 60 | beat-VI | 8.40 |
| 60 | dpr8 | 6.88 |
| 60 | beat-IIa | 5.97 |

**Dataset EV1: Homeodomain and Ig domain expression in scRNA- Seq identified clusters.**

Single cells were grouped into 60 clusters according to differential homeodomain TF expression (see Figure 2A) and then arranged along the AP position (from anterior to posterior: Cluster 1-60) (see Figure 1D). For each cluster the mean gene expression was computed with a threshold of 4. The table depicts homeo- and Ig- encoding genes in columns and clusters in rows.
