# Supplementary material for: Single‐cell RNA sequencing of motoneurons identifies regulators of synaptic wiring in Drosophila embryos
Source: Mol Syst Biol. 2022 Feb 28;18(3):e10255. doi: 10.15252/msb.202110255 (PMC8883443; doi:10.15252/msb.202110255)
Supplement: Supplementary file 3 [file MSB-18-e10255-s002.pdf]

molecular systems biology

Single-cell RNA sequencing of motoneurons identifies regulators of synaptic wiring in *Drosophila* embryos

Jessica Velten, Xuefan Gao, Rashi Agarwal, Patrick Van Nierop y Sanchez, Katrin Domsch, Lena Bognar, Malte Paulsen, Lars Velten, Ingrid Lohmann,
DOI: 10.15252/msb.202110255

Corresponding author(s): Ingrid Lohmann (ingrid.lohmann@cos.uni-heidelberg.de) , Lars Velten (lars.velten@crg.eu)

Review Timeline:

Submission Date:	28th Jan 21
Editorial Decision:	25th Feb 21
Revision Received:	30th Sep 21
Editorial Decision:	10th Dec 21
Revision Received:	28th Jan 22
Accepted:	7th Feb 22

Editor: Jingyi Hou

Transaction Report:

(Note: With the exception of the correction of typographical or spelling errors that could be a source of ambiguity, letters and reports are not edited. Depending on transfer agreements, referee reports obtained elsewhere may or may not be included in this compilation. Referee reports are anonymous unless the Referee chooses to sign their reports.)

Thank you for submitting your work to Molecular Systems Biology. We have now heard back from the three reviewers who agreed to evaluate your study. As you will see below, the reviewers find the topic of your study of interest. However, they raise substantial concerns about your work, which should be convincingly addressed in a major revision of the present manuscript.

Without reiterating all the points listed below, the most fundamental issues that need to be convincingly addressed are the following:

-Both Reviewers #1 and #2 pointed out that the study remains somewhat preliminary, and several main conclusions are not fully supported by the data. Therefore, some follow-up experiments are required to support the claims that 1) a 'homeodomain TF code' is sufficient to determine the identity of motoneurons and 2) Ig-domain proteins mediate the effects of homeodomain TFs, as recommended by reviewers.
-Further investigations and support for the presented phenotypes need to be provided, as commented by Reviewer #3.
-Overinterpretation should be avoided.

All other issues raised by the reviewers need to be satisfactorily addressed as well.

Overall it is clear that publication of the manuscript cannot be considered at this stage. Addressing the reviewers' concerns in full will be necessary for further considering the manuscript in our journal and this appears to require a lot of additional work and experimentation. I am unsure whether you will be able or willing to address those and return a revised manuscript. On the other hand, given the potential interest of the findings, I would be willing to consider a revised manuscript with the understanding that the reviewers' concerns must be fully addressed and that acceptance of the manuscript would entail a second round of review. I should remind you that it is Molecular Systems Biology policy to allow a single round of revision only and that, therefore, acceptance or rejection of the manuscript will depend on the completeness of your responses included in the next, final version of the manuscript. For this reason, and to save you from any frustrations in the end I would strongly advise against returning an incomplete revision and would also understand your decision if you choose to rather seek rapid publication elsewhere at this stage.

Should you find that the requested revisions are not feasible within the constraints outlined here and choose, therefore, to submit your paper elsewhere, we would welcome a message to this effect.

On a more editorial level, we would ask you to address the following issues.

REFEREE REPORTS

Reviewer #1:

This is an interesting paper on a very important topic that, however, exhibits several shortcomings that would benefit from correction.

From the abstract or title of the paper one would not know that the paper deals with the neuromuscular junction of Drosophila. It is annoying that the abstract and title doesn't allow any conclusions about what was actually done in the paper. This fits a general pattern in science nowadays. Titles are often sweeping even though the actual study is thankfully more focused. I think the title and abstract of the paper should state what is actually done: RNAseq on motoneurons of Drosophila.

Technically, the number of cells sequenced (999) and the number of genes transcripts per cell (1200) that were identified is low. Moreover, it appears that only 7 cells per type of motoneuron were sequenced. Because the sequencing depth was so low, the authors had to amplify homeobox gene transcripts that they would otherwise not have seen, if I understand the paper correctly. Overall, the RNAseq data are thus preliminary and do not match the standards of the field.

The paper has a tendency to overinterpret and overclaim. The authors not only claim that their RNAseq results are comprehensive, which they don't seem to be to me, but they make also other major claims that are not supported by the data. For example, the paper concludes that different motoneurons are specified by combinations of homeobox genes, but the RNAseq data themselves do not even define such combinations. Moreover, to make such a claim one would actually have to perform comprehensive manipulations of the homeobox genes. Similarly, the paper claims to show that homeobox gene expression combinations encode synaptic specificity by directing expression of Ig-domain proteins. But there is no real evidence that Ig-domain proteins mediate synaptic specificity, and certainly not in this paper. The phenotypes of Dpr and DIP mutants reported by others are rather weak, and the current paper doesn't really add to that.

In summary, this paper deals with an interesting problem, uses a potentially important approach, but is rather preliminary and needs major revisions in order to be acceptable for publication.

Reviewer #2:

The molecular logic of synaptic wiring at the single cell level
Velten et al. provides a deep transcriptomic characterization of Drosophila larval motoneurons at late embryonic stages when they have begun synapse formation with their target muscles. Through a clever modification of the Smart-Seq2 protocol, they are able to accurately map these transcriptomes across the anterior-posterior body axis using Hox gene expression as a reference. The authors divide the largest of the 3 subgroups of motoneurons (twitlow) into 60 groups which they claim are defined by unique combinations of homeodomain transcription factors. In at least three examples, they are able to show that these groups correspond to individual neurons.
They then show that 4 homeodomain transcription factors expressed by the motoneuron MN2a, as well as 3 Ig proteins likely regulated by these transcription factors are required for the synaptic specificity of this neuron. The finding that homeodomain transcription factors (or any transcription factor for that matter) are important for the identity of motoneurons is not surprising, nor is the finding that Ig proteins are important for correct muscle targeting, as has already been shown in the original Dpr/DIP paper (Carrillo et al. 2015). Similarly, the fact that transcription factors and cell-surface proteins are the most differentially expressed genes in neurons during synapse formation has already been reported in several systems.
The authors then use these findings to claim that a 'homeodomain TF code' is sufficient to confer the unique identity of motoneurons and that their effects on synaptic specificity is mediated by an 'Ig code' downstream of these TFs. Unfortunately, the evidence presented by the authors are not sufficient to demonstrate either of these claims. Despite several interesting findings, this manuscript contains several unsubstantiated claims that can be misleading for readers who are not experts.
Major Issues:

• It is completely unclear how the 60 groups of twitlow motoneurons were acquired. As the initial unsupervised clustering of the cells generated only 3 clusters, one should assume that this has been done in a supervised or semi-supervised manner based specifically on homeodomain TF expression. However, details about this are not provided, even in the Methods section. The authors attempt no statistical analyses to demonstrate that these groups (at some point they call them clusters, but this might not be accurate given that they do not appear to be the products of any 'clustering' process) represent truly homogenous groups. The authors attempt to address this issue experimentally: They show that in at least 3 cases, these groups correspond to unique neurons (Although the quality of the Hth staining in Fig. 1D is not very convincing). But in the other 3 cases (in Suppl. Fig. 3), the distinction seems to hinge on subtle differences in expression levels, rather than binary expression patterns (It is not clear why FasII, which is not a transcription factors, is part of this characterization). These differences are hard to justify in the absence of any real quantification and statistical testing of significance. As such, it is more likely that the non-homeodomain transcription factors that are not considered by the authors determine the differences between those 'clusters'. The authors' specific focus on homeodomain transcription factors seems to be justified only by a GO/SMART enrichment analysis on the genes driving the first three principal components, which appears arbitrary, and by the recent C. elegans paper by Reilly et al. It is important to note that, in that paper, the authors showed that binary expression patterns of homeodomain TFs were enough to define the worm neurons, which does not seem to be the case here.
• Even if every one of these 60 motoneuron groups corresponded to unique neurons, which as discussed above is far from clear,

the authors do not present enough evidence to support their claim that "a homeo-code acts as main determinant for transcriptional heterogeneity during the wiring phase of motoneurons": This is an argument of sufficiency. While they show that all 4 homeodomain proteins expressed by MN2a neurons are required for their proper targeting, a demonstration of sufficiency would require a conversion to another recognizable cell type when this "homeo-code" is accordingly modified. Some of the data hint in that direction: Dfd knockdown in MN2a neurons leads to their axons targeting to LR, which is where MN2c neurons that do not express Dfd normally target. The authors must show with sparse labeling that the entire morphology of the MN2a neuron, including its dendritic arbor in VNC, has shifted to be like MN2c in this knockdown. They would need to show this in one more than one type of neuron to support their ambitious "homeo-code" hypothesis.
• The authors then follow with the claim that Ig cell-surface proteins DIP/Dprs mediate the effects of homeodomain TFs on synaptic specificity. While they do not explicitly state that Ig proteins must mediate all such effects, their reference to a "simple homeo-immunoglobulin-code" implies yet another unsubstantiated argument of sufficiency. The authors make a point that DIP-k knock-down phenocopies Dfd knock-down, which is interesting. They should test whether overexpression of DIP-k and DIP-gamma rescues the Dfd knock-down phenotype. Without such an experiment, the authors cannot rule out the possibility that other types of cell-surface molecules may also be important for the targeting of MN2a axons. This is again evidence based on only one cell type which the authors are attempting to generalize to all motor neurons.
Minor Issues:
• In one place the authors state that twithigh cluster is the largest population but actually the twitlow cluster is. On that note, the lack of line, or even page numbers in the manuscript is not helpful to the reviewers.
• The authors use normalized data in their heatmaps, which makes them very hard to interpret. The standard practice in the field is to use scaled and mean-centered expression values for such plots, given that the range of expression can be very variable across different genes.

Reviewer #3:

The combinatorial action of transcription factors is thought to provide a core function in providing identity of differentiating neurons. The Hox code is hereby critical to define segmental identity along the AP axis.
The current manuscript addresses a core question related to this topic: How do TFs allow for appropriate wiring.
The authors generate a FACS based single-cell RNAseq data set on motoneurons of the late embryonic Drosophila nervous system. They use this information to identify specific neural subtypes in silico and map them in vivo. They further focus on the role of the Hox gene Dfd and its role in muscle targeting of motonneurons.

The study is quite elegant, and the results are very well presented. The question is timely and using sc-data to study axon targeting in the respective system novel.

There are a few comments that I feel might be important to address:

- One core conceptual point that I really missed was that "segmental identity" is almost completely ignored in the study of the developmental part of the study. Is the mis-targeting phenotype simply an interreference with terminal differentiation or does the neuron alter it's identity? For instance, does gain/loss of one hox in a specific neuron cause the neuron to target the muscles of a more anterior or posterior segment. Is this both in loss and gain the case according to the classic Hox model?
Or are the phenotypes observed merely due to a generic malformation.

- Similarly, while the description of the sc-data is quite well elaborated, the actual developmental study remains a superficial. The unique readout of the developmental fate is the proper targeting of a specific muscle (mouth hook elevator muscle; MHE) of a specific neuron (MN2a), which is likely disturbed by various genetic manipulations. There are a few quite big gaps in the analysis.
For instance, any temporal requirement is completely ignored. The authors argue that it's a late phenotype, but it could as well be an early one as there is no proper time-course experiment nor are temporally distinct drivers used.
Similarly, if it was not assessed if there is a change in a cell-fate of MN2a on the level of other markers.

-In principle also the generality of the observed targeting could have been addressed, at least at the level of using the experimental gain-of-function and knockdown of Hox genes and DIP. Do other MNs show similar phenotypes; (e.g. gain of Ubx should not affect posterior MNs, while anterior ones should be affected?).

- "In total, 1536 motoneurons were sequenced by SMART- Seq2 (Picelli et al., 2014). After filtering, 999 single-cell transcriptomes were retained, thus each biologically unique motoneuronal cell was covered on average 7-fold in our dataset (Supplementary Fig. 1B)"
It is unclear with the method section if this is simply the cut-off of reads/genes or if other filters have been used since about 1/3 of cells are lost. Why is this the case?
Maybe it's worth to state this in the main text.

- Do VUMs also follow the AP Hox expression code? It might be interesting to also display (and putatively discuss) this data.

POINT-BY-POINT RESPONSE TO THE REVIEWERS:

We would like to thank all the reviewers for their constructive criticism und helpful suggestions, which have helped to improve the manuscript significantly. We have included many new data, have performed new analyses and have substantially re-written the manuscript, which we describe in detail below.

Individual comments to the reviewers' questions:

Reviewer #1: thought the study is interesting on a very important topic. However, this reviewer also thought that the manuscript would benefit from some corrections before publication in MSB. We addressed the comments of this reviewer in the following way:

1) **From the abstract or title of the paper one would not know that the paper deals with the neuromuscular junction of Drosophila. It is annoying that the abstract and title doesn't allow any conclusions about what was actually done in the paper. This fits a general pattern in science nowadays. Titles are often sweeping even though the actual study is thankfully more focused. I think the title and abstract of the paper should state what is actually done: RNAseq on motoneurons of Drosophila.**

We appreciate the reviewer's thoughts on this matter and have renamed the paper to:
The molecular logic of synaptic wiring of Drosophila embryonic motoneurons at the single cell level
We have additionally rephrased the abstract to include the model organism and explain the experimental approaches. We stress that our manuscript uses various lines of evidence (imaging, genetics, single-cell RNA-seq) to draw conclusion.

2) **Technically, the number of cells sequenced (999) and the number of genes transcripts per cell (1200) that were identified is low. Moreover, it appears that only 7 cells per type of motoneuron were sequenced. Because the sequencing depth was so low, the authors had to amplify homeobox gene transcripts that they would otherwise not have seen, if I understand the paper correctly. Overall, the RNAseq data are thus preliminary and do not match the standards of the field.**

We believe the quality of our data was insufficiently explained and compared in the previous version of the manuscript, and we would like to use this opportunity to clarify.

First, the number 1200 refers to the number of unique genes observed per cell. This quantity depends on the protocol used and on the cell type. To help put this number in context, we have created a boxplot of the number of genes observed per cell in this study, as well in several reference studies working with similar biological systems (see ***new Figure EV1F****). These analyses demonstrate that our data quality, in terms of depth, matches the standard in the field. We further point out that we have sequenced the single cells to a median depth of 340,803 aligned reads per cell, which is typical for Smart-Seq2 studies but much deeper than what is typically done in single-cell RNA-Seq (see* ***new Figures EV1E, EV1F****). The number 'gene transcripts per cell', as referenced by the reviewer, cannot be computed from Smart-Seq2 data, since no unique molecular identifiers are included in this protocol.*

Further, we point out that Smart-Seq2 is the most sensitive single cell protocol according to a recent standardized comparison (Ziegenhain et al., 2017)*, i.e. it is the protocol most sensitively detecting transcripts. We here used a standardized and optimized setup of Smart-Seq2* (Velten et al., 2017, 2021)*. The addition of targeting primers to this setup increases sensitivity further beyond the already high baseline sensitivity of Smart-Seq2. This modification was developed by* (Giustacchini et al., 2017) *and further* (Velten et al., 2021)*. We have now correctly cited these references and expanded the discussion in the results on data quality.*

We disagree with the statement that 7 cells per type of motoneuron were sequenced. All 758 twil^{low} motoneurons form a largely homogeneous cluster. 7 biological replicates were sequenced ***per biologically unique cell;*** *in total, there are only about 140 OK371+ motoneurons at this stage of*

*development, corresponding to 70 distinct biological identities due to the bilateral symmetry (see **revised Figures EV1A, EV1C**). 999 cells were sequenced, corresponding to a 7-fold coverage of biologically unique cells and a 14-fold coverage when taking bilateral symmetry into account. To our knowledge, this is among the first single-cell RNA-seq study where biologically unique cells were covered in replicates; usually only a small fraction of cells in a given tissue is covered in single-cell RNA-seq studies.*

The total number of cells included here (999) may appear low compared to whole organism cell atlases with 10,000s of cells, but since the focus here is a very specific cell type present at around 140 cells per organism, it is actually a large excess. Cell numbers in the low 1000s remain the standard for specifically characterizing the heterogeneity of individual cell types by single cell RNA-seq (Davie et al., 2018; Li et al., 2017).

3) **The paper has a tendency to overinterpret and overclaim. The authors not only claim that their RNAseq results are comprehensive, which they don't seem to be to me, but they make also other major claims that are not supported by the data. For example, the paper concludes that different motoneurons are specified by combinations of homeobox genes, but the RNAseq data themselves do not even define such combinations.**

First of all, we agree that the use of 'specify' goes beyond the definition of combinations of homeodomain TF expression (which is used in bioinformatics) and implies that factors specify the cell identity in early development (in biology). We apologize for this mixing of terms, and to avoid confusions between 'specification of cellular identity in early embryonic development' and the intended meaning, 'control/determine specificity in the establishment of neuronal wiring', we now avoid the term specify altogether, for example in the abstract:

'*We show that a highly specific combinatorial code of homeodomain transcription factors and downstream Immunoglobulin domain proteins is expressed in individual cells and plays an important role in determining cell specific connections between differentiated motoneurons and target muscles.'*

*Importantly, we would like to emphasize here that our experimental set-up of transgene expression in the neuronal system by means of the elav-GAL4 driver leaves the initial specification of neurons unaffected, as this GAL4 driver induces transgene expression when the neurons are already differentiating. Thus, we only interfere with the late phase of development, in particular the wiring with target cells. This is now made clear in the manuscript. To prove this point, we also included new data in **Figure EV5A**, showing that RNAi induced reduction of gene expression using this driver is first visible in embryonic stage 14 when neurons are post-mitotic and differentiating.*

*As outlined in response to the reviewer's first comment, our scRNA-Seq data sufficiently cover all biologically unique cells in the system and match the current standard in the field. Based on this dataset, we defined combinations of homeobox gene expression and validated them in some cases by microscopy. We now list all combinations (defined from RNA-seq) in the **new Appendix Table S1**. We have further made clear that these combinations were identified bioinformatically in an unsupervised manner (see detailed response to reviewer 2's second point below).*

*To further validate this homeo-code code, we made use of the stereotypic position of some anterior motoneurons that innervate muscle groups required for feeding, which has been described in part in a previous publication form the lab (Friedrich et al., 2016). To unambiguously identify these motoneurons, we first localize the MN2a motoneuron, which reproducibly innervates the mouth hook elevator (MHE) muscle. To this end, we performed a co-staining of FasII and Myosin to label the muscles and the axon projections, or alternatively a FasII and Dfd co-staining. Due to the typical shapes of the MHE muscle and the axon projection emerging from the MN2a, this motoneuron can be reproducibly and confidently identified, as it projects an axon from a small group of Dfd expressing neurons (located within the SOG) to the MHE muscle (**new main Figure 2C**). Based on the location of the MN2a, the two neighboring neurons (MN2c and MN4) can be equally well and reproducibly identified. And finally, we show in the revised version of the manuscript that also a Scr expressing motoneuron innervating the mouth hook depressor (MHD) muscle can be highly reproducibly identified (MN3, **new main Figure 2C**). In total, we can identify 4 anterior neurons based solely on their stereotypic localization.*

For the revised manuscript, we have now analysed the expression of eight homeodomain encoding genes, mirr, Dfd, Scr, lab, vvl, Lim1, hth and unc-4, which we found by scRNA-Seq to be co-expressed in the different anterior motoneurons. To be able to do so, we have established a new technique in the lab, ***Hybridization Chain Reaction (HCR)*** *version 3.0 protocol (Choi et al., 2018), as for some of the genes we needed to study in this manuscript antibodies are* ***unavailable****. Based on the expression pattern of these eight genes, we were able to unambiguously assign all four motoneurons to clusters defined by the transcriptome data (MN2c = C6, MN2a = C18, MN4 = Cluster C12, MN3 = C20). The analysis is now described in the Results part of the manuscript and in detail in the Materials and Methods section as well as in* ***revised main Figure 2****. As described in response to the reviewer's next point, genetic experiments and imaging of MN2a and MN3 axon projections provided further validation of the homeo-code: ectopic expression of homeodomain TFs caused phenotypic defects in the motoneuron where these factors are not expressed, whereas knockdown via RNAi caused phenotypic defects in the motoneuron we found to express the TF (see also* ***revised main Figures 3 and new main Figure 4****). See more details in response to point 4.*

Together these analyses confirm that the RNA-seq dataset can define the combinations of homeodomain TFs expressed in single neurons or small groups of neurons. We have added a more critical discussion on the limitations of this approach:

"While difficulties in microscopically identifying all individual 60 MNs impeded us from unanimously designating all clusters as biologically unique cells, our analyses demonstrated that a homeodomain TF 'code' is associated with cellular heterogeneity during the synaptic wiring phase, and is specific to small groups of cells or even single cells." (Results, page 9)"

and

"*Despite the use of a high number of biological replicates for each unique cell, scRNA-Seq on its own cannot be applied to unanimously identify biologically unique cells. Thus, we used imaging to demonstrate in four cases a correspondence between homeo-clusters identified by scRNA-Seq and gene expression patterns in stereotypically defined single cells. Together, these data allowed us to conclude that a homeo-code is associated with cellular heterogeneity in Drosophila MNs, and is specific to small groups of cells or even single cells.* "*(Discussion, page 13)*

4) **Moreover, to make such a claim one would actually have to perform comprehensive manipulations of the homeobox genes.**

We thank the reviewer for this very useful suggestion. We have expanded our functional study to more systematically investigate the role of homeodomain TFs on motoneuronal wiring (see ***revised main Figures 3 and new main Figure 4****). In unperturbed development, from anterior to posterior, the Dfd expressing motoneuron MN2a innervates the Dfd positive muscle MHE and the Scr expressing motoneuron MN3 innervates the Scr positive muscle MHD (shown in* ***new main Figure 4A****). We have now done the following manipulations, the results from these functional interference and mis-expression studies support our claim that homeodomain TF combinations are active in specific motoneurons and control their wiring specificity:*

- *Ectopic expression of the Hox class homeodomain TF Scr causes the MN2a (Dfd+) motoneuron to now innervate the posterior MHD muscle, as expected, but only induces a mild phenotype in MN3 (Scr+) (see* ***new main Figures 4B, 4C****)*
- *Ectopic expression of the Hox class homeodomain TF Dfd causes the MN3 (Scr+) motoneuron to innervate to the anterior MHE muscle, but only induces a mild phenotype in the MN2a (Dfd+) (see* ***new main Figures 4B, 4C****)*
- *Dfd knockdown causes a branching phenotype in MN2a with a tendency to innervate the anterior LR muscle, which normally expresses the Hox gene labial (lab) (see* ***revised main Figures 3D, 3E****)*
- *Ectopic expression of Hox class homeodomain TF Lab causes the Dfd+ MN2a motoneuron to innervate the anterior LR muscle (Lab+) (see* ***new main Figures 4B, 4C****)*

Interestingly, the expression of the homeodomain encoding gene mirr is also highly specific for MN2a and MHE, whereas the homeodomain encoding gene hth is expressed by all muscle and motoneurons in that region (see ***revised main Figures 2, 3****). Accordingly, knockdown of mirr causes a mild*

branching phenotype and a tendency to target the Scr+/mirr- muscle located below, whereas hth knockdown causes an extreme change in wiring to a different region.

This confirms that the homeodomain TFs lab, Dfd, Scr, hth and mirr can change the wiring phenotype of two distinct MN projections (MN2a and MN3), and that the phenotypic consequences of these genetic manipulations justify a model whereby a specific combination of homeodomain transcription factors identified by our single cell RNA-seq data control neuronal wiring in a cell-specific manner.

5) **Similarly, the paper claims to show that homeobox gene expression combinations encode synaptic specificity by directing expression of Ig-domain proteins. But there is no real evidence that Ig-domain proteins mediate synaptic specificity, and certainly not in this paper. The phenotypes of Dpr and DIP mutants reported by others are rather weak, and the current paper doesn't really add to that.**

We have added new experiments further supporting a role of DIPs as regulators of synaptic specificity. To this end, we ectopically expressed DIPgamma and monitored the effect on the Scr expressing MN3 motoneuron, which according to our single cell RNA sequencing data does not express DIPgamma. We found a very clear shift towards the upper MHE (Dfd +) that is normally innervated by the DIPgamma expressing MN2a (Dfd +) in wild-type embryos (see ***revised main Figure 6****). Importantly, a similar phenotype is observed when Dfd is ectopically expressed in MN3 (see* ***new main Figure 4****). Together with the weaker phenotype of DIPkappa and DIPgamma knockdowns (which phenocopy the effect of Dfd knockdown on MN2a) that we have already reported in the initial version of the manuscript, this experiment provides further support for our statement that DIPgamma is a regulator of synaptic specificity downstream of Dfd (see* ***revised main Figure 6****). Additionally, we added ectopic expression of other immunoglobulin genes, such as dpr1 and dpr11 that are both absent in MN2a and MN3, but present in many other MN clusters. Ectopic expression of these factors also results in wiring defects (see* ***revised main Figure 6****).*

To further validate the claim that homeodomain TFs regulate DIP/Dprs, we performed two independent approaches, qPCR and ***HCR*** *experiments (HCR was established for this purpose to be able to monitor the expression of various Ig and homeodomain genes in the embryo). In both set ups, we interfered with the homeodomain TF Dfd in the nervous system and evaluated the downregulation of DIPgamma and DIPkappa compared to wild-type embryos. These new experiments are shown in* ***revised main Figure 5*** *and confirm the regulatory effect of homeodomain TFs on selected Immunoglobulin genes.*

We agree that our results cannot exclude that besides immunoglobulins other factors might play a role in mediating this process. We therefore changed the wording in the abstract and main text to: *"Taken together, our data strongly suggest that a linked homeo-immunoglobulin-code is a major determinant of neuronal circuit structure."*

Reviewer #2: highlighted that the study provides a deep transcriptomic characterization of Drosophila larval motoneurons at late embryonic stages and accurately maps these transcriptomes across the anterior-posterior body axis using Hox gene expression as a reference. However, the reviewer also commented that the manuscript contains some unsubstantiated claims that can be misleading for readers. We addressed the comments of this reviewer in the following way:

1) **The finding that homeodomain transcription factors (or any transcription factor for that matter) are important for the identity of motoneurons is not surprising, nor is the finding that Ig proteins are important for correct muscle targeting, as has already been shown in the original Dpr/DIP paper (Carrillo et al. 2015). Similarly, the fact that transcription factors and cell-surface proteins are the most differentially expressed genes in neurons during synapse formation has already been reported in several systems. The authors then use these findings to claim that a 'homeodomain TF code' is sufficient to confer the unique identity of motoneurons and that their effects on synaptic specificity is mediated by an 'Ig code' downstream of these TFs. Unfortunately, the evidence presented by the authors are not sufficient to demonstrate either of these claims.**

We agree with the reviewer that homeodomain TFs are important for the establishment of the identity of motoneurons. The novelty here lies in the investigation of homeodomain TFs mediated ***effects*** *in postmitotic motoneurons at the single cell level, a rather understudied topic (cf. Hobert, Nature Reviews Neuroscience 2021). The technical advances of this study allowed us to perform an unbiased analysis of co-varying expression patterns of potential transcriptional regulators and downstream molecules. Here we find that in late stages of motoneuron development the major process associated with the homeodomain TF code was the differential expression of Ig domain molecules. As the reviewer stated Ig domain molecules are well known to be implicated in synaptic connectivity. However, neither the link between homeodomain TFs and Igs in postmitotic MNs was clear, nor the specificity mediated by these factors for muscle target selection.*

We thank the reviewer for this comment regarding sufficiency and would like to highlight that we do not attempt to state that the homeodomain code is the only ('sufficient') regulator of Ig expression, or that Ig expression is the only regulator of synaptic specificity. However, we do think that our manuscript provides strong evidence that homeodomain TFs play an (important) role in regulating Ig genes, and that those in turn play an (important) role in regulating synaptic specificity. While we below address the specific points of this reviewer in detail, with new experiments, we have also rephrased the abstract as follows (and the main text) to avoid potentially misleading claims.

"Here, we investigated Drosophila embryonic motoneurons using single cell genomics, imaging and genetics. We show that a highly specific combinatorial code of homeodomain transcription factors and downstream Immunoglobulin domain proteins is expressed in individual cells and plays an important role in determining cell specific connections between differentiated motoneurons and target muscles. We provide evidence for a functional role of five homeodomain transcription factors (Dfd, Hth, Mirr, Scr, Lab), and four immunoglobulins (DIP-kappa, DIP-gamma, Dpr1, Dpr11) in the wiring of neuronal circuits."

2) **It is completely unclear how the 60 groups of twitlow motoneurons were acquired. As the initial unsupervised clustering of the cells generated only 3 clusters, one should assume that this has been done in a supervised or semi-supervised manner based specifically on homeodomain TF expression. However, details about this are not provided, even in the Methods section. The authors attempt no statistical analyses to demonstrate that these groups (at some point they call them clusters, but this might not be accurate given that they do not appear to be the products of any 'clustering' process) represent truly homogenous groups. The authors attempt to address this issue experimentally: They show that in at least 3 cases, these groups correspond to unique neurons (Although the quality of the Hth staining in Fig. 1D is not very convincing). But in the other 3 cases (in Suppl. Fig. 3), the distinction seems to hinge on subtle differences in expression levels, rather than binary expression patterns (It is not clear why FasII, which is not a transcription factors, is part of this characterization). These differences are hard to justify in the absence of any real quantification and statistical testing of significance. As such, it is more likely that the non-homeodomain transcription factors that are not considered by the authors determine the differences between those 'clusters'. The authors' specific focus on homeodomain transcription factors seems to be justified only by a GO/SMART enrichment analysis on the genes driving the first three principal components, which appears arbitrary, and by the recent C. elegans paper by Reilly et al. It is important to note that, in that paper, the authors showed that binary expression patterns of homeodomain TFs were enough to define the worm neurons, which does not seem to be the case here.**

We agree with the reviewer and to make this point clearer, we have now rearranged our manuscript to clarify how the 60 clusters were obtained. They are exclusively based on the expression of the homeodomain TF genes as the reviewer mentioned. However, the clustering on homeodomain TFs were obtained in an unsupervised manner; both binary and gradual differences shape this code. We have expanded the Materials and Methods section to explain this more precisely; and we provide further data and analyses regarding the experimental validation of individual motoneurons by imaging of further homeodomain factors, as well as genetics.

In summary,
- *We first show that homeodomain genes, unlike other gene classes, have a very high biological variability within the population of twi^{low} cells (this was done using the statistical method by*

(Brennecke et al., 2013)*, and formerly shown in the supplement, but now included as* ***revised main Figure 1C****)*

- *We then show that this variability is not 'random': first of all, it is associated with the first PCs as mentioned by the reviewer; second, it is associated with spatial heterogeneity (see revised* ***main Figures 1D, 1E****); third, even when accounting for spatial heterogeneity, homeodomain genes remain highly variable (****revised main Figure 1E****,* ***EV4H****), according to the statistical method by* (Risso et al., 2019)
- *Together, these lines of statistical evidence suggest that homeodomain genes can be used to sub-structure the* twi^{low} *cells. Therefore, we performed hierarchical clustering using only homeodomain genes as input. Thereby, we effectively avoid that this clustering is dominated by technical noise (a control for this is shown in* ***Figure EV4H****). The strategy to perform clustering using specific sets of transcription factors is commonly used when dissecting the heterogeneity of rather homogeneous cell populations by single-cell RNA-seq, see for example* (Li et al., 2017)
- *A further statistical justification of the exact number of clusters is difficult and typically remains an ad hoc choice. This is widely recognized in the literature* (Luecken & Theis, 2019; Zhu et al., 2019)*. To achieve a certain statistical support for their choice of cluster number, authors in the field have used the gap statistics (for example,*(Grün et al., 2015; Velten et al., 2017)*), average silhouette width* (Peyvandipour et al., 2020)*, measures of intra-cluster dispersion (Grün et al., 2016) and various Bayesian approaches (e.g.*(Azizi et al., 2017)*). For our dataset, these methods would statistically justify a very large number of clusters (~200) exceeding the number of biologically unique cells included in the experiment, suggesting that they inappropriately account for technical noise (****Response Figure 1****). We therefore chose the number 60, since it approximately corresponds to the number of biologically unique* twi^{low} *neurons labeled by the OK371-Gal4 driver during this stage (see revised* ***Figure EV1A, EV1C****) and produces visually rather homogeneous clusters that can be arranged along the AP-axis. We cannot exclude that we thereby do not achieve a 1:1 correspondence to the true biologically distinct cells. This is now mentioned more clearly in the manuscript (page 8).*

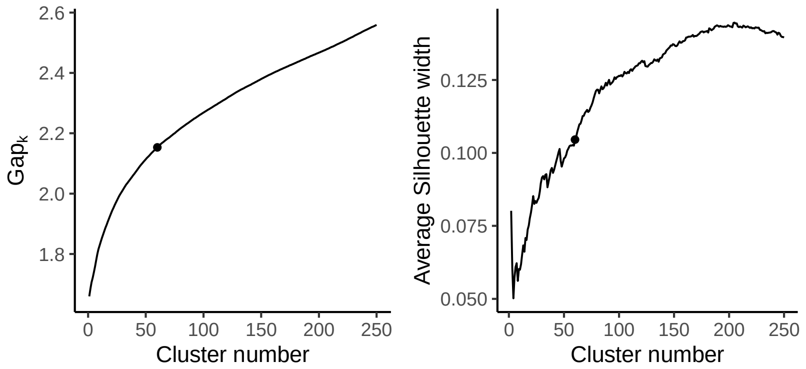

Response Figure 1: *Line plots depicting the gap statistics and the average silhouette width as a function of cluster number. The black dot indicates the cluster number used for the analysis in the manuscript.*

- *To further validate the code, we made use of the stereotypic position of some cells. We are able to reproducibly identify the MN2a motoneuron based on its connection to the mouth hook elevator (MHE) muscle; going from there, we identify the two neighboring neurons, MN4 and and MN2c. In the revised version of the manuscript, we have explained our strategy to identify these neurons in more detail and added a figure (new Figure 2C). We now further show that we can equally well identify a Scr expressing motoneuron innervating the mouth hood depressor (MN3, new main* ***Figure 2****). In total, we can identify 4 neurons based only on their stereotypic localization. In the revised manuscript, we have now analysed the expression of 8 homeodomain encoding genes, mirr, Dfd, Scr, lab, vvl, Lim1, hth and unc-4, which we found by scRNA-Seq to be co-expressed in the different anterior motoneurons. To be able to do so, we have established a new technique in the lab,* ***in situ HCR*** *version 3.0 protocol (Choi et al., 2018), as for some of the genes we needed to study in this manuscript, antibodies were* ***unavailable****. Based on the expression pattern of these eight genes, we were able to unambiguously assign all four motoneurons to clusters defined by the transcriptome data (MN2c = C6, MN2a = C18, MN4 = Cluster C12, MN3 = C20). Hth is not required for making this assignment however it is clearly expressed by most motoneurons in that region, in line with the RNA-seq result. The analysis is now described in the Results part of the manuscript and in detail in the Materials and Methods section as well as in* ***revised main Figure 2****.*

- *As described in detail in the context of **revised main Figure 3** and **new main Figure 4**, genetic experiments and imaging of MN2a and MN3 axon projections provide further validation for the homeo-code: Ectopic expression of homeodomain TFs caused phenotypic defects in the neuron where these factors are not expressed, whereas knockdown via RNAi caused phenotypic defects in the neuron we found to express the TF.*

- *In the posterior segment of the Drosophila embryo (as opposed to larva), it is for us not possible to identify motoneurons based on their location. According to our transcriptome data, the unique identification of Ubx+ motoneurons by binary expression patterns of homeodomain TFs would require a co-staining of ~11-14 markers (see **Response Figure 2** below); at the low level of multiplexing that is technically feasible, we did observe quantitative differences as predicted from the transcriptome experiment, but no binary pattern, as expected. This limitation was made clear in the manuscript (page 9) and the data on Ubx expressing motoneurons was removed.*

*Taken together, single cell RNA-seq (**revised main Figure 2**), imaging (**revised main Figure 2**) and genetics (revised **main Figure 3 and new main Figure 4**) support the view that "a homeodomain TF 'code' is associated with cellular heterogeneity during the synaptic wiring phase, and is specific to small groups of cells or even single cells" (page 9).*

	33	34	35	36	37	38	39	40	41	42	43	44	45	46	47	48	49	50	51	52	54	58
Ubx	+	+	+	+	+	+	+	+	+	+	+	+	+	+	+	+	+	+	+	+	+	+
mirr	+		+	+	+	+					+			+	+						+	+
vvl	+		+	+		+					+						+		+		+	+
Lim1		+		+	+	+	+	+	+		+		+	+	+	+		+	+	+	+	+
Antp	+	+	+	+		+	+		+	+	+	+	+	+	+	+		+	+	+	+	+
Abd-A	+				+	+				+	+	+	+	+	+	+	+	+	+	+	+	+
Abd-B	+							+										+			+	+
zfh2	+	+	+	+		+		+		+		+	+	+		+	+	+	+	+		+
Dr		+		+					+							+		+	+			
pb										+						+						
unc4																			+			

***Response Figure 2**: A Homeocode defines twitl^{ow} motoneurons*
Binarized visualisation of all Ubx expressing motoneuron clusters and the required homeo-code to uniquely distinguished the clusters by the expression of the 11 here depicted homeodomain TFs. Magenta = no expression, green = expression.

3) **Even if every one of these 60 motoneuron groups corresponded to unique neurons, which as discussed above is far from clear, the authors do not present enough evidence to support their claim that "a homeo-code acts as main determinant for transcriptional heterogeneity during the wiring phase of motoneurons": This is an argument of sufficiency. While they show that all 4 homeodomain proteins expressed by MN2a neurons are required for their proper targeting, a demonstration of sufficiency would require a conversion to another recognizable cell type when this "homeo-code" is accordingly modified. Some of the data hint in that direction: Dfd knockdown in MN2a neurons leads to their axons targeting to LR, which is where MN2c neurons that do not express Dfd normally target. The authors must show with sparse labeling that the entire morphology of the MN2a neuron, including its dendritic arbor in VNC, has shifted to be like MN2c in this knockdown. They would need to show this in one more than one type of neuron to support their ambitious "homeo-code" hypothesis.**

First of all, we agree that the use of 'specify' would go beyond the definition of combinations of homeodomain TF expression (which is used in bioinformatics) and implies that factors specify the cell identity in early development (in biology). We apologize for this mixing of terms, and to avoid confusions between 'specification of cellular identity in early embryonic development' and the intended

meaning, 'control/determine specificity in the establishment of neuronal wiring', we now avoid the term specify altogether, for example in the abstract:

'We show that a highly specific combinatorial code of homeodomain transcription factors and downstream Immunoglobulin domain proteins is expressed in individual cells and plays an important role in determining cell specific connections between differentiated motoneurons and target muscles.'

The focus of this study is not the specification of neuronal identity, but the establishment of specific motoneuron-muscle connections. That is also the reason why we used the experimental set-up of transgene expression in the neuronal system by means of the elav-GAL4 driver, as this GAL4 driver induces transgene expression when the neurons are already differentiating, thereby leaving the initial specification of neurons unaffected. Thus, we only interfere with the late phase of development, in particular the wiring with target cells. This is now made very clear in the manuscript. To prove this point, we also included new data in ***Figure EV5A****, showing that RNAi induced reduction of gene expression using this driver is first visible in embryonic stage 14 when neurons are post-mitotic and differentiating.*

This is also the main reason why we did not study the morphology of the entire motoneuron, in particular the dendritic arbors (which is also not the scope of this paper). Due to our experimental set-up, the identity of the motoneuron will not change, as we only interfere with the "late" program, meaning when the neurons are already specified and differentiating. Indeed, our experiments very clearly show that these late events can be changed depending on the Hox gene expressed. This is in line with previous findings showing that Hox genes control (moto)neuronal development in a stage specific manner (Friedrich et al., 2016; Philippidou et al., 2012), *meaning they control different target genes at the different stages, in our case genes critical for wiring. Furthermore, in contrast to the larval neuromuscular system, high-resolution imaging of synapses and dendritic arbors is not possible in the Drosophila embryo.*

As suggested by the reviewer, we have expanded our functional study to more systematically investigate the role of homeodomain TFs on motoneuronal wiring (see ***new main Figure 4****), which is a phenotype of differentiated motoneurons that can be studied in Drosophila embryos and which we used as read-out for synaptic wiring specificity.*
We now compare the muscle targeting of two distinct axon projections, the one from MN2a and the one from MN3: In unperturbed development, the axon projecting from the Dfd expressing MN2a innervate the Dfd expressing MHE muscle, while the axon projecting from the Scr expressing MN3 innervates the Scr expressing MHD muscle (shown in ***new main Figure 4A****). We have done the following manipulations, the results from these functional interference studies support our claim that homeodomain TF combinations are active in specific motoneurons and control their wiring specificity:*

- *Ectopic expression of the Hox class homeodomain TF Scr causes the MN2a (Dfd+) motoneuron to now innervate the posterior MHD muscle, as expected, but only induces a mild phenotype in MN3 (Scr+) (see* ***new main Figures 4B, 4C****)*
- *Ectopic expression of the Hox class homeodomain TF Dfd causes the MN3 (Scr+) motoneuron to innervate to the anterior MHE muscle, but only induces a mild phenotype in the MN2a (Dfd+) (see* ***new main Figures 4B, 4C****)*
- *Dfd knockdown causes a branching phenotype in MN2a with a tendency to innervate the anterior LR muscle, which normally expresses the Hox gene labial (lab) (see* ***revised main Figures 3D, 3E****)*
- *Ectopic expression of Hox class homeodomain TF Lab causes the Dfd+ MN2a motoneuron to innervate the anterior LR muscle (Lab+) (see* ***new main Figures 4B, 4C****)*

Interestingly, the expression of the homeodomain TF encoding gene mirr is also highly specific for MN2a and MHE, whereas the homeodomain TF encoding gene hth is expressed by all muscle and motoneurons in that region (see ***revised main Figures 2, 3****). Accordingly, knockdown of mirr causes a mild branching phenotype and a tendency to target the Scr+/mirr- muscle located below, whereas hth knockdown causes an extreme change in wiring to a different region.*

This confirms that the homeodomain TFs lab, Dfd, Scr, hth and mirr can change the wiring phenotype of two distinct MN projections (MN2a and MN3) and that the phenotypic consequences of these genetic manipulations justify a model whereby a specific combination of homeodomain transcription factors identified by our single cell RNA-seq data control neuronal wiring in a cell-specific manner.

4) **The authors then follow with the claim that Ig cell-surface proteins DIP/Dprs mediate the effects of homeodomain TFs on synaptic specificity. While they do not explicitly state that Ig proteins must mediate all such effects, their reference to a "simple homeo-immunoglobulin-code" implies yet another unsubstantiated argument of sufficiency. The authors make a point that DIP-k knock-down phenocopies Dfd knock-down, which is interesting. They should test whether overexpression of DIP-k and DIP-gamma rescues the Dfd knock-down phenotype. Without such an experiment, the authors cannot rule out the possibility that other types of cell-surface molecules may also be important for the targeting of MN2a axons. This is again evidence based on only one cell type which the authors are attempting to generalize to all motor neurons.**

We agree that we did not make sufficiently clear that immunoglobulins are the major, but very likely not the only factors mediating this process. We therefore changed the wording in the abstract and main text to: "a linked homeo-immunglobulin-code is a major determinant for neuronal circuit structure".

To further validate the claim that homeodomain TFs regulate DIP/Dprs, we performed two independent approaches, qPCR and ***HCR*** *experiments (HCR was established for this purpose to be able to monitor the expression of various Ig and homeodomain genes in the embryo). In both set ups, we interfered with the homeodomain TF Dfd in the nervous system and evaluated the downregulation of DIPgamma and DIPkappa compared to wild-type embryos. These new experiments are shown in* ***revised main Figure 5*** *and confirm the regulatory effect of homeodomain TFs on selected Immunoglobulin genes.*

In addition, we ectopically expressed DIPgamma in neurons and show that this affects the MN3, as this motoneurons, which normally does not express DIPgamma and projects to the MHD muscle, now innervates also the MHE muscle, which is normally only innervated by projections emerging from the DIPgamma expressing MN2a (see ***revised main Figure 6****). A similar effect is observed when Dfd is ectopically expressed in neurons (see* ***new main Figure 4****), further supporting the regulatory interaction of homeodomain and Ig genes. In sum, we think that these experiments strongly support our claim that Ig genes act downstream of homeodomain TFs. However, we agree that our results cannot exclude that besides immunoglobulins other factors might play a role in mediating this process. We therefore changed the wording in the abstract and main text to: "Taken together, our data strongly suggest that a linked homeo-immunoglobulin-code is a major determinant of neuronal circuit structure."*

We thank the reviewer for the suggestion of the rescue experiment of Dfd knockdown by DIP gamma and DIP kappa overexpression. Such an experiment is not feasible in a reasonable period of time (requires creation of a new fly line, since no DIPkappa overexpression line is not available; and additionally recombining and crossing to create elav>Dfd^{RNAi},DIPgamma,DIPkappa animals). We instead expressed DIPgamma in Dfd depleted neurons (elav>Dfd^{RNAi},DIPgamma), and observed a slight non-significant increase in fraction of animals displaying the wild-type MN2a innervation pattern compared to Dfd interference conditions alone, but not a significant rescue phenotype (see ***Response Figure 3****). This result is not surprising, in particular when looking at the Ig genes expressed in individual motoneurons according to our scRNA-Seq data (shown in main* ***Figure 5C****): in MN2a, as in all other motoneurons, many Ig genes are expressed, highlighting that not a single but a combination of Ig proteins and possibly also other gene families important for synaptic specificity are regulated by Dfd. In line, we do find that interference with DIPgamma or DIPkappa resulted in lower mistargeting rates as interference with Dfd (shown in* ***revised main Figures 3 and new main Figure 6****). This is now also mentioned in the Results:*

"RNAi-mediated knockdown of DIP-kappa and DIP-gamma in neuronal cells caused the MN2a to target the LR muscle (6% and 23%, respectively) (Fig 6A, 6C), and additionally caused terminal defects (41% and 23%, respectively). Interestingly, this effect was similar to the phenotype induced by Dfd RNAi, but less pronounced (Fig 3D, 3E, 29% mistargeting and 33% terminal defects). This is consistent with the assumption that targeting specificity is mediated not by one but a combination of Ig genes acting downstream of homeodomain TFs." (Results, page 11).

Taken together, our results suggest that each Immunoglobulin has a specific function in synaptic specificity, but a single one is unlikely to be sufficient to regulate the entire process.

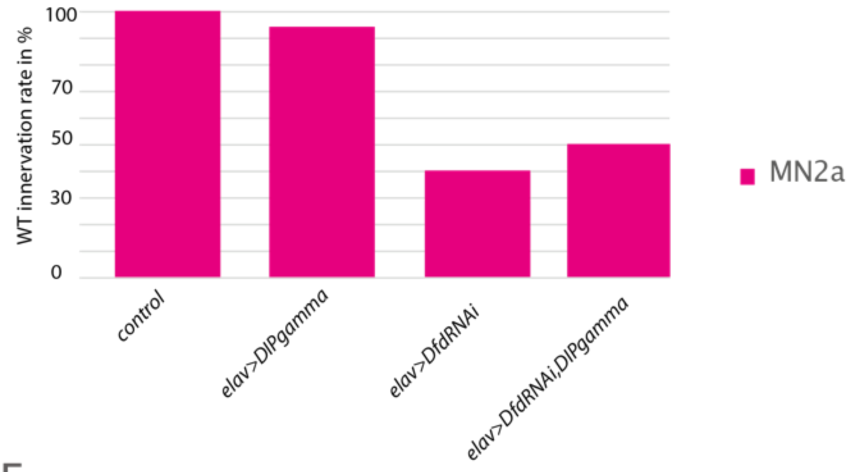

Response Figure 3: *Immunoglobulin genes are effectors of homeodomain TFs and of synaptic wiring Four independent genetic experiments using the pan-neural elav-GAL4 driver to control the expression of UAS-RNAi constructs and UAS constructs of homeodomain TFs and Immunoglobulins i. e. . elav>Dfd, elav>DIPgamma elav>Dfd^{RNAi}, elav>Dfd^{RNAi}, DIPgamma and UAS-RFP as control. Phenotypic penetrance for the MN2a projections 'innervation rate' was calculated by the rate of correct axon projections of MN2a to MHE (magenta) compared to wiring defects displayed by animals expressing RNAi constructs or UAS constructs for homeodomain TFs or Immunoglobulins. Note, each genetic experiment was performed in parallel to an adequate control experiment using the same driver line crossed to a line that controls expression of either UAS-RFP or UAS-GFP^{RNAi}.*

Minor issues:

- **In one place the authors state that twithigh cluster is the largest population but actually the twitlow cluster is. On that note, the lack of line, or even page numbers in the manuscript is not helpful to the reviewers.**

Thanks for indicating this mistake, we corrected this mistake in the main text.

- **The authors use normalized data in their heatmaps, which makes them very hard to interpret. The standard practice in the field is to use scaled and mean-centered expression values for such plots, given that the range of expression can be very variable across different genes.**

Indeed, normalized data emphasize the absolute expression level of the genes and are a meaningless quantity here. Scaled and mean-centered expression values, by contrast, take very high values if the gene is expressed in few cells and low values if the gene is expressed in many cells (see ***new Figure EV4G*** *for an example). For example, the homeodomain TF Dfd is expressed in very few motoneurons of the anterior segments, while the homeodomain TF Ubx is expressed in a large number of motoneurons of posterior segments. Scaled and mean-centered expression values would weight Dfd expression high and Ubx expression low, which does not reflect the real expression of these genes. Neither of the two strategies is ideal when aiming to identify a 'code' using clustering strategies. Binarization of the gene expression levels would constitute a viable alternative (see for example, Li et al., Cell 2017) but cannot capture biologically meaningful quantitative differences between cells expressing the gene 'highly' or 'lowly'. For the heatmap depicting the homeocode, we therefore had normalized the data gene-wise by*

$$N_i = \log\left(k\frac{X_i}{\sum X} + 1\right)$$

Where X is the vector of size factor normalized counts for a given gene. N approaches binarization for large k. Here, a value of k = 10^6 was chosen. This leads to a behavior where normalized values are essentially binarized for lowly expressed genes, where quantitative differences cannot be captured in

*a meaningful manner; by contrast, for highly expressed genes, expression levels are represented more quantitatively (see **new Figure EV4G**). We have now explained this strategy in our methods section. We have previously used similarly motivated strategy for data normalization in the context of predicting cell-cell interactions using scRNA-seq data (Baccin et al., Nature Cell Biology 2020).*

Reviewer #3: thought that the study is quite elegant, and the results are very well presented. This reviewer also highlighted that the question is timely and using sc-data to study axon targeting in the respective system novel. However, the reviewer had also few points he/she wanted to be addressed, which we did in the following way:

1) One core conceptual point that I really missed was that "segmental identity" is almost completely ignored in the study of the developmental part of the study. Is the mis-targeting phenotype simply an interreference with terminal differentiation or does the neuron alter its identity? For instance, does gain/loss of one hox in a specific neuron cause the neuron to target the muscles of a more anterior or posterior segment. Is this both in loss and gain the case according to the classic Hox model? Or are the phenotypes observed merely due to a generic malformation.

*We would like to thank the reviewer for his thoughts on this issue, and we would like to clarify a few things that were misleading in the previous version of the manuscript. First of all, we interfere late in neuronal development with gene expression by means of the UAS-GAL4 system, in particular by using the elav-GAL4 driver, which starts to induce interference with gene expression only at embryonic stage 14, when the motoneurons are post-mitotic and differentiating, (see also the reviewer's next point on temporal requirements). Thus, the neurons will not change their identities, as the early stages of development are unaffected by our mis-expression studies (see **new Figure EV5A**), and the identity of the whole segment will not be changed because we only interfere in one tissue with Hox gene expression. In contrast, by using this set-up, we specifically interfere with the late programs, meaning those processes taking place after embryonic stage 15. We monitored these changes by studying and quantifying the innervation patterns of specific motoneurons in comparison to unperturbed conditions. The results obtained from all these manipulations (see below and **revised main Figures 3** and **new main Figure 4**) show that the motoneurons innervate new muscles in a predictive manner, which is consistent with the anterior-posterior expression of Hox genes. We think that these results justify our conclusion that the observed phenotypes are not due to a generic malformation but due to highly specific manipulation of differentiation/wiring programs in motoneurons. Hox genes are known to be required throughout development and even during adulthood, and it has been shown that they control (moto)neuronal development in a stage specific manner* (Friedrich et al., 2016; Philippidou et al., 2012)*, meaning they control different target genes at the different stages. This is in line with our findings that late interference with different homeodomain transcription factors (including Hox genes) changes the late program, thereby leading to wiring defects. We have now made this point clearer in the Results part (pages 9, 10).*

*Second, we have now expanded our functional study to systematically investigate the role of Hox-TFs on motoneuronal wiring (see **new main Figure 4**). To this end, we have phenotypically characterized two motoneurons and their projections to target muscles: the Dfd expressing MN2a, which normally innervates the Dfd positive MHE muscle, and the Scr expressing MN3, which normally innervates the Scr positive MHD muscle. Both the MN2c and LR muscle express the Hox gene labial (see **new main Figure 4**). The effects that result from misexpression of the Hox TFs Lab, Dfd and Scr show that motoneurons changed their target preference in a predictive manner, which is in line with the expression of the respective Hox protein, meaning that mis-expression of more anterior Hox genes guided motoneurons to innervate more anterior muscles, while more posterior Hox genes directed the MNs to more posterior muscles (see **new main Figure 4**).*
Specifically, we found:

- *Ectopic neuronal expression of Scr causes the MN2a, which normally only expresses Dfd, to project an axon to the more posteriorly located MHD muscle, which expresses Scr. This mis-expression induces only a mild phenotype in MN3 (see **new main Figure 4)***
- *Ectopic expression of Dfd causes the MN3, which normally expresses only Scr, to project an axon to the anterior MHE muscle, which expresses Dfd. Again, only a mild phenotype is induced in MN2a (see **new main Figure 4**)*
- *Dfd knockdown causes a branching phenotype in MN2a with a tendency to innervate the anterior LR muscle, which does not express Dfd but Lab (see **revised main Figure 3**). This*

innervation pattern is phenocopied by ectopic neuronal expression of lab, as the MN2a now also innervates the LR muscle (see ***new main Figure 4****).*
This confirms that the homeodomain TFs Lab, Dfd and Scr, can change the wiring phenotype of two distinct MN projections (MN2a and MN3) and that the phenotypic consequences of these genetic manipulations justify a model whereby a specific combination of homeodomain transcription factors identified by our single cell RNA-seq data control neuronal wiring in a cell-specific manner.

2) Similarly, while the description of the sc-data is quite well elaborated, the actual developmental study remains a superficial. The unique readout of the developmental fate is the proper targeting of a specific muscle (mouth hook elevator muscle; MHE) of a specific neuron (MN2a), which is likely disturbed by various genetic manipulations. There are a few quite big gaps in the analysis. For instance, any temporal requirement is completely ignored. The authors argue that it's a late phenotype, but it could as well be an early one as there is no proper time-course experiment nor are temporally distinct drivers used. Similarly, if it was not assessed if there is a change in a cell-fate of MN2a on the level of other markers.

We have expanded the genotypes and developmental readouts included in this study, see the reviewer's previous point.

To address the reviewer's comment regarding temporal requirements, we performed additional time course experiments on the temporal control of RNAi constructs by the elav-GAL4 driver. This analysis showed that the RNAi mediated knockdown effect takes place between stage 13-14 (see ***revised Figure EV5A****). During this stage motoneurons are already specified and axonogenesis is taking place. Thus, the elav-GAL4 driver in combination with second generation TRIP line constructs such as* Dfd^{RNAi} *is ideally suited for temporal interference of proteins during synaptic wiring, as it leaves the early stages of development unaffected. We have now included these new data in the Results part and in the* ***revised Figure EV5****.*

We additionally examined the expression of other homeodomain TF genes, such as Lim1 and unc4, as well as of some Ig genes, DIPgamma dn DIPkappa, in Dfd knockdown conditions (elav>DfdRNAi). This showed that the effects of Dfd knockdown are specific, as Lim1 and unc4 expression was unaffected (see ***new Figure EV5B****), while expression of the two predicted Dfd targets, DIPgamma and DIPkappa, was reduced (see* ***new Figures 5D, 5E****). Thus, the combination of both, the elav-GAL4 time-course and the analysis of genes expressed in MN2a in control and perturbation conditions, show that the observed effects are due to late and specific interference with the motoneuronal wiring program due to interference with specific genes expressed in these motoneurons.*

3) In principle also the generality of the observed targeting could have been addressed, at least at the level of using the experimental gain-of-function and knockdown of Hox genes and DIP. Do other MNs show similar phenotypes; (e.g. gain of Ubx should not affect posterior MNs, while anterior ones should be affected?).

To address the reviewers comment we performed in depth functional experiments on homeodomain TFs and DIPs and investigated wiring defects on ***two*** *motoneuronal projections, the MN2a and MN3. In wild-type embryos, the MN2a (Dfd expressing) projects to the MHE (Dfd expressing) muscle and the MN3 (Scr expressing) projects to the MHD (Scr expressing) muscle. We then performed gain of function experiments for three different homeodomain TFs Lab, Dfd and Scr. We observed specific alterations in innervation patterns of the two axon projections MN2a and MN3. Comparison of ectopic Scr expression on the MN2a (Scr-) projection and Scr overexpression on the MN3 (Scr+) projection, shows that ectopic Scr expression in MN2a induces a strong phenotype (mistargeting to the Scr+ MHD muscle) whereas overexpression in MN3 induces a much smaller effect (****new main Figure 4****). Likewise, ectopic expression of Dfd in MN3 (Dfd-) in induces mistargeting to MHE (Dfd+), whereas overexpression of Dfd MN2a causes weaker phenotypes (mostly terminal defects).*
Here we used the comparison of two MN axon projections with distinct homeocode signatures to compare the mis-expression of a factor with overexpression conditions of the same factor as suggested by the Reviewer for Ubx.
Here we excuse that we did not perform this experiment with Ubx, but instead with Scr and Dfd, because we are more familiar with axon projection in the anterior segments

*Additionally, we performed gain of function experiments for DIPgamma and observed in MN3 (DIPgamma negative) a strong shift towards the upper MHE that is innervated by the DIPgamma expressing MN2a, thereby phenocopying ectopic Dfd expression. As expected, we did not find a phenotype on the MN2a (DIPgamma expressing) projection by DIPgamma overexpression. This experiment demonstrates that the expression of a single Immunoglobulin can induce strong targeting defects. Thus, we were able to find an additional additional instance where ectopic expression of an immunoglobulin phenocopies ectopic expression of an upstream homeodomain TF (**new main Figure 6**).*

- *Finally, we investigated the innervation pattern of our loss of function experiments (Dfd^{RNAi}, hth^{RNAi}, mirr^{RNAi}) in more detail and show that the interference with a single homeodomain TF in the same motoneuron causes distinct and specific wiring defects (**revised main Figure 3**).*

Taken together, the outcome of these gain of function and loss of function experiments are in line with what would be expected from a model whereby motoneurons and their target muscle express the same homeodomain TFs and are mediated by immunoglobulins. Our experiments further indicate that the effects mediated by each of the factors are rather specific, suggesting that a combinatorial code of these factors is required to achieve wiring precision. A more extensive characterization of other neurons is not currently possibly, due to difficulties in microscopically identifying axon projections in this system. In addition, we have to note, that we observe partial penetrance of phenotypes by misregulations of these factors, indicating that a combinatorial code involving homeodomainTFs, Igs, and possibly other factors, is required for synaptic specificity.

4) In total, 1536 motoneurons were sequenced by SMART- Seq2 (Picelli et al., 2014). After filtering, 999 single-cell transcriptomes were retained, thus each biologically unique motoneuronal cell was covered on average 7-fold in our dataset (Supplementary Fig. 1B)" It is unclear with the method section if this is simply the cut-off of reads/genes or if other filters have been used since about 1/3 of cells are lost. Why is this the case? Maybe it's worth to state this in the main text.

Filtering was done exclusively based on a minimum number of 500 genes observed with at least 10 reads per cell. This criterion is now also mentioned in the main text, and established in the field. The remaining cells would mostly have been of sufficient quality to identify main clusters (e.g. twit^{low}, twit^{high} and VUM motoneurons), but a more stringent threshold was required to enable the analysis of the homeo-code, which requires high quality data and high sequencing depth (see also the second point raised by reviewer 1).

5) Do VUMs also follow the AP Hox expression code? It might be interesting to also display (and putatively discuss) this data.

*While the number of VUMs sequenced (40) was too small to systematically delineate a Hox code, homeobox TFs are highly variably expressed in this population (**Response Figure 4**, p value for enrichment of homeobox TFs among highly variable genes within VUMs: $6 * 10^{-4}$). Clustering of only the highly variable homeo-TFs demonstrates, for example, that Scr and Abd-B are expressed in a mutually exclusive manner (**Response Figure 4**).*

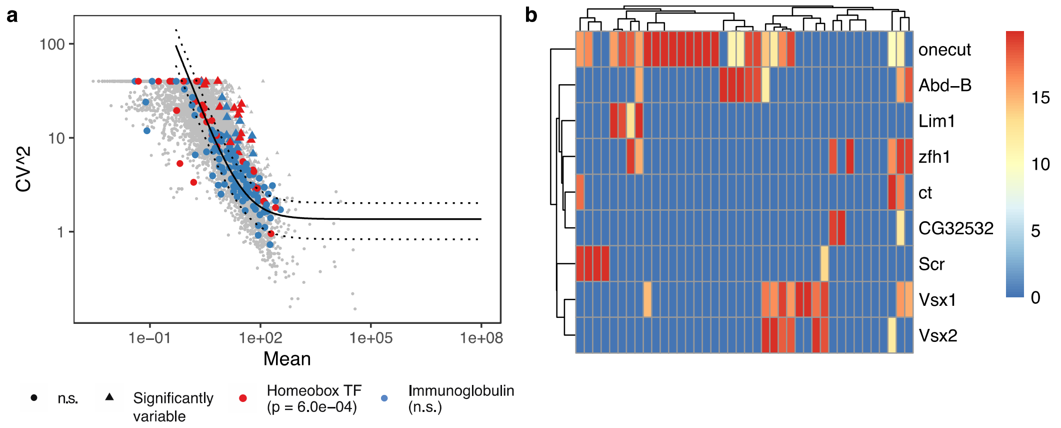

***Response Figure 4**: Analysis of VUM monotneurons.*

***(A)** Identification of highly variable genes using the method by (Brennecke et al., 2013). Scatter plot depicts for each gene the mean expression and squared coefficient of variation across twil^{ow} cells. The solid line indicates the fit, dashed lines the 95% confidence interval. Genes with a significantly elevated variance are shown as triangles, other genes as circles. Different gene classes are color coded. P values shown are from a hypergeometric test for enrichment of the respective gene class among highly variable genes. **(B)** Heatmap depicting the expression of highly variable homeobox TFs (red triangles from panel a) across n=40 VUMs.*

Thank you for sending us your revised manuscript. We have now heard back from the three referees who agreed to evaluate your study. Since Referees #1 and #2 from the previous round of review were not available this time, we were obliged to seek advice from two new experts (Referees #4 and #5). From the comments below, you will see that Referee #3 (who had reviewed the manuscript before) was satisfied with the revisions. The other two referees (who reviewed the manuscript for the first time) still raised a number of issues, while Referee #4 was more supportive. Given the balance of these evaluations, we feel that we can consider a revision of your manuscript if you can address the issues that have been raised.

- We think it is essential to address both referees' concerns regarding the overstatements in the title and manuscript text, and revise the discussion and data interpretation to describe the actual experimental findings more accurately.

- Referee #5 raised additional concerns about the use of OK371-Gal4 to detect MNs, which needs to be convincingly addressed and clarified. We think the proposed experiments and revisions in your preliminary response sound reasonable.

- The other points and comments from Referee #5 need to be clarified/addressed as well.

REFEREE REPORTS

Reviewer #3:

The manuscript has substantially been revised and I feel all points raised have been properly addressed. In particular the clarity of the results as well as the figures are now in an excellent shape. I do not have any further critical comments on the manuscript.

Reviewer #4:

Velten et al.

This study presents a large amount of data characterizing at cellular resolution the transcriptome of postmitotic motoneurons in Drosophila. In the revision, significant technical and descriptive improvements consolidate this valuable data set. The authors focus on homeobox transcription factors and the question how these TFs might control synaptic targeting. Based on these expression results and a few proof-of-principal functional studies the authors suggest that a "code" of homeodomain TFs regulate the expression of a set of Ig-SF receptors that instruct connectivity of the respective neuron-type.

Overall, the study will be of significant value for the scientific community and an important resource. I recommend, therefore, publication. I share, however, concerns with other reviewers regarding the interpretation, which seems disconnected from the actual findings.
It is unclear why the authors refer to the expression of a combination of TFs as a "code". Even less clear is the notion of an "Ig code". Can the authors define what the respective codes are. I do not think that mis-expression of individual genes are suited to test such a far-fetched code-hypothesis. A much more comprehensive manipulation of TF expression would be necessary to begin testing such an idea. For example changing TF combination of cell A into TF combination B. Then showing that this changes Ig-receptor combination A' into and B', with the in vivo confirmation of predicted switches in synaptic connectivity. I do not expect that such a set of experiments is possible currently. In particular, seeing the problematic low penetrance of functional alterations of TFs or Ig-receptors in the genetic experiments.

I much rather suggest to adjust the discussion and interpretation of the results to the precise - and certainly valuable - findings.

Reviewer #5:

Reviewer 1:
Point 1) OK.
Point 2) Still of concern; 7 cells per type of motoneuron is very low and raises concerns regarding reproducibility.
Point 3) Author's response raises new concerns: They claim that using elav-Gal4 they induce transgene expression when the neurons are already differentiating. But this is not true (see my point 4 below).
Point 4) Partly satisfactory, although I share the concern regarding a logical MN code (see my point 9).
Point 5) Partly satisfactory, although I agree that the CSP RNAi phenotypes are weak.

Reviewer 2:
Point 1). Still an issue (see my point 10).
Point 2) Amended in part.
Point 3) Issues remain (see also my point 9).
Point 4) Not amended, rescuing Dfd mutants by DIP-k and DIP-gamma has not been conducted.
Point 5) OK.
Point 6) OK.

Reviewer 3 is re-reviewing, so I will not comment on these points.

Reviewer #5 comments:
The notion that combinations of TFs dictate cell fate was demonstrated already in the 90s and is textbook knowledge by now.

Even in the CNS, numerous studies over the last 2 decades have revealed that combinatorial codes of TFs also can dictate neuronal sub-type identities, and that axon pathfinding and target recognition is one such sub-type feature often governed by TF codes. There are also a number of studies connecting such TF codes to various axon pathfinding genes. The fact that the authors apply the powerful scRNA-seq technology to this topic, although a reasonable way forward, in this case does very little to shine any further light on this issue. Basically, they find that TF codes dictate MN sub-type identify, albeit with gaps in the logic, and that these codes may regulate specific combinations of surface molecules, which may execute some of the pathfinding, although their weak phenotypes argue that many other downstream genes are involved. In addition to that the findings are not novel, there are a number of technical issues with the study (raised by the initial 3 reviewers and some additional ones raised by me). Taken together, I do not think this study is of broad interest, and have concerns about its solidity.

1) OK371-Gal4 is not specific to motor neurons, and there are other glutamatergic cells in the CNS. This is clearly visualised in their figure EV1A, where many cells in the anterior-most parts of the brain (protocerebrum) expresses the OK371-Gal4 marker; a region that has never been described as having any motor neurons. Since they are mostly studying cells that express Hox homeotic genes they are primarily (probably exclusively) studying cells located posterior to the protocerebrum, and these may chiefly be MNs, so perhaps this is of limited concern. But this complicating factor should be outlined in the start of the Results section. For instance, in Fig 2A, they identify cells in the anterior Lab domain as MNs, although they express genes such as Hmx, Rx and ap, which are evolutionarily conserved markers/determinants of specific brain regions and of interneurons. This certainly indicates that they are studying not only MNs.

2) They describe 140 MNs detected in the late embryo with OK371-Gal4. MNs have not been mapped at single-cell resolution in all CNS segments, but extrapolating from what has been described in great detail for thoracic and abdominal segments, there should be an estimated ~1,200 MNs in the stage 17 embryo. They claim (rows 137-148) that the extensive discrepancy between all MNs and those detected by OK371-Gal4 i.e., 140 versus 1200 is due to that OK371-Gal4 only expresses in mature MNs i.e., those that have formed synapses with muscles. However, because many MNs are born quite early, typically between stages 9-12 and rapidly contact their muscle targets, this is a surprising finding, or at least a surprising conclusion by the authors. This raises the question of whether OK371-Gal4 only expresses in mature MNs and/or a subset of MNs. On that note, what is the repertoire of MNs that express OK371-Gal4 at Stage 17? There are a number of markers for MNs that could be used to elucidate this. This is not a trivial detail, because the whole study is based upon using OK371-Gal4 to conduct scRNA-seq, and thereon to map out the TF and CSP codes.

3) The expression analysis of Hox genes along the A-P axis and D-V genes (vvl, mirr) along the D-V axis is given a lot of attention (text, figure space, data) but is quite well-established already. If you scRNA-seq MNs from various parts of the CNS it is anticipated that known patterning genes would express according to the last 35 years of literature.

4) elav-Gal4: the authors correctly refer to elav-Gal4 as pan-neural as opposed to pan-neuronal. While expression of the Elav protein is specific to neurons in the developing CNS, the elav-Gal4 line expresses in most, if not all neuroblasts, and as an affect thereof in both neurons and glia (Berger et al., DevDyn, 2007, 236:3562-3568). They claim that the RNAi is realized earliest at stage 14, and show some evidence for this, which would negate any critique that they may be studying TF gene function in neuroblast as opposed to MNs. However, this still leaves the door open for non-MN-autonomous effects, for instance by RNAi affecting glia that guide MN axons.

5) In the RNAi experiments they use FasII to reveal MN axons and observe changes in muscle innervation. But are the different MNs still there, or could subsets of MNs be removed by apoptosis in the RNAi experiments?

6) Relatedly, the specific MNs under study (MN2a etc) are depicted by an asterisk in all images. It would be re-assuring to actually see these MN cell bodies, and see e.g., Dfd expressed in the cell itself by immunostaining.

7) Rows 192 and 195: "Kania and Jessell, 2003" is probably not the correct reference here, it refers to studies of mouse motor neurons.

8) Split channels for EV2D.

9) 2-3 decades of gene expression analysis, and of course the recent "explosion" of scRNA-seq data has made it clear that different cell types express different combinations of genes. One could hence talk about a "TF code", a "kinase code", a "cadherin code" etc. However, used frivolously the term loses its meaning. In its original inception it was meant to refer to a "functional code" i.e., that AB governed a different cell fate than AC etc. On that note, in Figure 3, when they observe effects in the RNAi for Dfd, hth and mirr, what is the logic of these axon pathfinding changes that justify calling the function of these TFs a "code"? Based upon the expression of the 3 TFs (3C) RNAi of e.g., Dfd should convert MN2a into MN2c, etc. But it is not clear that this is the phenotype they observe.

10) Rows 285-286: "However, it is so far unclear whether sets of homeodomain TFs also instruct developmental programs for precise matching of differentiated MNs and their targets." There are dozens of publications addressing this very question, in worms, flies, zebrafish, chick and mouse. Similarly, the Discussion fails to draw parallels with the many publications describing TF codes for MNs, in worms, flies, zebrafish, chick and mouse, some of which even address TF connections to CSPs.

POINT-BY-POINT RESPONSE TO THE REVIEWERS:

We have now addressed all the points raised by the reviewers. We have included new data, have performed new analyses and have re-written the manuscript based on reviewer's suggestions, which we describe in detail below.

General points:

- We changed the title to „Single-cell sequencing of Drosophila embryonic motoneurons identifies linked homeodomain and Immunglobulin programs critical for synaptic wiring".

- We extensively revised the Discussion and data presentation to eliminate possible overstatements. We included relevant literature, which was previously missing. All changes are highlighted.

- We included new data, which are presented in Figure EV3A-D.

Individual comments to the reviewers' questions:

Reviewer #3: thought the study has been substantially revised and this reviewer felt that all points raised had been properly addressed. This reviewer also highlighted that the manuscript is in an **excellent state**.

Reviewer #4: pointed out that this study will be of **significant value for the scientific community** and an **important resource**. This reviewer had a few concerns that we addressed in the ways outlined.

1) **It is unclear why the authors refer to the expression of a combination of TFs as a "code". Even less clear is the notion of an "Ig code". Can the authors define what the respective codes are. I do not think that mis-expression of individual genes are suited to test such a far-fetched code-hypothesis. A much more comprehensive manipulation of TF expression would be necessary to begin testing such an idea. For example ,changing TF combination of cell A into TF combination B. Then showing that this changes Ig-receptor combination A' into and B', with the in vivo confirmation of predicted switches in synaptic connectivity. I do not expect that such a set of experiments is possible currently. In particular, seeing the problematic low penetrance of functional alterations of TFs or Ig-receptors in the genetic experiments.**

   I much rather suggest to adjust the discussion and interpretation of the results to the precise - and certainly valuable - findings.

We thank the reviewer for her/his valuable input and the appreciation of our data. We would like to state that "code" is (nowadays) often used to specify combinations of genes expressed in individual cells. However, we do agree that "code" is probably more related to a functional code. Thus, we removed completely the term "code" from the manuscript and have replace it with "cell specific expression of …", as this does more accurately reflect our data (lines 59, 61, 168, 428, 478, 479, 563, 682, 691, 825, 828). We have also included a few sentences in the Discussion describing potential reasons for the low penetrance of functional alterations of the TFs or Ig-receptors in genetic experiments (lines 723-730).

Reviewer #5: raised several concerns, which we all addressed as outlined in the following.

1) **OK371-Gal4 is not specific to motor neurons, and there are other glutamatergic cells in the CNS. This is clearly visualised in their figure EV1A, where many cells in the anterior-most parts of the brain (protocerebrum) expresses the OK371-Gal4 marker; a**

region that has never been described as having any motor neurons. Since they are mostly studying cells that express Hox homeotic genes they are primarily (probably exclusively) studying cells located posterior to the protocerebrum, and these may chiefly be MNs, so perhaps this is of limited concern. But this complicating factor should be outlined in the start of the Results section. For instance, in Fig 2A, they identify cells in the anterior Lab domain as MNs, although they express genes such as Hmx, Rx and ap, which are evolutionarily conserved markers/determinants of specific brain regions and of interneurons. This certainly indicates that they are studying not only MNs.

We do agree with the reviewer that not all OK371 expressing cells are motoneurons, which has already been described in the original paper by Mahr and Aberle, 2006. We have now mentioned this very clearly in the Results part as one of the disadvantages of the OK371-GAL4 driver (lines 216-226). However, we would like to highlight that these are only very few cells at stage 16 (see EV1A). In addition, these neurons are not considered in our detailed analysis of homeodomain TFs and Ig molecules, as we exclude these cells from our analysis by focusing only on Hox expressing cells (as described in Materials and Methods). This is the filtering we used to map motoneurons along the AP axis, and it has been stressed more clearly in the text. Although we cannot completely exclude that a very few of these neurons are present in our data, we do not find any indication for this in our data.
In addition, we would like to highlight here that the reviewer stated that the *Hmx* gene is a marker gene of specific brain regions and interneurons. The paper of Wang et al. (2000) showed already in the year 2000 that *Hmx* is also expressed in a few neurons in each hemisegment of the ventral nerve cord in stage 15 and 16 embryos and our single cell study now identifies these Hmx expressing cells outside the brain to be motoneurons. We did not emphasize this finding in the revised manuscript, as the paper of Wang et al (2000) already showed that Hmx expression is not exclusive to the brain as suggested by the reviewer. However, it confirms the value of our data, which will be very useful for the community.
We realized that the description of the disadvantages of the OK371-GAL4 driver could also be moved to a Supplemental note, as we feel it breaks the flow of the manuscript at the beginning of the Results part. We would appreciate here the input of the editor.

2) **They describe 140 MNs detected in the late embryo with OK371-Gal4. MNs have not been mapped at single-cell resolution in all CNS segments, but extrapolating from what has been described in great detail for thoracic and abdominal segments, there should be an estimated ~1,200 MNs in the stage 17 embryo. They claim (rows 137-148) that the extensive discrepancy between all MNs and those detected by OK371-Gal4 i.e., 140 versus 1200 is due to that OK371-Gal4 only expresses in mature MNs i.e., those that have formed synapses with muscles. However, because many MNs are born quite early, typically between stages 9-12 and rapidly contact their muscle targets, this is a surprising finding, or at least a surprising conclusion by the authors. This raises the question of whether OK371-Gal4 only expresses in mature MNs and/or a subset of MNs. On that note, what is the repertoire of MNs that express OK371-Gal4 at Stage 17? There are a number of markers for MNs that could be used to elucidate this. This is not a trivial detail, because the whole study is based upon using OK371-Gal4 to conduct scRNA-seq, and thereon to map out the TF and CSP codes.**

First of all, we would like to highlight that there was a mistake in the previous version, which we now corrected in the text and in the figure: it was not late stage 16/early stage 17 but stage 16 embryos that were used for the single cell sequencing, as this is the stage when motoneurons form connections with their muscle targets (Landgraf et al., 1997).
Coming to the concerns of the reviewer. On the one hand, she/he is concerned about the discrepancy between motoneuronal cell number used in our study (stage 16 embryos) and the number that should be present at the end of embryogenesis (late stage 17), which led to the second concern that we could look only at a subset of motoneurons and not the whole repertoire of motoneurons, which he/she thinks could compromise the study.

A) Discrepancy between cell numbers (140 vs 1200):

The OK317-GAL4 driver starts to induce gene expression in stage 16 (when motoneurons from connections with their muscle targets, which is for example described in Landgraf et al., (1997), the expression gets strongly increased in stage 17 (shown in our Figure EV1A) and will in the end label all motoneurons at the end of embryogenesis (late stage 17) as shown by Mahr and Aberle (Gene Expr Patterns, 2006, see attached pdf).

1) We now show OK371-GAL4 driven RFP expression in stage 16 embryos (the stage we used for the single cell sequencing) and in late stage 17 embryos to demonstrate the increased number of RFP expressing cells in stage 17. In addition, we also quantified the numbers of RFP expressing cells per hemisegment in both stages to highlight the increase in numbers and to show that at the end of stage 17 all MNs are labelled by the OK371-GAL4 driver. We used the quantification per abdominal hemisegment, as this is the standard measurement for motoneurons in the field. Our quantification showed that on average 35 cells per abdominal hemisegment are labelled by the OK371-GAL 4 driver at late stage 17, which is in line with the reported number of total motoneurons per hemisegment (for example Landgraf *et al*, 1997; Landgraf & Thor, 2006) and the number detected by Mahr and Aberle (2006). In stage 16 embryos, about 7 to 8 motoneurons per abdominal hemisegment are labelled by the OK371 driver. We now show these data in Fig. EV3A and describe them in the manuscript (lines 221-223).
2) We would like to highlight here that Mahr and Aberle had already shown that the OK371-GAL4 driver labels all glutamatergic motoneurons at the end of embryogenesis (Mahr and Aberle, 2006, Figure 5). Thus, we are surprised that the reviewer was concerned about the use of this GAL 4 driver. This is indeed the only GAL4 driver labelling all motoneurons, which is the reason why this driver was used for this study.

B) Concern that not all motoneuronal subtypes are present in the 140 motoneurons expressing GFP under the control of the OK371-GAL4 at stage 16 (the stage used for the single cell sequencing):

1) To confirm that all motoneuronal subtypes are present in our data set, which was generated from about 140 motoneurons per stage 16 embryo, we analysed known motoneuronal markers in our single-cell sequenced cells, which is shown below. We used all the known marker genes and their co-expression to define the two major subtypes, the dorsally projecting motoneurons (dMNs) and ventrally projecting motoneurons (vMNs) (for example Landgraf *et al*, 1997; Landgraf & Thor, 2006). The analysis was done on 758 cells taken from the *twit*^{low} cluster (see Fig 1B), cells were assigned as dMNs or vMNs by computing expression scores on dMN and vMN markers. This analysis very clearly shows that the two major motoneuron subpopulations are present in our dataset, even quantitatively at their expected ratios (Fig EV3B, EV3D). In addition, it very nicely confirms that we do see co-expression of known markers in the different subpopulations in the expected rations (Fig. EV3C).
2) In addition, we would like to emphasize that it was absolutely critical for this study to sequence motoneurons at stage 16, as they then from their connections with the muscles. We disagree with the reviewer that connections between muscles and motoneurons are already formed early in embryogenesis after the motoneurons are born (stage 9 to 12), as we only observed interaction late in embryogenesis at the time when the OK371-GAL4 driver starts to be active.

3) The expression analysis of Hox genes along the A-P axis and D-V genes (vvl, mirr) along the D-V axis is given a lot of attention (text, figure space, data) but is quite well-established already. If you scRNA-seq MNs from various parts of the CNS it is anticipated that known patterning genes would express according to the last 35 years of literature.

We deliberately dedicated attention to these points in order to demonstrate the high quality of our data: It recapitulates 35 years of literature and allows to see individual previous findings in a larger

perspective. A major point for using these markers and their known expression along the major body axes was to spatially map the motoneurons in Drosophila embryos to know the location of each motoneuron. Using this approach, we now know for each motoneuron not only its transcriptome but importantly its precise location, which is fundamental for all analyses shown in figure 2-8 and the functional studies. Furthermore, we would like to highlight here that although candidate homeodomain TFs and cell surface molecule encoding genes have been studied in the neuromuscular system, which has led to important conclusions, so far a comprehensive analysis of the system was missing. This is critical to understand the complexity, which will now set the basis to resolve the redundancy and specificity.

4) **elav-Gal4: the authors correctly refer to elav-Gal4 as pan-neural as opposed to pan-neuronal. While expression of the Elav protein is specific to neurons in the developing CNS, the elav-Gal4 line expresses in most, if not all neuroblasts, and as an affect thereof in both neurons and glia (Berger et al., DevDyn, 2007, 236:3562-3568). They claim that the RNAi is realized earliest at stage 14, and show some evidence for this, which would negate any critique that they may be studying TF gene function in neuroblast as opposed to MNs. However, this still leaves the door open for non-MN-autonomous effects, for instance by RNAi affecting glia that guide MN axons.**

We do agree with the reviewer that we cannot completely exclude non-autonomous effects by using the elav-GAL4 driver. However, we also see similar phenotypes with the motoneuron-specific OK6-GAL4 driver. Thus, we now state in lines 502-503: "…. that the phenotypes were primarily due to knockdown of gene expression in MNs and not or to a minor extent caused by non-autonomous effects from other cell types."

5) **In the RNAi experiments they use FasII to reveal MN axons and observe changes in muscle innervation. But are the different MNs still there, or could subsets of MNs be removed by apoptosis in the RNAi experiments?**

In the RNAi experiments, we analyzed selected motoneurons based on their homeodomain and Ig gene expression with a focus on the MN2a and MN3. Projections from these motoneurons are clearly visible by the Fas2 staining in the knock-down experiments, and we observed mistargeting defects. This clearly shows that the motoneurons under study are still there and not removed by apoptosis (otherwise the axons would degenerate). This is now also shown in Response Figure 1, lower panel (see point 6).

6) **Relatedly, the specific MNs under study (MN2a etc) are depicted by an asterisk in all images. It would be re-assuring to actually see these MN cell bodies, and see e.g., Dfd expressed in the cell itself by immunostaining.**

We show this now exemplarily in Response Figure 1 below, which could be included in the manuscript. As you can see in this Figure, the cell projecting its axon (highlighted by FasII staining, yellow) to the MHE expresses Dfd (purple) (upper and central panel). The lower panel shows that cells devoid of one homeodomain TF, in this case hth, are still present and do not die, as the Dfd expressing cell body is still present and projecting its axon, in this case to the LR muscle.

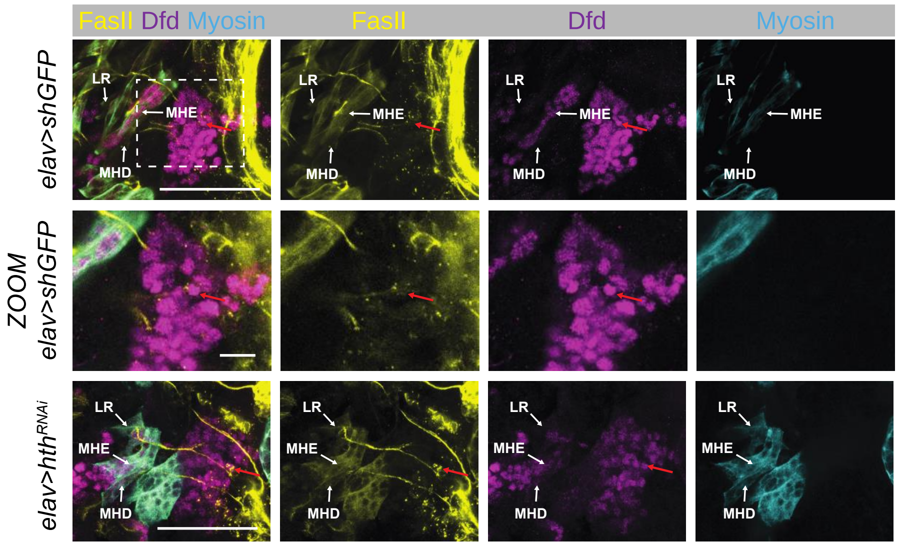

Response Figure 1: Confocal images of stage 17 embryonic heads highlighting expression of Dfd (purple), Myosin in muscles (blue) and FasII in axonal projections (yellow) in control (*elav>shGFP*) animals and after RNA interference with the homeodomain TF gene *hth*. A zoom on the projection (FasII staining) of MN2a to the MHE and MHD muscle of an early stage 17 *Drosophila* embryo are shown (central panel).

7) Rows 192 and 195: "Kania and Jessell, 2003" is probably not the correct reference here, it refers to studies of mouse motor neurons.

We agree with the reviewer and removed the reference.

8) Split channels for EV2D.

We have adjusted the figure.

9) 2-3 decades of gene expression analysis, and of course the recent "explosion" of scRNA-seq data has made it clear that different cell types express different combinations of genes. One could hence talk about a "TF code", a "kinase code", a "cadherin code" etc. However, used frivolously the term loses its meaning. In its original inception it was meant to refer to a "functional code" i.e., that AB governed a different cell fate than AC etc. On that note, in Figure 3, when they observe effects in the RNAi for Dfd, hth and mirr, what is the logic of these axon pathfinding changes that justify calling the function of these TFs a "code"? Based upon the expression of the 3 TFs (3C) RNAi of e.g., Dfd should convert MN2a into MN2c, etc. But it is not clear that this is the phenotype they observe.

The same concern about the use of the term „code" was raised by reviewer #4. We would like to state that "code" is (nowadays) often used to specify combinations of genes expressed in individual cells. However, we do agree that "code" is probably more related to a functional code. Thus, we removed completely the term "code" from the manuscript and have replace it with "cell specific expression of …", as this does more accurately reflect our data. The experiments from Figure 3 and 4 do reveal highly specific effects, e.g. RNAi of Dfd and ectopic expression of Lab target MN2a to the anterior (lab+) MN, which is usually innervated by MN2c. In terms of wiring, the experiment therefore does "convert" MN2a into MN2c. Early steps of neuronal specification are not the focus of our study, hence it is difficult to define a "conversion".

10) **Rows 285-286: "However, it is so far unclear whether sets of homeodomain TFs also instruct developmental programs for precise matching of differentiated MNs and their targets." There are dozens of publications addressing this very question, in worms, flies, zebrafish, chick and mouse. Similarly, the Discussion fails to draw parallels with the many publications describing TF codes for MNs, in worms, flies, zebrafish, chick and mouse, some of which even address TF connections to CSPs.**

We agree with the reviewer that this sentence was not correct. We removed this sentence (and the sentence before) and replaced it with "After having identified cell specific combinations of homeodomain TFs to determine motoneuronal heterogeneity, we wanted to test their contribution to synaptic specificity in individual embryonic MNs." (lines 479-481). We now clearly state in the Discussion that homeodomain TFs have been shown before to function in MNs to control their matching to muscles, not only in Drosophila but also in other organism, and included some of the relevant literature (lines 157-160, 696-700).

ACCEPTED **7th Feb 2022**

Thank you again for sending us your revised manuscript. We are now satisfied with the modifications made and I am pleased to inform you that your paper has been accepted for publication.

EMBO PRESS

YOU MUST COMPLETE ALL CELLS WITH A PINK BACKGROUND ↓

PLEASE NOTE THAT THIS CHECKLIST WILL BE PUBLISHED ALONGSIDE YOUR PAPER

Corresponding Author Name: Ingrid Lohmann & Lars Velten

Journal Submitted to: Molecular Systems Biology

Manuscript Number: MSB-2021-10255

Reporting Checklist For Life Sciences Articles (Rev. June 2017)

This checklist is used to ensure good reporting standards and to improve the reproducibility of published results. These guidelines are consistent with the Principles and Guidelines for Reporting Preclinical Research issued by the NIH in 2014. Please follow the journal's authorship guidelines in preparing your manuscript.

A- Figures

1. Data

The data shown in figures should satisfy the following conditions:

- the data were obtained and processed according to the field's best practice and are presented to reflect the results of the experiments in an accurate and unbiased manner.
- figure panels include only data points, measurements or observations that can be compared to each other in a scientifically meaningful way.
- graphs include clearly labeled error bars for independent experiments and sample sizes. Unless justified, error bars should not be shown for technical replicates.
- if n< 5, the individual data points from each experiment should be plotted and any statistical test employed should be justified
- Source Data should be included to report the data underlying graphs. Please follow the guidelines set out in the author ship guidelines on Data Presentation.

2. Captions

Each figure caption should contain the following information, for each panel where they are relevant:

- a specification of the experimental system investigated (eg cell line, species name).
- the assay(s) and method(s) used to carry out the reported observations and measurements
- an explicit mention of the biological and chemical entity(ies) that are being measured.
- an explicit mention of the biological and chemical entity(ies) that are altered/varied/perturbed in a controlled manner.

- the exact sample size (n) for each experimental group/condition, given as a number, not a range;
- a description of the sample collection allowing the reader to understand whether the samples represent technical or biological replicates (including how many animals, litters, cultures, etc.).
- a statement of how many times the experiment shown was independently replicated in the laboratory.
- definitions of statistical methods and measures:
  - common tests, such as t-test (please specify whether paired vs. unpaired), simple χ2 tests, Wilcoxon and Mann-Whitney tests, can be unambiguously identified by name only, but more complex techniques should be described in the methods section;
  - are tests one-sided or two-sided?
  - are there adjustments for multiple comparisons?
  - exact statistical test results, e.g., P values = x but not P values < x;
  - definition of 'center values' as median or average;
  - definition of error bars as s.d. or s.e.m.

Any descriptions too long for the figure legend should be included in the methods section and/or with the source data.

In the pink boxes below, please ensure that the answers to the following questions are reported in the manuscript itself. Every question should be answered. If the question is not relevant to your research, please write NA (non applicable). We encourage you to include a specific subsection in the methods section for statistics, reagents, animal models and human subjects.

USEFUL LINKS FOR COMPLETING THIS FORM

http://www.antibodypedia.com	Antibodypedia
http://1degreebio.org	1DegreeBio
http://www.equator-network.org/reporting-guidelines/improving-bioscience-research-repor	ARRIVE Guidelines
http://grants.nih.gov/grants/olaw/olaw.htm	NIH Guidelines in animal use
http://www.mrc.ac.uk/Ourresearch/Ethicsresearchguidance/Useofanimals/index.htm	MRC Guidelines on animal use
http://ClinicalTrials.gov	Clinical Trial registration
http://www.consort-statement.org	CONSORT Flow Diagram
http://www.consort-statement.org/checklists/view/32-consort/66-title	CONSORT Check List
http://www.equator-network.org/reporting-guidelines/reporting-recommendations-for-tumo	REMARK Reporting Guidelines (marker prognostic studies)
http://datadryad.org	Dryad
http://figshare.com	Figshare
http://www.ncbi.nlm.nih.gov/gap	dbGAP
http://www.ebi.ac.uk/ega	EGA
http://biomodels.net/	Biomodels Database
http://biomodels.net/miriam/	MIRIAM Guidelines
http://jjj.biochem.sun.ac.za	JWS Online
https://osp.od.nih.gov/biosafety-biosecurity-and-emerging-biotechnology/	Biosecurity Documents from NIH
http://www.selectagents.gov/	List of Select Agents

B- Statistics and general methods

Please fill out these boxes ↓ (Do not worry if you cannot see all your text once you press return)

1.a. How was the sample size chosen to ensure adequate power to detect a pre-specified effect size?	For the single cell transcriptomics experiment: The number of OK371>GFP cells per animal was quantified as 140 cells, corresponding to 70 biologically unique cells due to the bilateral symmetry (see supplementary Figure 1A and C). Taking into account the high variance of single cell RNA-seq measurements, we aimed to cover each biologically unique cell with at least 10 replicates, a number arbitrarily chosen based on experience. Finally, 999 cells were included in the dataset, corresponding to a 14-fold coverage of each biologically unique cell. This strategy is described in the results section.
1.b. For animal studies, include a statement about sample size estimate even if no statistical methods were used.	For genetics experiments with Drosophila embryos, no statistical methods were used to pre-determine sample size.
2. Describe inclusion/exclusion criteria if samples or animals were excluded from the analysis. Were the criteria pre-established?	For the single cell transcriptomics experiment: Pre-established filtering criteria commonly used in the field were used (cell were removed unless they contained at least 10 reads for each of at least 500 genes, and genes were removed unless they were expressed in at least 5 cells with 10 reads each). These criteria are described in the methods section.<br>For microscopy based experiments (phenotyping, validation of the homeocode, etc.), embryos were selected based on stage (late stage 16/early 17 were included), experiments were removed if staining was unsuccessful, or if the orientation of the embryo on the slide was not dorsal-ventral.
3. Were any steps taken to minimize the effects of subjective bias when allocating animals/samples to treatment (e.g. randomization procedure)? If yes, please describe.	No experiments based on differential experimental treatment of genetically identical samples/animals are included in the manuscript
For animal studies, include a statement about randomization even if no randomization was used.	No experiments based on differential experimental treatment of genetically identical samples/animals are included in the manuscript
4.a. Were any steps taken to minimize the effects of subjective bias during group allocation or/and when assessing results (e.g. blinding of the investigator)? If yes please describe.	No blinding was performed
4.b. For animal studies, include a statement about blinding even if no blinding was done	No blinding was performed
5. For every figure, are statistical tests justified as appropriate?	Statistical tests are described in the figure legends and further justified in the main text where appropriate.
Do the data meet the assumptions of the tests (e.g., normal distribution)? Describe any methods used to assess it.	Statistical tests are described in the figure legends and further justified in the main text where appropriate.<br>Statistical methods used in the context of single cell genomics were tools established in the field for this purpose (e.g. Brennecke et al., Nature Methods 2013, Risso et al., Nature Communications 2018).<br>Phenotypes were classified into discrete groups and statistically analyzed using the Fisher test, which does not make specific assumptions regarding normality.<br>qPCR data was analyzed using a t-test, as is common practice in the field (see https://doi.org/10.1186/1471-2105-7-85)

Is there an estimate of variation within each group of data?	Yes, included in all quantitative figures.
Is the variance similar between the groups that are being statistically compared?	Yes

C- Reagents

6. To show that antibodies were profiled for use in the system under study (assay and species), provide a citation, catalog number and/or clone number, supplementary information or reference to an antibody validation profile. e.g., Antibodypedia (see link list at top right), 1DegreeBio (see link list at top right).	Antibodies used in this study are: Mouse anti-Scr (DSHB 6H4.1); Mouse anti-Abd A (all isoforms) (DSHB Ubx/ABD-A FP6.87); Mouse anti-And B (DSHB 1A2E9); Mouse anti-Antp (DSHB 8c11); Mouse anti-FasII (DSHB 1D4); Guinea pig anti-Ubx (Domsch et al. 2019); Guinea pig anti-Dfd (this study); Guinea pig anti-Vnd (Robert Zinzen lab), Goat anti-Abd A (Santa Cruz 27063); Rat anti-ELAV (DSHB 7E8A10); Rat anti-Vvl (W.A Johnson lab, Anderson et al. 1995); Rat anti-GFP (chromotek 3H9); Rat anti-Myosin (abcam ab51098-100); Rabbit anti-Lab (T. Kaufmann lab, Diederich et al.1989); Rabbit anti-GFP (Invitrogen, A11122); Rabbit anti-Hth (Richard Mann lab, McKay et al. 2009).
7. Identify the source of cell lines and report if they were recently authenticated (e.g., by STR profiling) and tested for mycoplasma contamination.	N/A

* for all hyperlinks, please see the table at the top right of the document

D- Animal Models

8. Report species, strain, gender, age of animals and genetic modification status where applicable. Please detail housing and husbandry conditions and the source of animals.	We used the OK371-GAL4 driver (Mahr & Aberle, 2006) crossed to UAS-mRFP to perform single cell sorting of MNs, and the Mhc-TAU-GFP line (Chen & Olson, 2001) for sorting of somatic muscle cells. In order to validate protein expression of specific marker genes, we used the following endogenously GFP tagged fly lines: frq1::GFP (BL:60284), mirr::GFP (BL:68183) and lab::GFP (BL:66551) lines. For genetic experiments, we used the pan-neural elav-GAL4 driver (Luo et al., 1994) and the muscle specific Mef2-GAL4 driver (Ranganayakulu et al., 1998) as main driver. In addition, we combined these two driver lines (elav-GAL4; Mef2-GAL4 driver) for this study. To confirm phenotypes, in MNs, the OK6-GAL4 driver (Mahr & Aberle, 2006) was used in a few examples. The UAS-DfdRNAi was designed for this study using the second generation of TRiP system. The UAS-mirrRNAi (BL:65877), UAS-hthRNAi (BL:34637), UAS-DIPgammaRNAi (BL:80461), UAS-DIPkappaRNAi(BL:31740) were obtained from the Bloomington Stock Center. The UAS overexpression lines UAS-Ubx-HA (Domsch et al., 2019) and UAS-Dfd-HA were designed by Katrin Domsch as described in (Domsch et al., 2019), the UAS-lab and UAS-dpr1 (BL:31740) lines were from the Bloomington Stock Center, and the UAS-DIPgamma (F003086) and UAS-dpr11 (F003032) lines were from the FlyORF collection. Animals are raised according to the guide line in the field.
9. For experiments involving live vertebrates, include a statement of compliance with ethical regulations and identify the committee(s) approving the experiments.	N/A
10. We recommend consulting the ARRIVE guidelines (see link list at top right) (PLoS Biol. 8(6), e1000412, 2010) to ensure that other relevant aspects of animal studies are adequately reported. See author guidelines, under 'Reporting Guidelines'. See also: NIH (see link list at top right) and MRC (see link list at top right) recommendations. Please confirm compliance.	Yes

E- Human Subjects

11. Identify the committee(s) approving the study protocol.	N/A
12. Include a statement confirming that informed consent was obtained from all subjects and that the experiments conformed to the principles set out in the WMA Declaration of Helsinki and the Department of Health and Human Services Belmont Report.	N/A
13. For publication of patient photos, include a statement confirming that consent to publish was obtained.	N/A
14. Report any restrictions on the availability (and/or on the use) of human data or samples.	N/A
15. Report the clinical trial registration number (at ClinicalTrials.gov or equivalent), where applicable.	N/A
16. For phase II and III randomized controlled trials, please refer to the CONSORT flow diagram (see link list at top right) and submit the CONSORT checklist (see link list at top right) with your submission. See author guidelines, under 'Reporting Guidelines'. Please confirm you have submitted this list.	N/A
17. For tumor marker prognostic studies, we recommend that you follow the REMARK reporting guidelines (see link list at top right). See author guidelines, under 'Reporting Guidelines'. Please confirm you have followed these guidelines.	N/A

F- Data Accessibility

18: Provide a "Data Availability" section at the end of the Materials & Methods, listing the accession codes for data generated in this study and deposited in a public database (e.g. RNA-Seq data: Gene Expression Omnibus GSE39462, Proteomics data: PRIDE PXD000208 etc.) Please refer to our author guidelines for 'Data Deposition'.<br><br>Data deposition in a public repository is mandatory for:<br>a. Protein, DNA and RNA sequences<br>b. Macromolecular structures<br>c. Crystallographic data for small molecules<br>d. Functional genomics data<br>e. Proteomics and molecular interactions	Single cell transcriptomics raw data and count tables are available from gene expression omnibus, ID: GSE155578 (for the motoneuronal dataset) and ID: GSE155586 (for the muscle dataset) reviewer access for both datasets: svgvmiqijvobfwz.
19. Deposition is strongly recommended for any datasets that are central and integral to the study; please consider the journal's data policy. If no structured public repository exists for a given data type, we encourage the provision of datasets in the manuscript as a Supplementary Document (see author guidelines under 'Expanded View' or in unstructured repositories such as Dryad (see link list at top right) or Figshare (see link list at top right).	Single cell transcriptomics raw data and count tables are available from gene expression omnibus, ID: GSE155578 (for the motoneuronal dataset) and ID: GSE155586 (for the muscle dataset) reviewer access for both datasets: svgvmiqijvobfwz.
20. Access to human clinical and genomic datasets should be provided with as few restrictions as possible while respecting ethical obligations to the patients and relevant medical and legal issues. If practically possible and compatible with the individual consent agreement used in the study, such data should be deposited in one of the major public access-controlled repositories such as dbGAP (see link list at top right) or EGA (see link list at top right).	NA
21. Computational models that are central and integral to a study should be shared without restrictions and provided in a machine-readable form. The relevant accession numbers or links should be provided. When possible, standardized format (SBML, CellML) should be used instead of scripts (e.g. MATLAB). Authors are strongly encouraged to follow the MIRIAM guidelines (see link list at top right) and deposit their model in a public database such as Biomodels (see link list at top right) or JWS Online (see link list at top right). If computer source code is provided with the paper, it should be deposited in a public repository or included in supplementary information.	NA

G- Dual use research of concern

22. Could your study fall under dual use research restrictions? Please check biosecurity documents (see link list at top right) and list of select agents and toxins (APHIS/CDC) (see link list at top right). According to our biosecurity guidelines, provide a statement only if it could.	No
